# Fates of imine intermediates in radical cyclizations of *N*-sulfonylindoles and ene-sulfonamides

**DOI:** 10.3762/bjoc.11.181

**Published:** 2015-09-17

**Authors:** Hanmo Zhang, E Ben Hay, Stephen J Geib, Dennis P Curran

**Affiliations:** 1 Department of Chemistry, University of Pittsburgh, Pittsburgh, PA 15260, USA

**Keywords:** ene-sulfonamide, imine, radical cyclization, radical fragmentation, spiro-indoles

## Abstract

Two new fates of imine intermediates formed on radical cyclizations of ene-sulfonamides have been identified, reduction and hydration/fragmentation. Tin hydride-mediated cyclizations of 2-halo-*N*-(3-methyl-*N*-sulfonylindole)anilines provide spiro[indoline-3,3'-indolones] or spiro-3,3'-biindolines (derived from imine reduction), depending on the indole C2 substituent. Cyclizations of 2-haloanilide derivatives of 3-carboxy-*N*-sulfonyl-2,3-dihydropyrroles also presumably form spiro-imines as primary products. However, the lactam carbonyl group facilitates the ring-opening of these cyclic imines by a new pathway of hydration and retro-Claisen-type reaction, providing rearranged 2-(2'-formamidoethyl)oxindoles.

## Introduction

**Figure 1:**
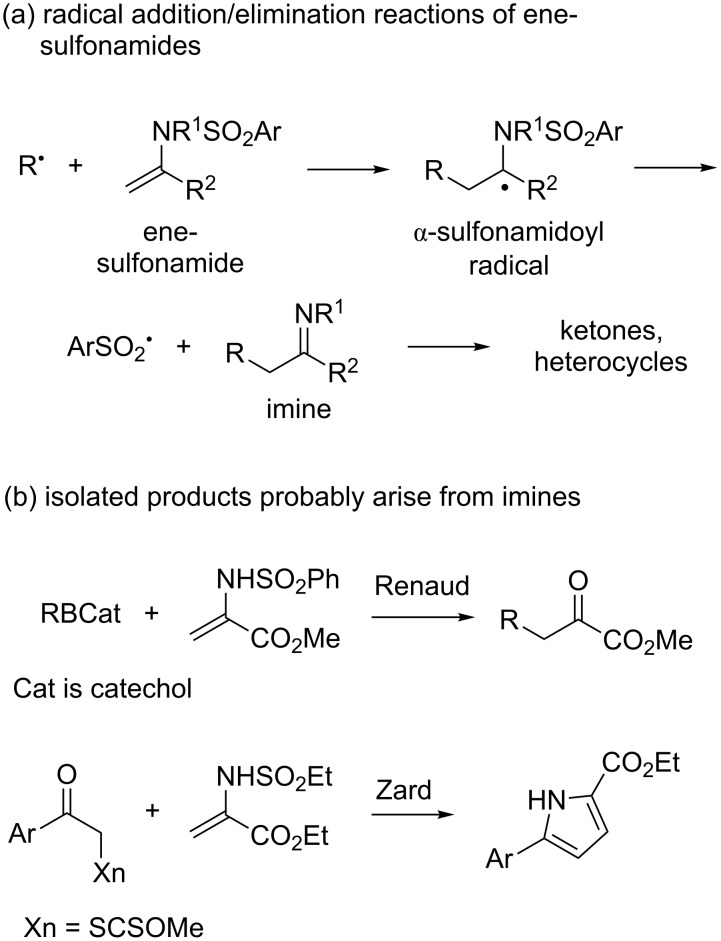
(a) Radical reactions of ene-sulfonamides give diverse isolated products; (b) these products are often derived from transient imine intermediates.

Radical additions and cyclizations of ene-sulfonamides are useful reactions in a number of settings. The primary products of these reactions, α-sulfonamidoyl radicals, are often thought to undergo elimination reactions of sulfonyl radicals to make transient imines [1–3]. In turn, onward reactions of the imines provide assorted products including ketones and nitrogen heterocycles [4–10]. Figure 1a shows a generic addition/elimination reaction of ene-sulfonamides along with example transformations in Figure 1b from Renaud [8] and Zard [7] that probably involve imine intermediates.

**Figure 2:**
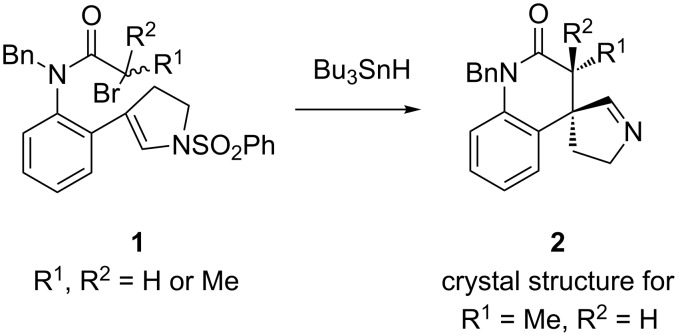
Isolation of stable imines strengthens the case for sulfonyl radical elimination.

Recent work from our group considerably strengthened the case for α-sulfonamidoyl radical elimination reactions. We were able to isolate stable imines from treatment of an assortment of ene-sulfonamides bearing radical precursors [11]. Typical examples are the cyclizations of bromide precursors **1** mediated by Bu_3_SnH (Figure 2). Imines **2** were formed in good yields from primary, secondary and tertiary radical precursors (R^1^, R^2^ = H or Me). The cyclic imine structures **2** were evident from characteristic resonances in the ^1^H and ^13^C NMR spectra, and in one case, from an X-ray crystal structure. Assorted bicyclic and tricyclic imines were formed with both spiro-rings and fused-rings and with varying ring sizes and substituents.

Extending this work, we have studied radical cyclizations of several ene-sulfonamides and *N*-sulfonylindoles. The substrates were chosen to provide imines with spirocyclic indoline and indolone substituents. We discovered that these imines are rather reactive, being either reduced or undergoing an unusual hydration and retro-Claisen-type reaction of an amide. Here we report the results of these experiments, which add to the types of products that can be formed from radical reactions of ene-sulfonamides.

## Results and Discussion

### Cyclizations of *N*-sulfonylindoles

**Scheme 1:**
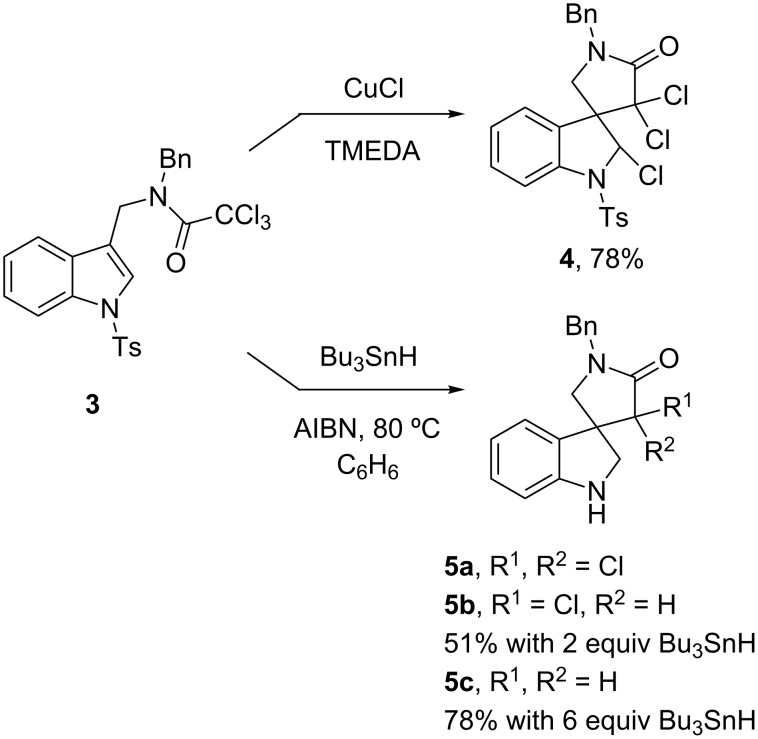
Cyclizations of *N*-sulfonylindole **3** occur with retention or elimination of the sulfonyl group depending upon conditions.

Radical cyclizations of 3-substituted *N*-alkylindoles provide spirocyclic indolines [12–13]. Nicolaou used this reaction as a key step in a synthesis of the alkaloid aspidophytine published in 2008 [14]. In 2005, Stevens and coworkers reported that treatment of trichloroacetamide-substituted *N*-sulfonylindole **3** under conditions for copper-catalyzed atom transfer cyclization provided chlorine transfer product **4** with the *N*-sulfonyl group intact (Scheme 1) [15]. In this reaction, the putative α-sulfonamidoyl radical presumably abstracts a chlorine atom from the catalyst faster than it eliminates the sulfonyl radical.

To learn whether such an elimination could occur under other conditions, we prepared substrate **3** and treated it with varying amounts of tributyltin hydride. Syringe pump addition experiments gave mixtures of various reductive dechlorination products, but fixed concentration experiments gave more tractable results. The reaction of **3** (0.01 M solution) with 2 equiv of tributyltin hydride gave a mixture of products from which the major product **5b** was isolated in 51% yield by flash chromatography. The probable precursor of monochloride **5b** is dichloride **5a**, which was also apparent as a minor product in the crude reaction mixture from this experiment but was not isolated in pure form. To simplify the situation with respect to reductive dechlorination, we treated **3** with excess tributyltin hydride (6 equiv). This gave a relatively clean crude product mixture from which the fully reduced spiroindoline **5c** was isolated in 78% yield by flash chromatography. These results suggest that the α-sulfonamidoyl radical resulting from the 5-*exo-trig* cyclization indeed undergoes elimination to form an imine, but that this cyclic imine is directly reduced by tributyltin hydride by either a radical or ionic pathway.

**Scheme 2:**
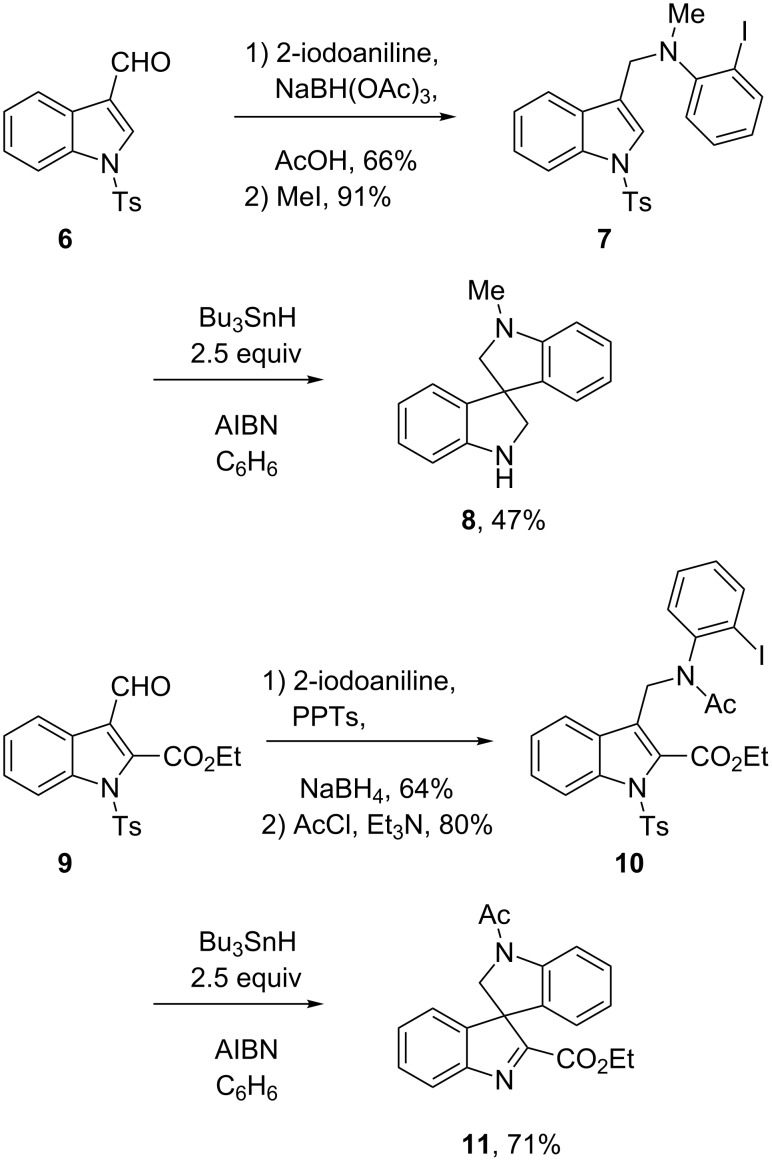
Aryl radical cyclization to *N*-sulfonylindoles.

We next studied two aryl radical cyclizations of *N*-sulfonylindoles, as shown in Scheme 2. Substrate **7** was made in two steps by reductive amination of *N*-tosylindole aldehyde **6** with 2-iodoaniline (66%), followed by methylation of the aniline nitrogen atom (91%). Substrate **10** with the ester on C2 of the indole was chosen to learn if a more stabilized radical intermediate would still eliminate the *N*-tosyl group. Reductive amination of **9**, 2-iodoaniline, PPTS (pyridinium *p*-toluenesulfonate) and NaBH_4_ (64%) was followed by N-acylation of the aniline nitrogen atom (80%) provided the target precursor **10**.

The reaction of **7** with 1 equiv of tributyltin hydride was incomplete, but the use of 2.5 equiv of tributyltin hydride at fixed concentration ([**7**] = 0.01 M) led to complete conversion after 1 h of heating. Solvent evaporation and flash chromatography provided spiro-3,3'-biindoline **8** in 47% isolated yield. As with the formation of **5a–c** in Scheme 1, an imine is presumably formed by aryl radical cyclization and then sulfonyl radical elimination, but is rapidly reduced by the tin hydride reagent.

In contrast, cyclization of 2-ester-substituted indole **9** under the same conditions provided cyclic imine **11** even though excess tributyltin hydride (2.5 equiv) was again used. Imine **11** is a stable compound that was isolated as a clear oil in 71% yield following flash chromatography.

**Figure 3:**
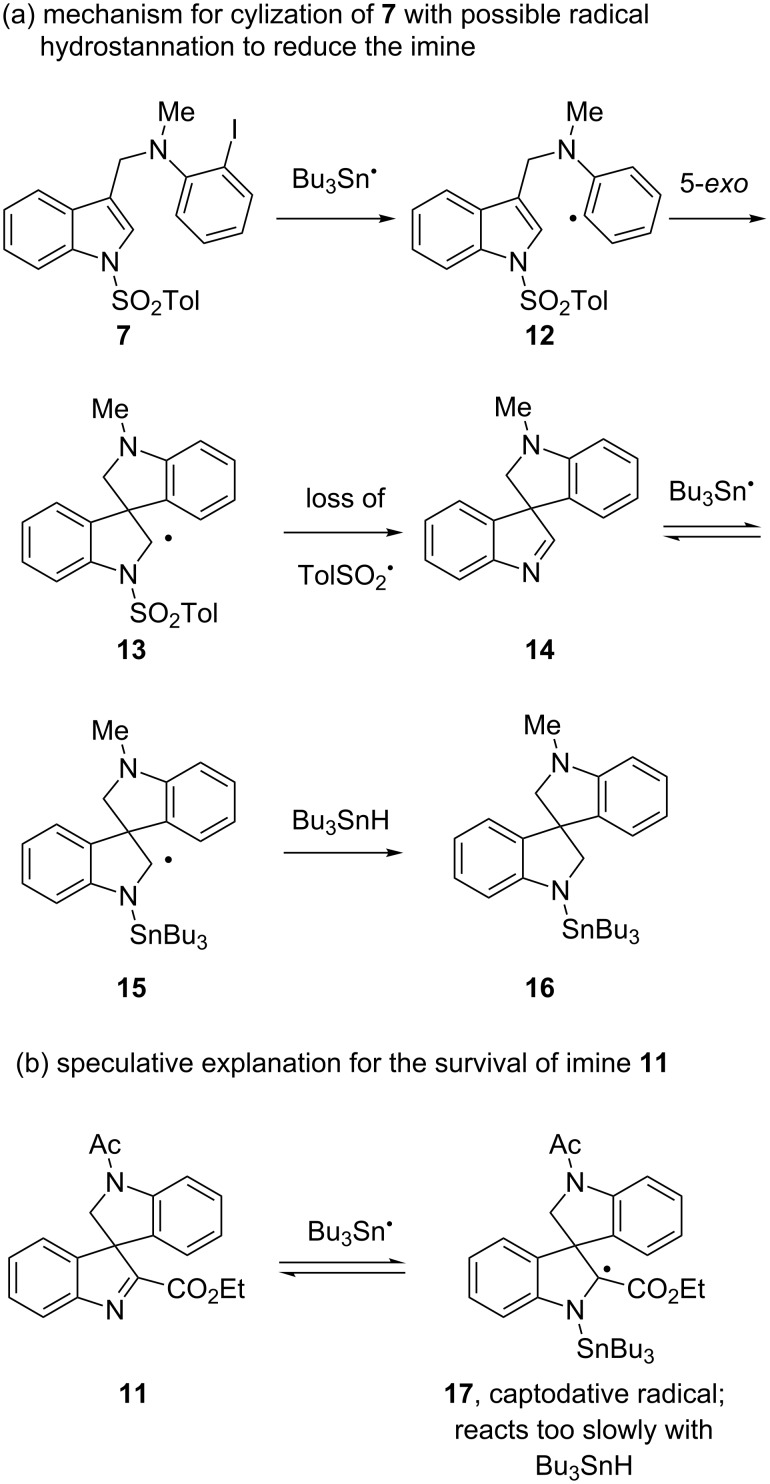
Mechanistic aspects of cyclizations shown in Scheme 2; (a) mechanism for formation of **7**; (b) possible reason for survival of **11**.

Mechanistic aspects of these cyclizations are shown in Figure 3. Precursor **7** provides aryl radical **12**, which in turn undergoes 5-*exo* cyclization to form α-sulfonamidoyl radical **13**. This presumably eliminates tosyl radical (TolSO_2_^•^) to give imine **14**. The difference between reactive imines like **14** in Figure 3 and stable imines like **2** (Figure 2) in the prior work is that the former imines have *N*-aryl groups while the latter have *N*-alkyl groups. This suggests that the *N*-aryl groups promote imine reduction.

We speculate based on the two different types of products isolated from **6** and **9** that the imine **14** is reduced by radical hydrostannation to give **16** via **15**. Finally, protodestannylation of **16** provides isolated product **8**. Imine **11** with the ester substituent on C2 is formed by a similar sequence of reactions as **14**. However, tin radical addition to **11** produces a captodative radical **17** that may be too stable to rapidly abstract a hydrogen atom from tin hydride (Figure 3b). Thus, imine **11** survives to isolation.

That said, we cannot rule out ionic (hydride-type) reduction of imine **14** by tin hydride. Simple imines are not easily reduced by Bu_3_SnH alone, but ionic reductions can be effected by adding Lewis bases (such as HMPA) or by using Lewis acidic tin hydrides (such as Bu_2_SnClH) [16–17]. Potential catalysts of hydride reduction such as benzenesulfinic acid (PhSO_2_H) and tributyltin benzenesulfinate (PhSO_2_SnBu_3_) could form under these conditions [11].

The imine reduction mechanism aside, the take home message is *N*-sulfonylindoles undergo both radical cyclization and elimination; however, the resulting spirocyclic *N*-arylimines may be susceptible to further reduction to the amine depending on the carbon substituent of the imine. In contrast, related spirocyclic imines with *N*-alkyl (rather than *N*-aryl) groups are not readily reduced under the same conditions [11].

### Cyclizations of aniline derivatives of ene-sulfonamides

**Figure 4:**
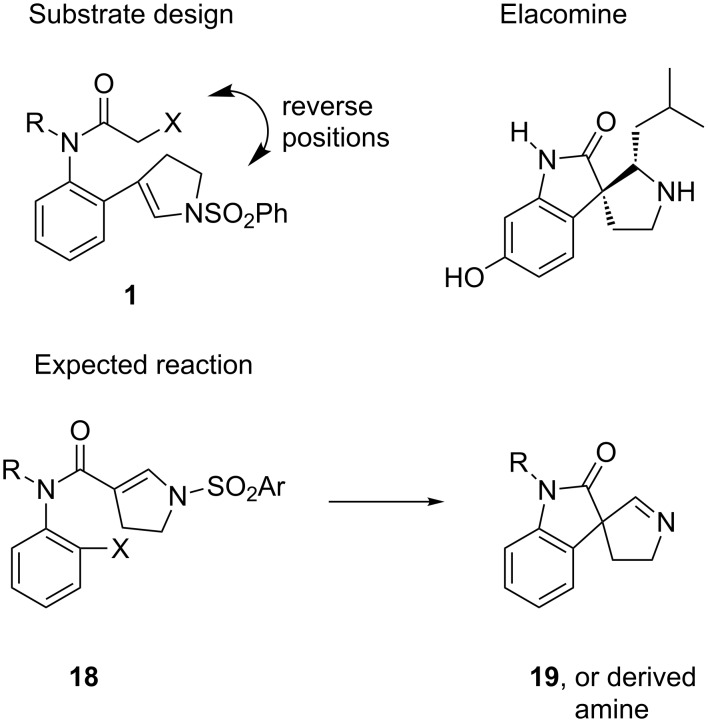
Substrate design by swapping radical precursor and acceptor.

We next studied cyclizations of several aniline analogs of ene-sulfonamides as summarized in Figure 4. The generic substrate **18** was loosely designed based on the original substrates **1** by swapping the locations of the radical precursor (halide) and the radical acceptor (ene-sulfonamide). The expected products of these reactions, imines like **19**, could possibly be used to make spirocyclic oxindole natural products like coerulescine [18], horsfiline [19–23] and elacomine [24] (whose structure is shown in Figure 4). Although the success of the radical cyclization could be projected based on prior work of Jones with *N*-benzylated compounds [19], the subsequent chemistry took a new turn, providing neither an imine nor an amine.

**Scheme 3:**
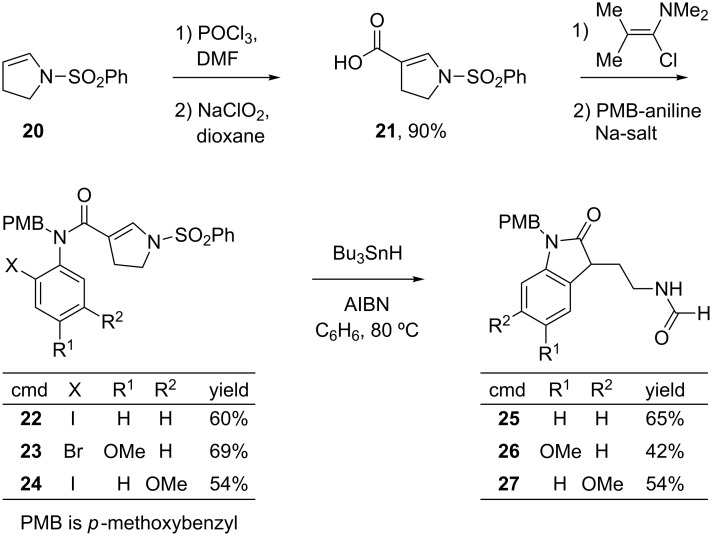
Synthesis and cyclization of precursors **22**–**24**.

The acid **21** needed for the radical precursors was made in high yield as shown in Scheme 3. Vilsmeier–Haack formylation [25] of ene-sulfonamide **20** was followed by sodium chlorite oxidation [26] of the resulting aldehyde (90% yield over two steps). The radical precursors **22**–**24** were readily made in 54–69% yield by treatment of acid **21** with Ghosez reagent (1-chloro-*N,N*,2-trimethyl-1-propenylamine) [27] followed by addition of the sodium salt of the corresponding 2-haloaniline (generated in situ by reaction with NaN(TMS)_2_). The needed *N*-PMB haloanilines were readily available in two or three steps as detailed in the Supporting Information File 1 (PMB is *p*-methoxybenzyl).

Syringe pump addition of a solution of Bu_3_SnH and AIBN to a solution of **22** consumed the starting material, providing a major product that was neither the imine nor the derived amine. The product, isolated by flash chromatography in 60% yield, was soon identified by 1D and 2D NMR experiments, IR and HRMS as 3-(2-formamidoethyl)-2-oxindole **25**.

**Figure 5:**
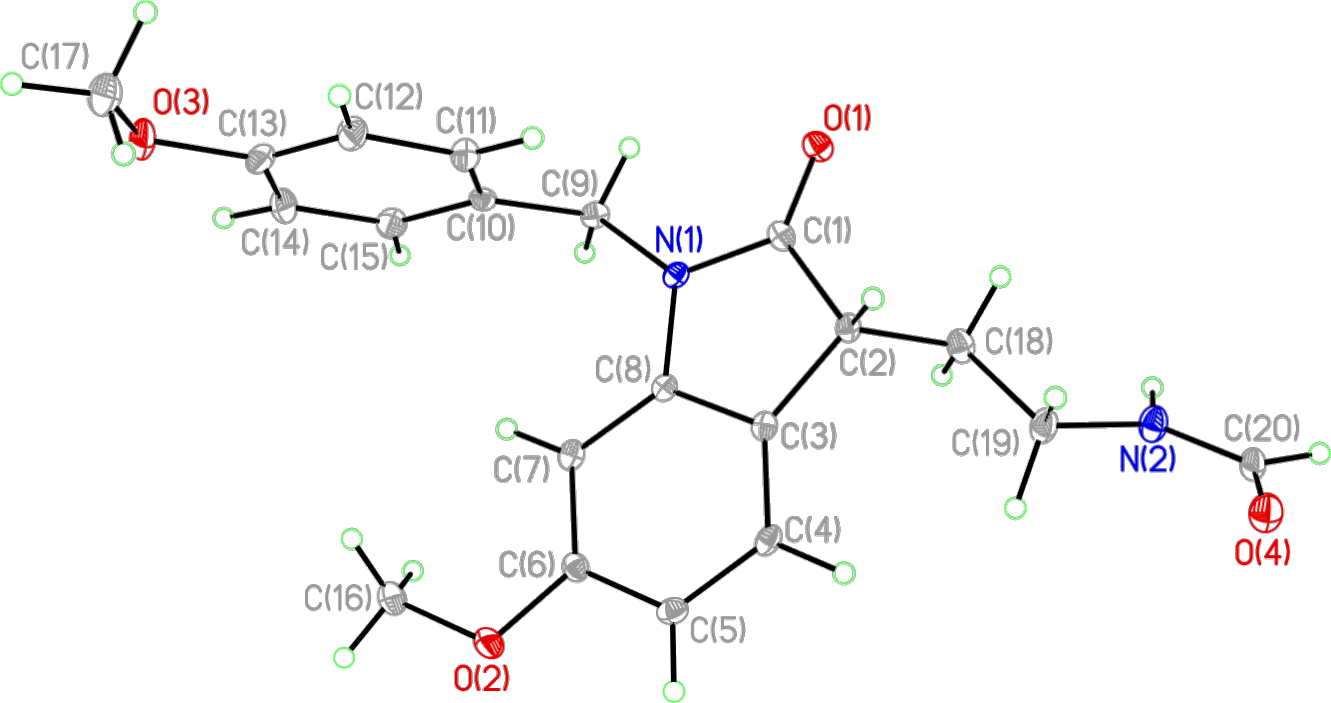
ORTEP representation of the crystal structure of **27**.

The cyclizations of **23** and **24** gave comparable results. The corresponding formamides **26** and **27** were isolated in 42% and 54% yields, respectively. All three formamides are white solids with high melting points (>200 °C), and the crystal structure of **27** was solved to make the structure assignment ironclad. An ORTEP representation of this structure is shown in Figure 5.

**Figure 6:**
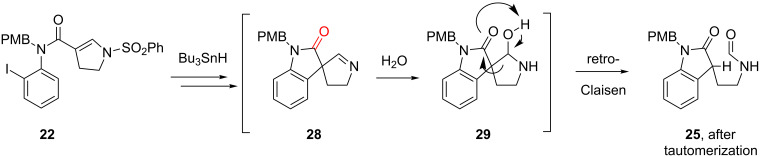
Proposed hydration/retro-Claisen path to formamides.

A likely pathway from **22** to **25** is shown in Figure 6. Radical cyclization and sulfonyl radical elimination give the imine **28**, which then apparently undergoes rapid hydration to hemiaminal **29** followed by fragmentation and enol/lactam tautomerization to give formamide **25**. We show here direct fragmentation of **29**, in what is essentially the lactam/formamide version of a retro-Claisen (or retro-Dieckmann reaction). However, it is also possible that hemiaminal **29** opens in the other direction (with C–N bond cleavage) to give an α-formyl-γ-amino lactam, which then undergoes C-to-N transformylation.

The analysis of the crude reaction mixtures by TLC showed that the spots of formamides **25**–**27** were present early and grew in intensity with time as the intensity of the spots of precursors **22**–**24** faded. This suggests that the formamides are forming during the reaction course, not during the flash chromatography. This conclusion was supported by an experiment with Bu_3_SnH conducted in C_6_D_6_ with direct NMR analysis. The resonances of the precursor **23** were gradually replaced by those of the formamide **26**. No resonances attributed to the intermediate imine or hemiaminal were identified. Likewise, we tried to trap **28** in situ with both isobutylmagnesium bromide (see elacomine in Figure 4), sodium cyanide [28], and sodium borohydride, but in each case got formamide **25** in roughly the same yield as when no additive was present.

The source of water for the imine hydration is not entirely clear. A case could be made for adventitious water in the small scale NMR experiment, but not in the preparative experiments. These were conducted at about 100 mg scale with benzene taken from an activated alumina column. The primary sulfur product of the reaction is probably benzenesulfinic acid, which is thermally unstable, self-condensing with dehydration [29]. Thus, it is possible that either water or a reactive equivalent of water (such as Bu_3_SnOH) is formed during the reaction.

The key difference between the very stable imines such as **2** (Figure 1) and the sensitive imines such as **28** is the presence of the lactam carbonyl group in **28** (highlighted in red in the structure of Figure 6). This group can both assist the hydration of the imine (by inductive electron withdrawal) and provide for the onward retro-Claisen reaction. Imines like **2** are less prone to hydration and, lacking an enolizable substituent, have no retro-Claisen pathway available.

## Conclusion

Two new fates of imine intermediates formed on radical cyclizations of ene-sulfonamides have been identified. Tin hydride-mediated cyclizations at C3 of *N*-sulfonylindoles to make spiro rings occur with ejection of the sulfonyl radical. When the indole C2 substituent is hydrogen, this imine is reduced in situ to a spiro-indoline, most likely by a radical hydrostannation. When the C2 substituent is an ester, the imine is isolated.

Tin hydride-mediated cyclizations of aniline substituted dihydropyrroles **22**–**24** also presumably form spiro-imines as primary products. However, the lactam carbonyl group of these imines opens a new pathway of hydration and retro-Claisen-type reaction, providing rearranged 3-(2'-formamidoethyl)-2-oxindoles. These transformations add new dimensions to the growing radical chemistry of ene-sulfonamides.

## Supporting Information

File 1 Experimental procedures, compound characterization data, and copies of NMR spectra.

File 2 Chemical information file of compound **27**.
