# Supplementary material for: Fates of imine intermediates in radical cyclizations of N-sulfonylindoles and ene-sulfonamides
Source: Beilstein J Org Chem. 2015 Sep 17;11:1649–55. doi: 10.3762/bjoc.11.181 (PMC4660904; doi:10.3762/bjoc.11.181)

Supporting Information

for

Fates of imine intermediates in radical cyclizations of *N*-sulfonylindoles and ene-sulfonamides

Hanmo Zhang, E. Ben Hay, Stephen J. Geib and Dennis P. Curran*

Address: Department of Chemistry, University of Pittsburgh, Pittsburgh, PA 15260, USA

Email: Dennis P. Curran - curran@pitt.edu

*Corresponding author

Experimental procedures, compound characterization data, and copies of NMR spectra

Table of Contents

General remarks..S2

Experimental procedures and compound characterization S3

References..S20

Copies of spectra S21

General Remarks: Chemicals and solvents were purchased from commercial suppliers and used as received, except as follows. Dichloromethane, THF, ether, and toluene were dried by passing through an activated alumina column. All reactions were carried out under an inert atmosphere of dry argon, unless otherwise indicated.

All reactions were followed by TLC or ^{1}H NMR spectroscopy. TLC visualizations were performed by illumination with a UV lamp (254 nm) or staining with a phosphomolybdic acid solution in ethanol and heating. Flash chromatographies were performed with a automated flash chromatography instrument and prepacked columns containing 230-400 mesh silica gel.

^{1}H NMR spectra were recorded at 300, 400, 500 and 600 MHz in $CDCl_3$, and chemical shifts were measured relative to tetramethylsilane (δ 0.00 ppm) or residual solvent peak (δ 7.26 ppm). ^{13}C NMR spectra were recorded at 75, 100, 125, 150 MHz in $CDCl_3$, and chemical shifts were measured relative to residual solvent peak (δ 77.0 ppm). Unless otherwise noted, NMR spectra were recorded at 293 K. The following abbreviations were used to describe coupling: s = singlet, d = doublet, t = triplet, q = quartet, m = multiplet, br = broad. IR spectra were recorded as thin films ($CHCl_3$) or neat on NaCl plates on an FTIR spectrometer.

Aldehyde **6** was prepared using published procedures, and the spectral data matched the reported ones.[1] 1-(Phenylsulfonyl)-2,3-dihydro-1*H*-pyrrole **20** was prepared using published procedures.[2]

Experimental Procedures and Compound Characterization

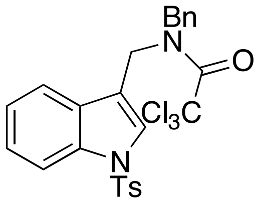

***N*-Benzyl-2,2,2-trichloro-*N*-((1-tosyl-1*H*-indol-3-yl)methyl)acetamide (3)**: The title compound was prepared as a mixture of 2.5:1 rotamers: ^{1}H NMR (300 MHz, $CDCl_3$) δ major rotamer: 4.87 (s, 2H), 4.73 (s, 2H); minor rotamer: 4.99 (s, 2H), 4.66 (s, 2H); overlapping signals: 8.04 (d, J = 7.8 Hz, 1H), 7.78 (d, J = 7.8 Hz, 2H), 7.45−7.12 (m, 10H), 2.37 (s, 3H). The above spectral data were identical to those reported.[3]

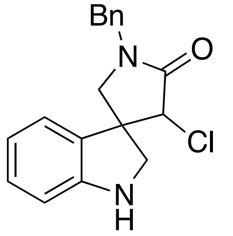

1'-Benzyl-4'-chlorospiro[indoline-3,3'-pyrrolidin]-5'-one (5b): A mixture of tin hydride (110 μL, 0.411 mmol), AIBN (17 mg, 0.102 mmol), and trichloroacetamide **3** (110 mg, 0.205 mmol) in benzene (10 mL) was refluxed for 30 min. The solvent was evaporated and the residue was purified by flash chromatography (silica gel, 30% EtOAc/hexanes) to provide the title compound (33 mg, 51%) as a colorless oil: ^{1}H NMR (300 MHz, $CDCl_3$) δ 7.39−7.26 (m, 5H), 7.18−7.10 (m, 2H), 6.74 (td, J = 7.5 Hz, 1.0 Hz, 1H), 6.68 (d, J = 7.5 Hz, 1H), 4.63 (d, J = 14.4 Hz, 1H), 4.53 (d, J = 14.4 Hz, 1H), 4.45 (s, 1H), 3.77 (brs, 1H), 3.75 (d, J = 10.2 Hz, 1H), 3.63 (d, J = 9.6 Hz, 1H), 3.48 (d, J = 9.6 Hz, 1H), 3.25 (d, J = 10.2 Hz, 1H); ^{13}C NMR (75 MHz, $CDCl_3$) δ

169.3, 151.6, 135.4, 129.5, 128.9, 128.3, 128.1, 126.6, 125.6, 118.8, 110.1, 61.5, 58.6, 55.0, 52.2, 47.2; FTIR (thin film, CH_2Cl_2, cm^{-1}) 3334, 3030, 2922, 2856, 1701, 1607, 1486, 1465, 1257, 742; HRMS (TOF ES) *m/z* cald for $C_{18}H_{17}N_2ONaCl$, 335.0927 $[M+Na]^+$, found: 335.0916.

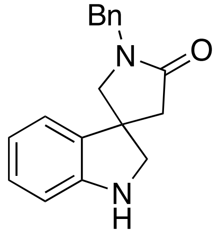

1'-Benzylspiro[indoline-3,3'-pyrrolidin]-5'-one (5c): A mixture of tin hydride (150 μL, 0.560 mmol), AIBN (4 mg, 0.023 mmol), and trichloroacetamide **3** (50 mg, 0.093 mmol) in benzene (10 mL) was refluxed for 30 min. The solvent was evaporated and the residue was purified by flash chromatography (silica gel, 75% EtOAc/hexanes) to provide the title compound (20 mg, 78%) as a colorless oil: ^1H NMR (300 MHz, $CDCl_3$) δ 7.37−7.25 (m, 5H), 7.06 (d, *J* = 8.4 Hz, 2H), 6.75 (t, *J* = 7.8 Hz, 1H), 6.65 (d, *J* = 7.8 Hz, 1H), 4.62 (d, *J* = 14.4 Hz, 1H), 4.41 (d, *J* = 14.4 Hz, 1H), 3.75 (brs, 1H), 3.50 (d, *J* = 14.4 Hz, 1H), 3.48 (d, *J* = 14.4 Hz, 1H), 3.39 (d, *J* = 13.8 Hz, 1H), 3.36 (d, *J* = 13.8 Hz, 1H), 2.87 (d, *J* = 16.8 Hz, 1H), 2.65 (d, *J* = 16.8 Hz, 1H); ^{13}C NMR (75 MHz, $CDCl_3$) δ 173.0, 150.7, 136.2, 132.5, 128.8, 128.6, 128.3, 127.8, 122.1, 119.3, 110.0, 60.6, 58.3, 46.7, 45.8, 44.0; FTIR (thin film, CH_2Cl_2, cm^{-1}) 3336, 2920, 2855, 1680, 1608, 1488, 1256, 745, 702; HRMS (TOF ES) *m/z* cald for $C_{18}H_{18}N_2ONa$, 301.1317 $[M+Na]^+$, found: 301.1304.

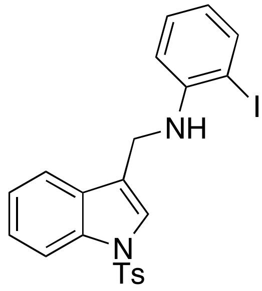

2-Iodo-*N*-((1-tosyl-1*H*-indol-3-yl)methyl)aniline (S1): 2-Iodoaniline (241 mg, 1.10 mmol), acetic acid (60 mg, 1.00 mmol), and sodium triacetoxyborohydride (637 mg, 3.00 mmol) were added to a stirred solution of 1-tosyl-1*H*-indole-3-carbaldehyde **6** (300 mg, 1.00 mmol) in 1,2-dichloroethane (8 mL). After 12 h, the reaction was quenched with a saturated $NaHCO_3$ solution and the aqueous layer was extracted with 1,2-dichloroethane. The combined organic layer was washed with brine, dried with $MgSO_4$, and concentrated *in vacuo*. The residue was purified by flash chromatography (silica gel, 15% EtOAc/hexanes) to give the title compound (330 mg, 66%) as a colorless oil: ^{1}H NMR (300 MHz, $CDCl_3$) δ 8.01 (d, *J* = 8.1 Hz, 1H), 7.73–7.69 (m, 3H), 7.58 (d, *J* = 7.8 Hz, 1H), 7.51 (s, 1H), 7.36 (td, *J* = 7.8 Hz, 1.2 Hz, 1H), 7.27 (m, 1H), 7.22–7.14 (m, 3H), 6.58 (dd, *J* = 8.1 Hz, 1.2 Hz, 1H), 6.50 (td, *J* = 7.5 Hz, 1.2 Hz, 1H), 4.54–4.47 (m, 3H), 2.35 (s, 3H); ^{13}C NMR (75 MHz, $CDCl_3$) δ 146.9, 145.0, 139.1, 135.6, 135.1, 129.9, 129.6, 126.9, 125.1, 124.2, 123.4, 119.9, 119.6, 119.2, 114.0, 111.1, 85.6, 40.2, 21.6; FTIR (thin film, CH_2Cl_2, cm^{-1}) 3394, 3061, 2921, 1589, 1502, 1448, 1367, 1277, 1173, 1121, 1005, 970, 810, 744; HRMS (EI) *m/z* cald for $C_{22}H_{19}N_2O_2SI$, 502.0212 $[M]^+$, found: 502.0198.

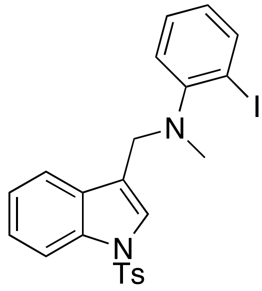

2-Iodo-*N*-methyl-*N*-((1-tosyl-1*H*-indol-3-yl)methyl)aniline (7): A NaHMDS solution (450 μL, 1M in THF) was added dropwise to a solution of aniline **S1** (150 mg, 0.30 mmol) in THF (5 mL) at −78 °C. After 30 min, iodomethane (46 μL, 0.75 mmol) was added dropwise at this temperature. The reaction mixture was then stirred at room temperature for 12 h. A saturated aqueous $NaHCO_3$ solution was added, and the aqueous layer was extracted with diethyl ether. The combined organic layer was washed with brine, dried over $MgSO_4$, and concentrated *in vacuo*. The residue was purified by flash chromatography (silica gel, 20% EtOAc/hexanes) to afford the title compound (140 mg, 91%) as a colorless oil: ^{1}H NMR (300 MHz, $CDCl_3$) δ 7.97 (d, *J* = 8.1 Hz, 1H), 7.88 (dd, *J* = 7.8 Hz, 1.2 Hz, 1H), 7.70 (d, *J* = 8.1 Hz, 2H), 7.60–7.54 (m, 2H), 7.32–7.17 (m, 5H), 7.02 (dd, *J* = 8.1 Hz, 1.2 Hz, 1H), 6.80 (t, *J* = 7.8 Hz, 1H), 4.24 (s, 2H), 2.67 (s, 3H), 2.33 (s, 3H); ^{13}C NMR (75 MHz, $CDCl_3$) δ 153.8, 144.8, 140.3, 135.4, 135.3, 130.6, 129.9, 129.1, 126.8, 125.7, 125.4, 124.8, 123.1, 122.2, 120.6, 119.5, 113.7, 98.6, 51.7, 42.4, 21.7; FTIR (thin film, CH_2Cl_2, cm^{-1}) 3053, 2947, 1596, 1470, 1446, 1367, 1173, 1119, 1093, 978, 812, 747, 702; HRMS (TOF ES) *m/z* cald for $C_{23}H_{22}N_2O_2SI$, 517.0447 $[M+H]^+$, found: 517.0460.

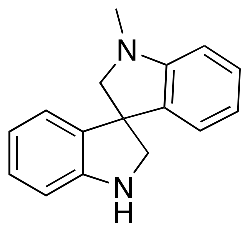

1-Methyl-3,3'-spirobi[indoline] (8): A mixture of aryl iodide **7** (70 mg, 0.136 mmol), tributyltin hydride (91 μL, 0.340 mmol) and AIBN (7 mg, 0.040 mmol) in benzene (13 mL) was refluxed for 20 min. TLC suggested the starting material was not completely consumed. Tin hydride (163 μL, 0.610 mmol) was added in two portions with a catalytic amount of AIBN. After 20 min, the solvent was evaporated, and the residue was purified by flash chromatography (silica gel, 20% EtOAc/hexanes) to provide the title compound (15 mg, 47%) as a colorless oil: ^{1}H NMR (600 MHz, $CDCl_3$) δ 7.18 (t, *J* = 7.8 Hz, 1H), 7.11 (t, *J* = 7.8 Hz, 1H), 7.04 (d, *J* = 7.8 Hz, 1H), 7.01 (d, *J* = 7.8 Hz, 1H), 6.77–6.72 (m, 3H), 6.60 (d, *J* = 7.8 Hz, 1H), 3.78 (d, *J* = 9.0 Hz, 1H), 3.63 (d, *J* = 9.0 Hz, 1H), 3.62 (d, *J* = 9.0 Hz, 1H), 3.27 (d, *J* = 9.0 Hz, 1H), 2.82 (s, 3H); ^{13}C NMR (75 MHz, $CDCl_3$) δ 153.1, 151.4, 135.3, 134.1, 128.4, 128.2, 124.1, 123.6, 119.3, 118.6, 109.7, 107.5, 68.9, 60.3, 54.5, 36.1; FTIR (thin film, CH_2Cl_2, cm^{-1}) 3374, 3046, 2949, 2853, 2805, 1606, 1487, 1463, 1295, 1252, 1151, 1121, 1021, 745; HRMS (EI) *m/z* cald for $C_{16}H_{16}N_2$, 236.1313 $[M]^+$, found: 236.1313.

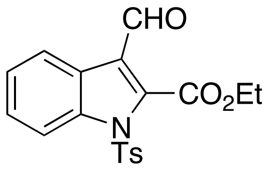

Ethyl 3-formyl-1-tosyl-1*H*-indole-2-carboxylate (9): Ethyl 3-formyl-1*H*-indole-2-carboxylate (150 mg, 0.69 mmol) and tosyl chloride (197 mg, 1.04 mmol) were added to a stirred suspension of Cs_2CO_3 (675 mg, 2.07 mmol) in DMF (4 mL). After 30 min, the reaction was quenched with a

saturated aqueous NH_4Cl solution, and the aqueous layer was extracted with EtOAc. The combined organic layer was washed with brine, dried over $MgSO_4$, and concentrated *in vacuo*. The crude product was purified by flash chromatography (silica gel, 15% EtOAc/hexanes) to afford the title compound (75 mg, 29%) as a colorless oil: ^{1}H NMR (400 MHz, $CDCl_3$) δ 10.16 (s, 1H), 8.29 (d, J = 8.0 Hz, 1H), 8.02–7.98 (m, 3H), 7.46 (td, J = 7.6 Hz, 1.2 Hz, 1H), 7.38 (td, J = 7.6 Hz, 1.2 Hz, 1H), 7.32 (d, J = 8.0 Hz, 2H), 4.61 (q, J = 7.2 Hz, 2H), 2.39 (s, 3H), 1.50 (t, J = 7.2 Hz, 3H); ^{13}C NMR (100 MHz, $CDCl_3$) δ 185.3, 161.1, 146.4, 139.3, 135.3, 134.4, 130.2, 127.9, 127.8, 127.4, 125.7, 125.3, 123.1, 120.9, 114.2, 63.7, 21.9, 14.2; FTIR (thin film, CH_2Cl_2, cm^{-1}) 2924, 2360, 1734, 1680, 1545, 1380, 1321, 1268, 1206, 1179, 1141, 1111, 1086, 1015, 986, 817, 749; HRMS (TOF ES) *m/z* cald for $C_{19}H_{17}NO_5NaS$, 394.0725 $[M+Na]^+$, found: 394.0699.

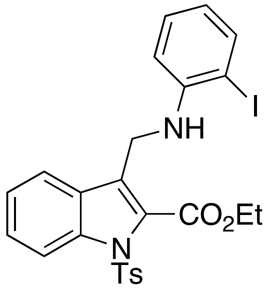

Ethyl 3-((2-iodophenylamino)methyl)-1-tosyl-1*H*-indole-2-carboxylate (S2): A mixture of aldehyde **9** (66 mg, 0.178 mmol), 2-iodoaniline (43 mg, 0.195 mmol), anhydrous $MgSO_4$ (64 mg, 0.533 mmol), and pyridinium *p*-toluenesulfonate (13 mg, 0.053 mmol) in CH_2Cl_2 (2 mL) was stirred for 12 h. The reaction was quenched with a saturated aqueous $NaHCO_3$ solution, and the aqueous layer was extracted with CH_2Cl_2. The combined organic layer was washed with brine, dried over $MgSO_4$, and concentrated *in vacuo*. The resulting crude imine was dissolved in EtOH (3 mL), and $NaBH_4$ (20 mg, 0.533 mmol) was added in one portion. After 3 h, the reaction

was quenched with a saturated aqueous $NaHCO_3$ solution, and the aqueous layer was extracted with EtOAc. The combined organic layer was washed with brine, dried over $MgSO_4$, and concentrated *in vacuo*. The crude product was purified by flash chromatography (silica gel, 15% EtOAc/Hexane) to afford the title compound (65 mg, 64%) as a colorless oil: ^{1}H NMR (500 MHz, $CDCl_3$) δ 8.07 (dd, *J* = 8.0 Hz, 1.0 Hz, 1H), 7.81 (m, 2H), 7.67 (m, 2H), 7.43 (td, *J* = 7.5 Hz, 1.5 Hz, 1H), 7.30 (m, 1H), 7.21 (m, 3H), 6.72 (dd, *J* = 8.0 Hz, 1.5 Hz, 1H), 6.49 (td, *J* = 7.5 Hz, 1.5 Hz, 1H), 4.53 (s, 1H), 4.48 (q, *J* = 7.0 Hz, 1H), 2.34 (s, 3H), 1.39 (t, *J* = 7.0 Hz, 1H); ^{13}C NMR (100 MHz, $CDCl_3$) δ 162.4, 146.8, 145.1, 139.1, 136.8, 134.4, 129.7, 129.6, 129.5, 128.9, 127.2, 126.9, 124.4, 124.3, 120.8, 119.3, 115.5, 110.9, 85.5, 62.6, 39.0, 21.6, 14.0; FTIR (thin film, CH_2Cl_2, cm^{-1}) 3395, 2982, 1724, 1590, 1503, 1451, 1371, 1312, 1266, 1177, 1006, 814, 746; HRMS (EI) cald for $C_{25}H_{23}N_2O_4SI$, 574.0423 $[M]^+$, found: 574.0408.

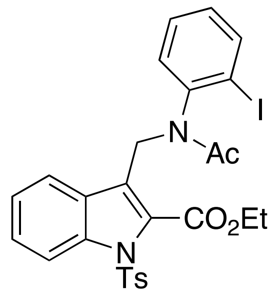

Ethyl 3-((*N*-(2-iodophenyl)acetamido)methyl)-1-tosyl-1*H*-indole-2-carboxylate (10): DMAP (4 mg, 0.03 mmol), pyridine (19 μL, 0.23 mmol), and acetyl chloride (8 μL, 0.11 mmol) were added to a stirred solution of aniline **S2** (33 mg, 0.057 mmol) in CH_2Cl_2 (2 mL) at 0 °C. The reaction mixture was then stirred at room temperature for 12 h. It was diluted with CH_2Cl_2 and a saturated aqueous $NaHCO_3$ solution was added. The aqueous layer was extracted with EtOAc, and the combined organic layer was washed with brine, dried over $MgSO_4$, and concentrated *in vacuo*. The crude product was purified by flash chromatography (silica gel, 25%

EtOAc/hexanes) to afford the title compound (29 mg, 80%) as a colorless oil: ^{1}H NMR (400 MHz, $CDCl_3$) δ 8.00 (d, *J* = 8.0 Hz, 1H), 7.91 (dd, *J* = 8.0 Hz, 1.2 Hz, 1H), 7.82 (d, *J* = 8.0 Hz, 1H), 7.70 (d, *J* = 8.0 Hz, 2H), 7.41 (t, *J* = 8.0 Hz, 1H), 7.30-7.27 (m, 1H), 7.19 (d, *J* = 8.0 Hz, 2H), 7.10 (td, *J* = 7.6 Hz, 1.2 Hz, 1H), 7.02 (td, *J* = 7.6 Hz, 1.2 Hz, 1H), 6.40 (dd, *J* = 7.6 Hz, 1.2 Hz, 1H), 5.74 (d, *J* = 14.4 Hz, 1H), 4.52 (d, *J* = 14.4 Hz, 1H), 4.17–4.01 (m, 2H), 2.35 (s, 3H), 1.78 (s, 3H), 1.24 (t, *J* = 7.2 Hz, 3H); ^{13}C NMR (100 MHz, $CDCl_3$) δ major rotamer: 62.3, 21.6, 13.9; minor rotamer: 60.4, 21.1, 14.2; overlapping signals: 169.9, 161.4, 145.0, 143.0, 140.1, 136.5, 134.5, 131.3, 130.6, 130.0, 129.6, 129.5, 129.0, 127.3, 126.9, 124.6, 122.6, 121.9, 115.1, 100.0, 39.4, 22.8; FTIR (thin film, CH_2Cl_2, cm^{-1}) 2982, 1723, 1664, 1469, 1372, 1313, 1267, 1177, 1017, 762; HRMS (TOF ES) *m/z* cald for $C_{27}H_{25}N_2O_5NaSI$, 639.0427 [M+Na]$^+$, found: 639.0479.

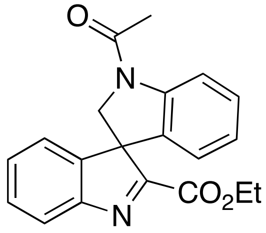

Ethyl 1'-acetylspiro[indole-3,3'-indoline]-2-carboxylate (11): In a sealed tube, a mixture of tin hydride (28 μL, 0.11 mmol), AIBN (1 mg, 0.0060 mmol), and aryl iodide **10** (13 mg, 0.021 mmol) in benzene (2 mL) was refluxed for 30 min. The solvent was evaporated and the residue was purified by flash chromatography (silica gel, 60% EtOAc/hexanes) to provide the title compound (5 mg, 71%) as a colorless oil, in a 5:1 ratio of rotamers: ^{1}H NMR (400 MHz, $CDCl_3$) δ major rotamer: 4.73 (d, *J* = 12.0 Hz, 1H), 2.28 (s, 3H); minor rotamer: 4.80 (d, *J* = 12.0 Hz, 1H), 2.59 (s, 3H); overlapping signals: 8.36 (d, *J* = 8.0 Hz, 1H), 7.90 (d, *J* = 7.6 Hz, 1H), 7.49 (t, *J* = 7.6 Hz, 1H), 7.40 (t, *J* = 7.2 Hz, 1H), 7.32–7.27 (m, 2 H), 6.89 (t, *J* = 7.6 Hz, 1H), 6.42 (d, *J*

= 7.6 Hz, 1H), 4.31–4.29 (m, 3H), 1.26 (t, *J* = 7.2 Hz, 3H); ^{13}C NMR (100 MHz, $CDCl_3$) δ 171.3, 168.2, 160.7, 152.6, 145.2, 144.2, 129.8, 129.5, 128.9, 124.0, 123.9, 122.7, 122.3, 118.1, 64.2, 62.3, 55.4, 24.4. 14.0; FTIR (thin film, CH_2Cl_2, cm^{-1}) 2923, 1722, 1665, 1593, 1482, 1402, 1339, 1267, 1146, 1106, 1021, 754; HRMS (TOF ES) *m/z* cald for $C_{20}H_{18}N_2O_3Na$, 357.1215 $[M+Na]^+$, found: 357.1239.

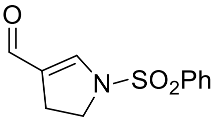

1-(Phenylsulfonyl)-4,5-dihydro-1*H*-pyrrole-3-carbaldehyde (S3): Phosphorous oxychloride (1.21 mL, 12.9 mmol) was added to a solution of DMF (8.4 mL, 10.8 mmol) in CH_2Cl_2 (8.4 mL) at 0 °C. The solution as stirred at 0 °C for 10 min, then ene-sulfonamide **20** (2.50 g, 10.8mmol) in CH_2Cl_2 (10 mL) was added. After 10 min, the solution was poured into saturated aqueous K_2CO_3 (100 mL) and the mixture was stirred vigorously for 1 h. The organic layer was separated, and the aqueous layer was extracted with CH_2Cl_2. The combined extracts were dried over $MgSO_4$ and concentrated. The residue was purification by flash chromatography (20% EtOAc in hexanes) to give the title compound (2.40 g, 94%) as a crystalline solid: mp 76–79 °C. ^{1}H NMR (500 MHz, $CDCl_3$) δ 9.55 (s, 1H), 7.82 (d, J = 7.5 Hz, 2H), 7.66 (t, J = 7.0 Hz, 1H), 7.57 (t, J = 7.5 Hz, 2H), 7.39 (s, 1H), 3.73 (t, J = 9.5 Hz, 2H), 2.78 (t, J = 9.5 Hz, 2H); ^{13}C NMR (125 MHz, $CDCl_3$) δ 185.4, 147.1, 136.1, 133.9, 129.6, 127.2, 125.8, 48.6, 25.9; HRMS (APCI) *m/z* calcd for $C_{11}H_{12}NO_3S$ $[M+H]^+$ 238.0538, found 238.0552.

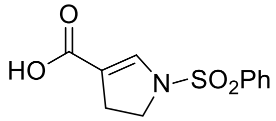

1-(Phenylsulfonyl)-4,5-dihydro-1*H*-pyrrole-3-carboxylic acid (21): $NaClO_2$ (0.97 g, 8.60 mmol) was added in one portion to a mixture of aldehyde **S3** (3.00 g, 12.6 mmol), 2-methyl-2-butene (25.3 mL, 2M in THF), and NaH_2PO_4 (7.59 g, 63.22 mmol) in 1,4-dioxane/H_2O (3:1; 80 mL). The reaction mixture was stirred for 1 h at room temperature. A saturated $NaHCO_3$ solution (50 mL) was added with precaution to the solution and the final mixture was stirred vigorously

for 30 min. The mixture was concentrated and the residue was dissolved in EtOAc. The organic mixture was washed with 10% HCl and H_2O, then evaporated under vacuum. The residue was purified by flash chromatography (50% EtOAc in hexanes) to give the title compound (3.02 g, 95%) as a white crystalline solid: mp 145–147 °C; ^{1}H NMR (400 MHz, $CDCl_3$) δ 7.82 (d, *J* = 8.0 Hz, 2H), 7.67 (t, *J* = 7.6 Hz, 1H), 7.58 (t, *J* = 7.6 Hz, 2H), 7.46 (s, 1H), 3.72 (t, *J* = 9.6 Hz, 2H), 2.79 (t, *J* = 9.2 Hz, 2H); ^{13}C NMR (100 MHz, $CDCl_3$) δ 169.2, 142.7, 136.2, 133.8, 129.5, 127.4, 113.5, 48.7, 27.8; HRMS (APCI) *m/z* calcd for $C_{11}H_{12}NO_4S$ $[M+H]^+$ 254.0487, found 254.0510.

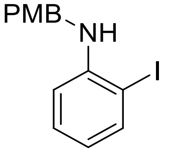

2-Iodo-*N*-(4-methoxylbenzyl)aniline (S4): $NaBH(OAc)_3$ (9.67 g, 45.6 mmol) was added to a solution of *p*-anisaldehyde (1.53 mL, 12.6 mmol), 2-iodoaniline (2.5 g, 11.4 mmol) and AcOH (0.11 mL, 0.11 mmol) in 1,2-DCE (60 mL). The mixture was stirred 24 h, then saturated aqueous $KHCO_3$ (30 mL) was slowly added while stirring vigorously. The aqueous layer was extracted with EtOAc, the combined organic layer was washed with brine, dried over $MgSO_4$, and volatile compounds were removed under vacuum. The residue was subjected to flash chromatography (20% EtOAc in hexanes) to give the title compound (3.53 g, 91%). ^{1}H NMR (400 MHz, $CDCl_3$) δ 7.68 (dd, *J* = 8.0 Hz, 1.2. Hz, 1H), 7.29 (d, *J* = 8.8 Hz, 2H), 7.17 (td, *J* = 8.0 Hz, 1.2 Hz, 1H), 6.90 (d, *J* = 8.8 Hz, 2H), 6.56 (dd, *J* = 8.4 Hz, 1.2 Hz, 1H), 6.45 (td, *J* = 8.0 Hz, 1.2 Hz, 1H), 4.54 (s, 1H), 4.33 (s, 2H), 3.81 (s, 3H); ^{13}C NMR (100 MHz, $CDCl_3$) δ 158.9, 147.1, 139.0, 130.5, 129.4, 128.8, 118.7, 114.1, 110.1, 85.3, 55.3, 47.8; HRMS (APCI) *m/z* calcd for $C_{14}H_{13}INO$ $[M–H]^+$ 338.0084, found 338.0070.

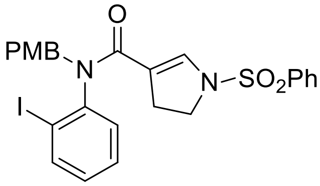

***N*-(2-Iodophenyl)-*N*-(4-methoxybenzyl)-1-(phenylsulfonyl)-4,5-dihydro-1*H*-pyrrole-3-carboxamide (22):** Ghosez reagent (0.15 mL, 1.25 mmol) was added to a suspension of carboxylic acid **21** (317 mg, 1.25 mmol) in toluene (12.5 mL), the mixture was stirred for 1 h. NaHMDS (0.93 mL, 1M in THF) was added to a solution of aniline **S4** (315 mg, 0.93 mmol) in THF (9 mL) at –78 °C. The mixture was stirred 30 minutes then cannulated into the stirring acid chloride solution. The mixture was stirred to rt for 3 h. The reaction was quenched with aqueous $KHCO_3$, the aqueous layer was extracted with EtOAc. The combined organic layer was washed with brine, dried over $MgSO_4$, volatile compounds were removed under vacuum. The residue was subjected to flash chromatography (20% EtOAc in hexanes) to give the title compound (320 mg, 60%) as a colorless solid: mp 53–56 °C. ^{1}H NMR (500 MHz, $CDCl_3$) δ 7.94 (dd, *J* = 8.0 Hz, 1.5 Hz, 1H), 7.60–7.57 (m, 1H), 7.49–7.48 (m, 4H), 7.28 (td, *J* = 8.0 Hz, 1.5 Hz, 1H), 7.18–7.09 (m, 2H), 6.79–6.76 (m, 3H), 5.83 (s, 1H), 5.49 (d, *J* = 14.0 Hz, 1H), 4.02 (d, *J* = 14.0 Hz, 1H), 3.75 (s, 3H), 3.45–3.41 (m, 1H), 3.29–3.29 (m, 1H), 2.65 (m, 1H), 2.55 (m, 1H); ^{13}C NMR (125 MHz, $CDCl_3$) δ 164.0, 159.0, 144.1, 140.2, 136.7, 135.7, 133.2, 131.1, 130.8, 128.0, 129.3, 129.2, 128.6, 118.1, 113.6, 101.0, 55.1, 52.0, 47.0, 30.8; HRMS (ESI) *m/z* calcd for $C_{25}H_{25}IN_2O_4S$ $[M+H]^+$ 575.2502, found 575.0508.

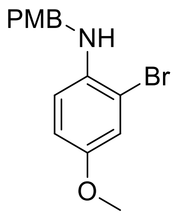

2-Bromo-4-methoxy-*N*-(4-methoxybenzyl)aniline (S5): $NaBH(OAc)_3$ (5.10 g, 24.1 mmol) was added to a solution of *p*-anisaldehyde (0.41 mL, 6.63 mmol), 2-bromo-4-methoxyaniline[4] (1.5 g, 6.02 mmol) and AcOH (0.11 mL, 1.91 mmol) in 1,2-DCE (30 mL). The mixture was stirred 24 h, then saturated aqueous $KHCO_3$ (15 mL) was slowly added while stirring vigorously. The aqueous layer was extracted with EtOAc, the combined organic layer was washed with brine, dried over $MgSO_4$, and volatile compounds were removed under vacuum. The residue was subjected to flash chromatography (20% EtOAc in hexanes) to give the title compound (2.14 g, 91%) as a white solid: mp 63–64 °C. ^{1}H NMR (400 MHz, $CDCl_3$) δ 7.29 (d, *J* = 6.8 Hz, 2H), 7.08 (d, *J* = 2.4 Hz, 1H), 6.89 (d, *J* = 6.8 Hz 2H), 6.77 (dd, *J* = 7.2 Hz, 2.4 Hz, 1H), 6.58 (d, *J* = 7.2 Hz, 1H), 4.33 (s, 1H), 4.28 (s, 2H), 3.81 (s, 3H), 3.73 (s, 3H).

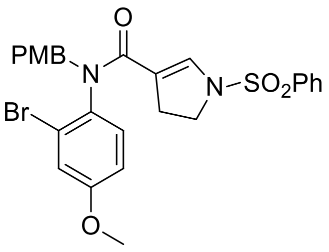

***N*-(2-Bromo-4-methoxyphenyl)-*N*-(4-methoxybenzyl)-1-(phenylsulfonyl)-4,5-dihydro-1*H*-pyrrole-3-carboxamide (23):** Ghosez reagent (75 µL, 0.57 mmol) was added to a suspension of carboxylic acid **21** (144 mg, 0.57 mmol) in toluene (5.7 mL), the mixture was stirred for 1 h. NaHMDS (0.38 mL, 1M in THF) was added to a solution of protected aniline **S5** (140 mg, 0.38 mmol) at –78 °C. The mixture was stirred 30 minutes, then cannulated into the stirring acid

chloride solution, the mixture was stirred to r.t. for 3 h. The reaction was quenched with $KHCO_3$, and the aqueous layer was extracted with EtOAc. The combined organic layer was washed with brine, dried over $MgSO_4$, volatile compounds were removed under vacuum. Flash chromatography (20% EtOAc in hexanes) gave the title compound (158 mg, 69%). ^{1}H NMR (500 MHz, $CDCl_3$) δ 7.61 (tt, J = 7.0 Hz, 1.8 Hz, 1H) 7.54–7.48 (m, 4H) 7.19 (d, J = 3.0 Hz, 1H), 7.10 (d, J = 8.5 Hz, 2H), 6.77 (d, J = 8.5 Hz, 2 H), 6.74 (dd, J = 9.0 Hz, 3.0 Hz, 1H), 6.66 (d, J = 9.0 Hz, 1H), 6.02 (s, 1H), 5.47 (d, J = 14.0 Hz, 1H), 4.02 (d, J = 14.0 Hz, 1H), 3.84 (s, 3H), 3.78 (s, 3H), 3.38 (ddd, J = 50.0 Hz, 10.0 Hz, 7.5 Hz, 2H), 2.67–2.48 (m, 2H); ^{13}C NMR (125 MHz, $CDCl_3$) δ 164.7, 159.8, 159.0, 136.7, 135.9, 133.6, 133.3, 132.0, 130.8, 129.2, 129.0, 127.3, 124.6, 119.0, 118.2, 113.8, 113.7, 55.9, 55.2, 47.3, 30.7; HRMS (ESI) *m/z* calcd for $C_{26}H_{25}BrNaN_2O_5S$ $[M+Na]^+$ 579.0565, found 579.0604.

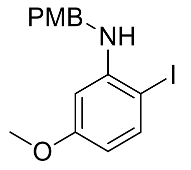

2-Iodo-5-methoxy-*N*-(4-methoxybenzyl)aniline (S6): $NaBH(OAc)_3$ (5.10 g, 24.1 mmol) was added to a solution of *p*-anisaldehyde (0.41 mL, 6.63 mmol), 2-iodo-5-methoxyaniline[5] (1.5 g, 6.02 mmol) and AcOH (0.11 mL, 0.11 mmol) in 1,2-DCE (30 mL). The mixture was stirred 24 h, then saturated aq. $KHCO_3$ (15 mL) was slowly added while stirring vigorously. The aqueous layer was extracted with EtOAc, the combined organic layer was washed with brine, dried over $MgSO_4$, and volatile compounds were removed under vacuum. The residue was subjected to flash chromatography (20% EtOAc in hexanes) to give the title compound (1.80 g, 81%). ^{1}H NMR (400 MHz, $CDCl_3$) δ 7.50 (d, J = 8.6 Hz, 1H), 7.26 (d, J= 8.6 Hz, 2H), 6.87 (d, J= 8.6 Hz, 2H), 6.14 (d, J = 2.8 Hz, 1H), 6.07 (dd, J = 8.6 Hz, 2.8 Hz, 1H), 4.49 (s, 1H), 4.27 (s, 1H), 4.26

(s, 1H), 3.78 (s, 3H), 3.69 (s, 3H); ^{13}C NMR (100 MHz, $CDCl_3$) δ 161.2, 158.8, 147.9, 138.9, 130.3, 128.5, 114.0, 104.0, 97.9, 74.5, 55.2, 47.8.

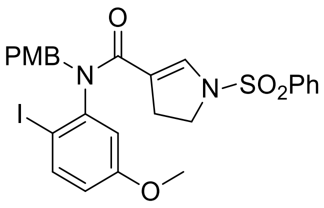

***N*-(2-Iodo-5-methoxyphenyl)-*N*-(4-methoxybenzyl)-1-(phenylsulfonyl)-4,5-dihydro-1*H*-pyrrole-3-carboxamide (24):** Ghosez reagent (0.12 mL, 0.93 mmol) was added to a suspension of carboxylic acid **21** (236 mg, 0.93 mmol) in toluene (7 mL), the mixture was stirred for 1 h. NaHMDS (0.72 mL, 1M in THF) was added to a solution of aniline **S6** (265 mg, 0.72 mmol) in THF (7 mL) at –78 °C. The mixture was stirred 30 minutes, then added by cannula into the stirring acid chloride solution, the mixture was warmed to room temperature and stirred for 3 h. The reaction was quenched with $KHCO_3$, and the aqueous layer was extracted with EtOAc. The combined organic layer was washed with brine and dried over $MgSO_4$; volatiles were removed under vacuum. The residue was purified by flash chromatography (20% EtOAc in hexanes) to give the title compound (235 mg, 54%) as a colorless oil: ^{1}H NMR (500 MHz, $CDCl_3$) δ 7.79 (d, J = 9.0 Hz, 1H), 7.61 (tt, J = 6.5 Hz, 2.0 Hz, 1H), 7.53–7.50 (m, 3H), 7.13 (d, J = 8.5 Hz, 2H), 6.78 (d, J = 8.5 Hz, 2H), 6.74 (dd, J = 9.0 Hz, 3.0 Hz, 1H), 6.29 (d, J = 3.0 Hz, 1H), 5.92 (s, 1H), 5.52 (d, J = 14.5 Hz, 1H), 3.98 (d, J = 14.5 Hz, 1H), 3.77 (s, 3H), 3.61 (s, 3H), 3.42 (td, J = 10.0 Hz, 7.5 Hz, 1H), 3.34 (td, J = 10.0 Hz, 7.5 Hz, 1H), 2.72–2.58 (m, 2H); ^{13}C NMR (125 MHz, $CDCl_3$) δ 163.9, 160.5, 159.1, 144.9, 140.2, 136.9, 135.3, 133.3, 130.9, 129.3, 128.9, 127.3, 118.1, 117.0, 116.6, 113.7, 89.0, 55.6, 55.2, 52.0, 57.1, 30.9; HRMS (ESI) *m/z* calcd for $C_{26}H_{26}IN_2O_5S$ $[M+H]^+$ 605.0607, found 605.0616.

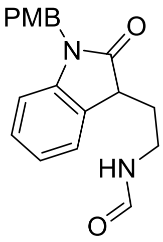

***N*-(2-(1-(4-Methoxybenzyl)-2-oxoindolin-3-yl)ethyl)formamide (25):** A solution of Bu_3SnH (0.24 mL, 0.83 mmol) and AIBN (14 mg, 0.014 mmol) in degassed benzene (4 mL) were added via syringe pump to a refluxing solution of iodoaniline **22** (98 mg, 0.17 mmol) in degassed benzene (8 mL) to afford the title compound (34 mg, 65%) as a colorless solid: mp >220 °C. ^1H NMR (400 MHz, $CDCl_3$) δ 8.14 (s, 1H), 7.28 (d, *J* = 7.6 Hz, 1H), 7.23 (d, *J* = 7.2 Hz, 2H), 7.20 (t, *J* = 7.6 Hz, 1H), 7.05 (t, *J* = 7.6 Hz, 1H), 6.84 (d, *J* = 7.6 Hz, 2H), 6.78 (d, *J* = 7.6 Hz, 1H), 6.52 (s, 1H), 4.84 (s, 2H), 3.77 (s, 3H), 3.59–3.54 (m, 3H), 2.32 (dq, *J* = 14.0 Hz, 6.0 Hz, 1H), 2.03 (hex, *J* = 7.2 Hz, 1H); ^{13}C NMR (100 MHz, $CDCl_3$) δ 178.0, 161.3, 159.1, 143.0, 128.7, 128.6, 128.4, 128.2, 127.7, 123.9, 122.9, 114.2, 109.2, 55.3, 44.1, 43.3, 36.0, 30.1; HRMS (TOF ES) *m/z* calcd for $C_{19}H_{21}N_2O_3$ $[M+H]^+$ 325.1552, found 325.1562.

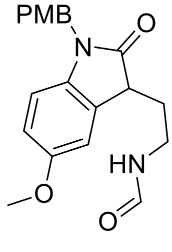

***N*-(2-(5-Methoxy-1-(4-methoxybenzyl)-2-oxoindolin-3-yl)ethyl)formamide (26):** A solution of Bu_3SnH (0.24 mL, 0.83 mmol) and AIBN (14 mg, 0.014 mmol) in degassed benzene (4 mL) were added via syringe pump to a refluxing solution of bromoaniline **23** (100 mg, 0.17 mmol) in degassed benzene (8 mL). After 2 h, the mixture was cooled to r.t. and the volatile compounds were removed under vacuum. Flash chromatography (0–5 % MeOH in CH_2Cl_2) gives the title

compound (25 mg, 42%) as a white solid, mp >220 °C. ^{1}H NMR (500 MHz, $CDCl_3$) δ 8.15 (s, 1H), 7.21 (dt, J = 10.0 Hz, 2.5 Hz, 2H), 6.91 (dd, J = 2.5 Hz, 0.9 Hz, 1H), 6.84 (dt, J = 10.0 Hz, 2.5 Hz, 2H), 6.71 (dd, J = 8.0 Hz, 2.5 Hz, 1H), 6.66 (d, J = 8.5 Hz, 1H), 4.82 (s, 2H), 3.77 (s, 3H), 3.76 (S, 3H), 3.58–3.53 (m, 2H), 2.31 (dq, J = 15.0 Hz, 6.0 Hz, 1H), 2.06–1.98 (m, 1H); ^{13}C NMR (125 MHz, $CDCl_3$) δ 177.6, 161.3, 159.1, 156.2, 136.4, 129.7, 128.6, 127.7, 114.2, 112.6, 111.2, 109.6, 55.7, 55.2, 44.4, 43.3, 35.9, 30.1; HRMS (ESI) *m/z* calcd for $C_{20}H_{21}N_2O_3$ $[M+H]^+$ 337.1552, found 337.1558.

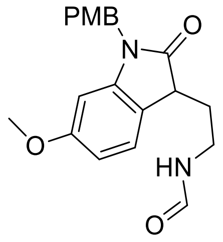

***N*-(2-(6-Methoxy-1-(4-methoxybenzyl)-2-oxoindolin-3-yl)ethyl)formamide (27):** A solution of Bu_3SnH (0.06 mL, 0.40 mmol) and AIBN (3 mg, 0.003 mmol) in degassed benzene (1 mL) were added via syringe pump to a refluxing solution of iodoanilide **24** (20 mg, 0.03 mmol) in degassed benzene (1.5 mL). After heating an additional 2 h, the mixture was cooled to room temperature and volatile compounds were removed under vacuum. The residue was purified by flash chromatography (5% MeOH in CH_2Cl_2) to give the title compound (6 mg, 54%) as a white solid: mp >220 °C. ^{1}H NMR (500 MHz, $CDCl_3$) δ 8.13 (s, 1H), 7.22 (dt, J = 10.0 Hz, 2.5 Hz, 2H), 7.17 (d, J = 8.5 Hz, 1H), 6.84 (dt, J = 10.0 Hz, 2.5 Hz, 2H), 6.54 (dd, J = 8.0 Hz, 2.5 Hz, 1H), 6.37 (d, J = 2.5 Hz, 1H), 4.81 (s, 2H), 3.77 (s, 3H), 3.75 (S, 3H), 3.56–3.49 (m, 2H), 2.26 (dq, J = 14.5 Hz, 6.0 Hz, 1H), 2.04–1.97 (m, 1H); ^{13}C NMR (125 MHz, $CDCl_3$) δ 178.7, 161.3, 160.1, 159.2, 144.2, 128.7, 127.7, 124.4, 120.2, 114.3, 106.4, 97.6, 55.5, 55.3, 43.5, 43.3, 36.0, 30.3; HRMS (ESI) *m/z* calcd for $C_{20}H_{21}N_2O_3$ $[M+H]^+$ 337.1552, found 337.1558.

References

1. Zi, W.; Wu, H.; Toste, D. F. *J. Am. Chem. Soc.* **2015**, *137*, 3225-3228.

2. Zhang, H.; Hay, E. B.; Geib, S. J.; Curran, D. P. *J. Am. Chem. Soc.* **2013,** *135*, 16610-16617.

3. Stevens, C. V.; Van Meenen, E.; Eeckhout, Y.; Vanderhoydonck, B.; Hooghe, W., *Chem. Commun.* **2005,** *38*, 4827-4829.

4. Kondo, Y.; Kojima, S.; Sakamoto, T. *J. Org. Chem.* **1997,** *62*, 6507-6511.

5. Roschangar, F.; Liu, J.; Estanove, E.; Dufour, M.; Rodríguez, S.; Farina, V.; Hickey, E.; Hossain, A.; Jones, P. J.; Lee, H.; Lu, B. Z.; Varsolona, R.; Schröder, J.; Beaulieu, P.; Gillard, J.; Senanayake, C. H. *Tetrahedron Lett.* **2008,** *49*, 363-366.

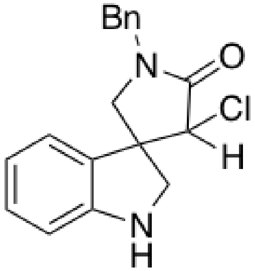
Bn
N
O
Cl
H
N
H

5b

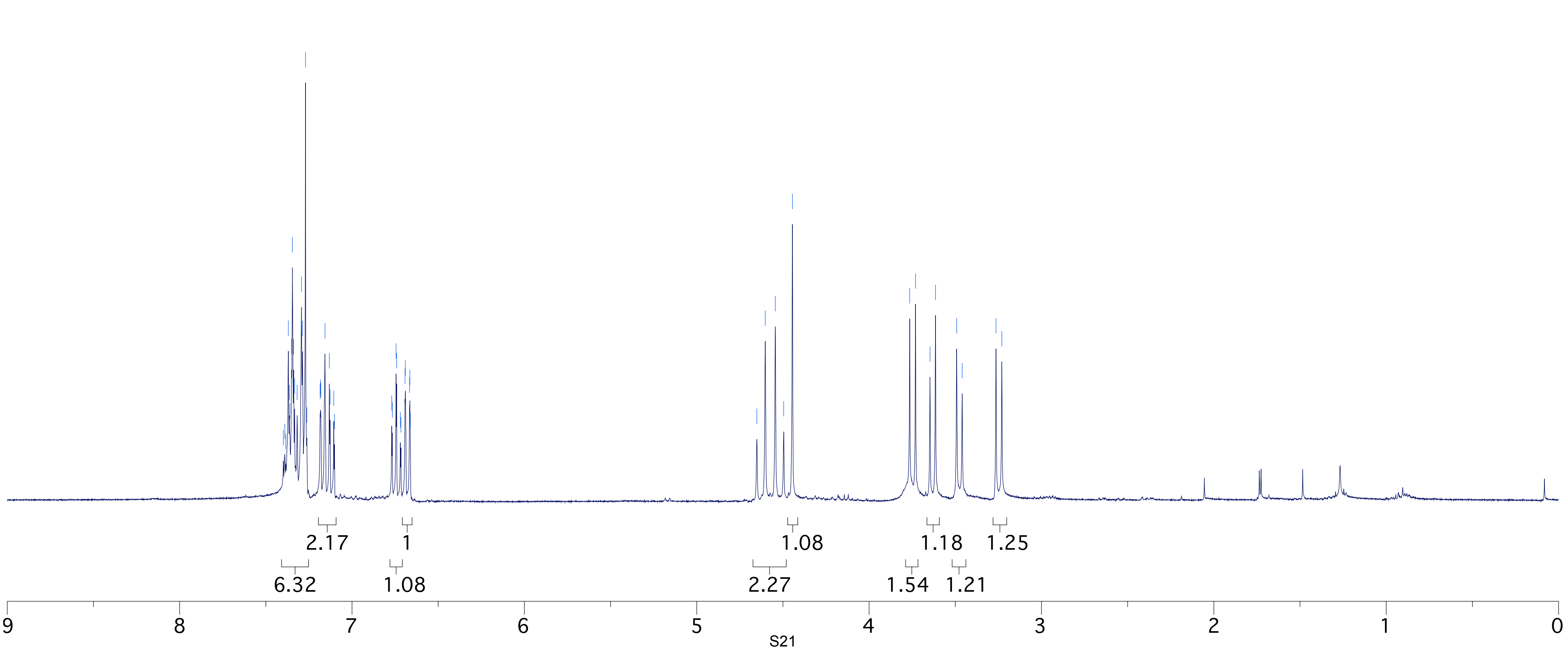
7.398
7.390
7.384
7.370
7.364
7.361
7.351
7.346
7.339
7.333
7.318
7.294
7.287
7.270
7.262
7.185
7.183
7.181
7.157
7.132
7.127
7.106
7.102
6.769
6.766
6.744
6.741
6.719
6.716
6.692
6.690
6.666
6.664
6.661
4.651
4.603
4.544
4.496
4.445
3.764
3.731
3.647
3.615
3.492
3.460
3.264
3.230
2.17
1
1.08
1.18
1.25
6.32
1.08
2.27
1.54
1.21
9
8
7
6
5
4
3
2
1
0

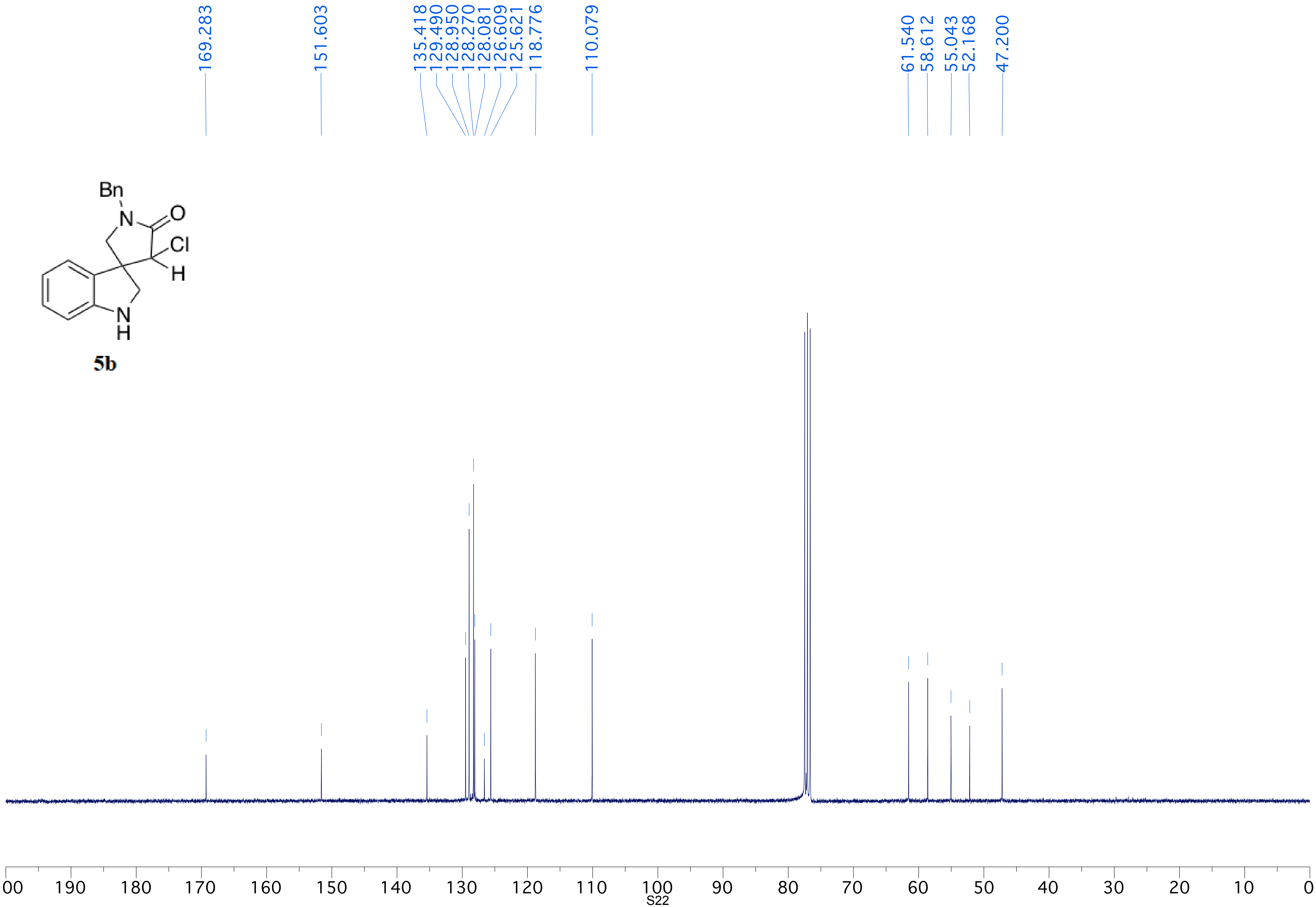
169.283
151.603
135.418
129.490
128.950
128.270
128.081
126.609
125.621
118.776
110.079
61.540
58.612
55.043
52.168
47.200
Bn
N
O
Cl
H
N
H
5b
00
190
180
170
160
150
140
130
120
110
100
90
80
70
60
50
40
30
20
10
0

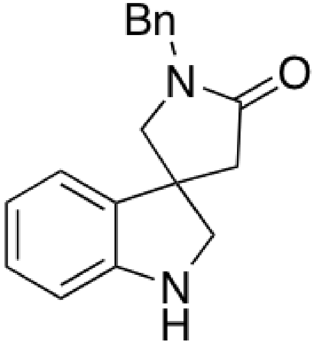

Bn
N
O
N
H

5c

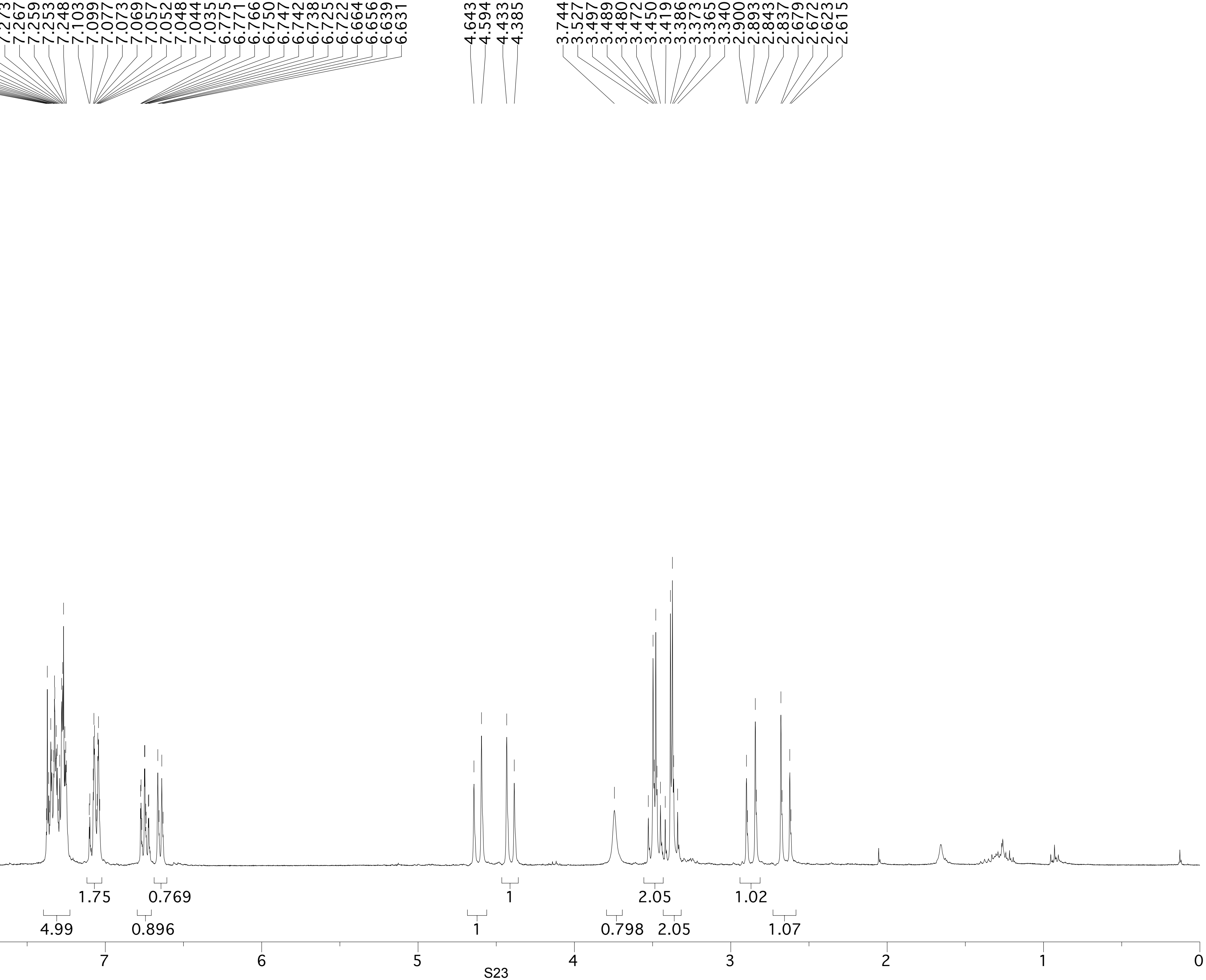

7.377
7.371
7.363
7.356
7.349
7.343
7.330
7.325
7.323
7.314
7.308
7.300
7.292
7.279
7.273
7.267
7.259
7.253
7.248
7.103
7.099
7.077
7.073
7.069
7.057
7.052
7.048
7.044
7.035
6.775
6.771
6.766
6.750
6.747
6.742
6.738
6.725
6.722
6.664
6.656
6.639
6.631
4.643
4.594
4.433
4.385
3.744
3.527
3.497
3.489
3.480
3.472
3.450
3.419
3.386
3.373
3.365
3.340
2.900
2.893
2.843
2.837
2.679
2.672
2.623
2.615
1.75
0.769
1
2.05
1.02
4.99
0.896
1
0.798
2.05
1.07
9
8
7
6
5
4
3
2
1
0

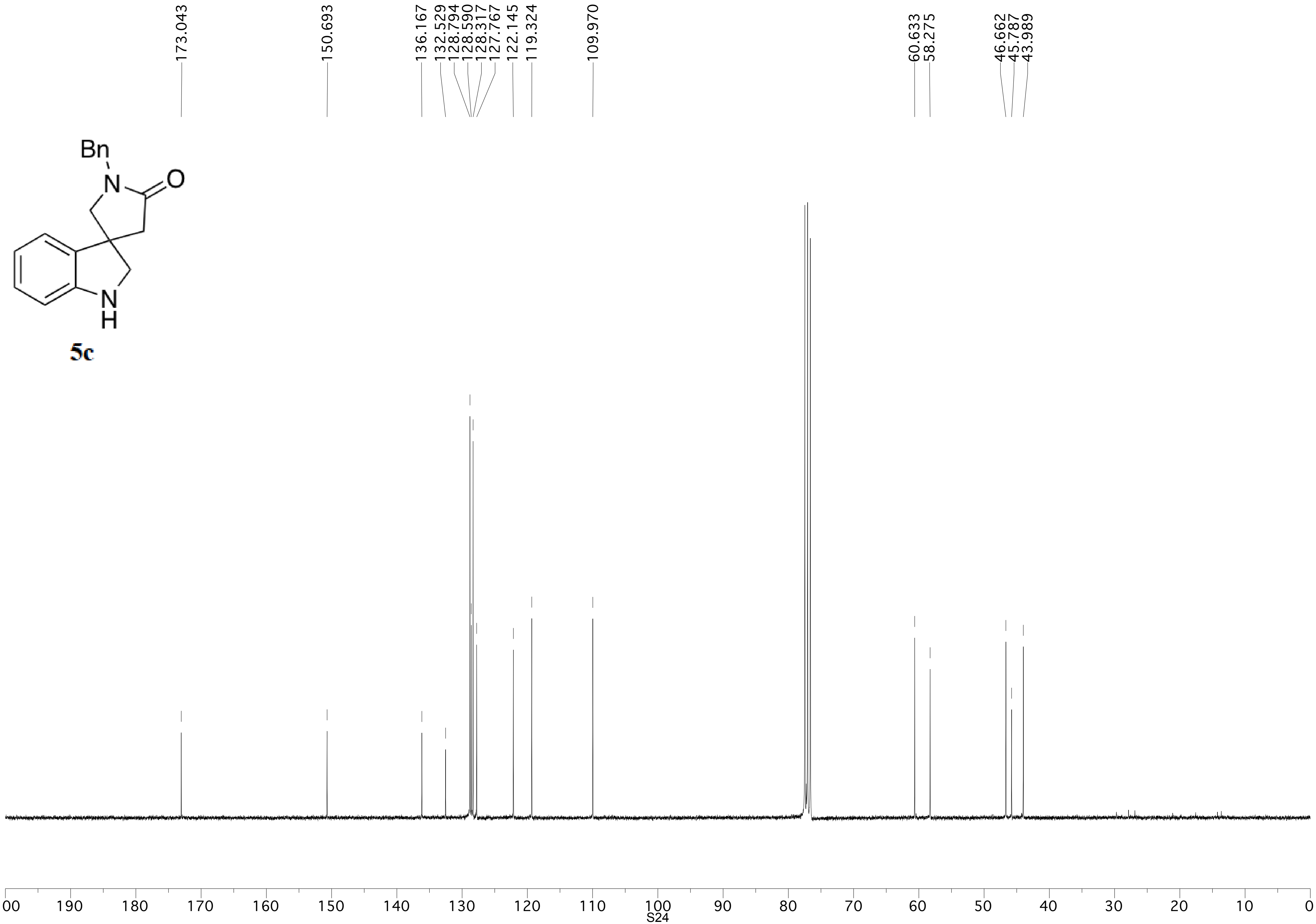
173.043
150.693
136.167
132.529
128.794
128.590
128.317
127.767
122.145
119.324
109.970
60.633
58.275
46.662
45.787
43.989
Bn
N
O
N
H
5c
00 190 180 170 160 150 140 130 120 110 100 90 80 70 60 50 40 30 20 10 0

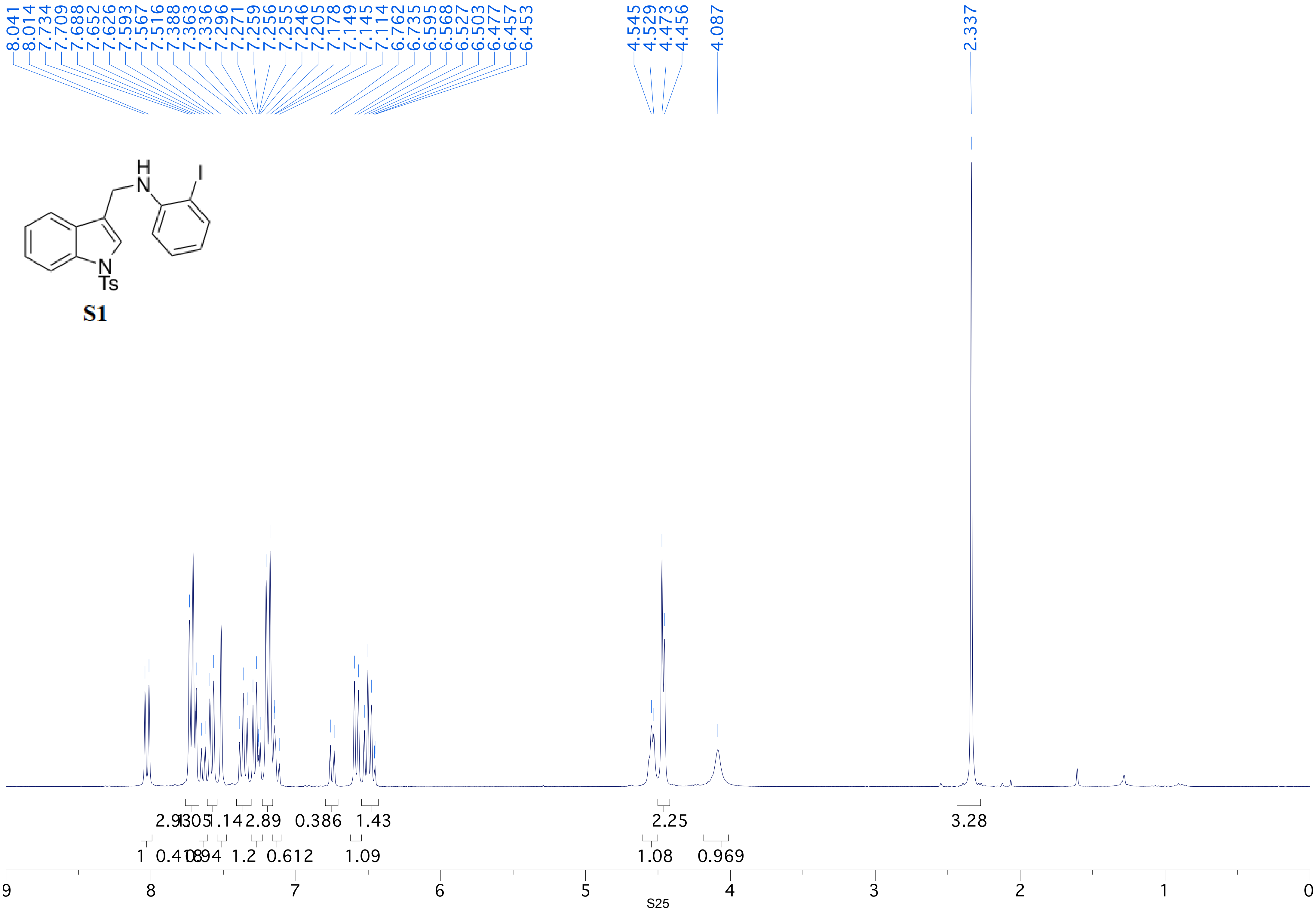
8.041
8.014
7.734
7.709
7.688
7.652
7.626
7.593
7.567
7.516
7.388
7.363
7.336
7.296
7.271
7.259
7.256
7.255
7.246
7.205
7.178
7.149
7.145
7.114
6.762
6.735
6.595
6.568
6.527
6.503
6.477
6.457
6.453
4.545
4.529
4.473
4.456
4.087
2.337
H
N
I
N
Ts
S1
2.91305
1.14
2.89
0.386
1.43
2.25
3.28
1
0.41094
1.2
0.612
1.09
1.08
0.969
9
8
7
6
5
4
3
2
1
0

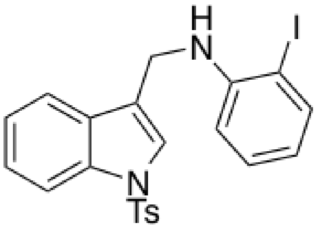
H
N
I
N
Ts

S1

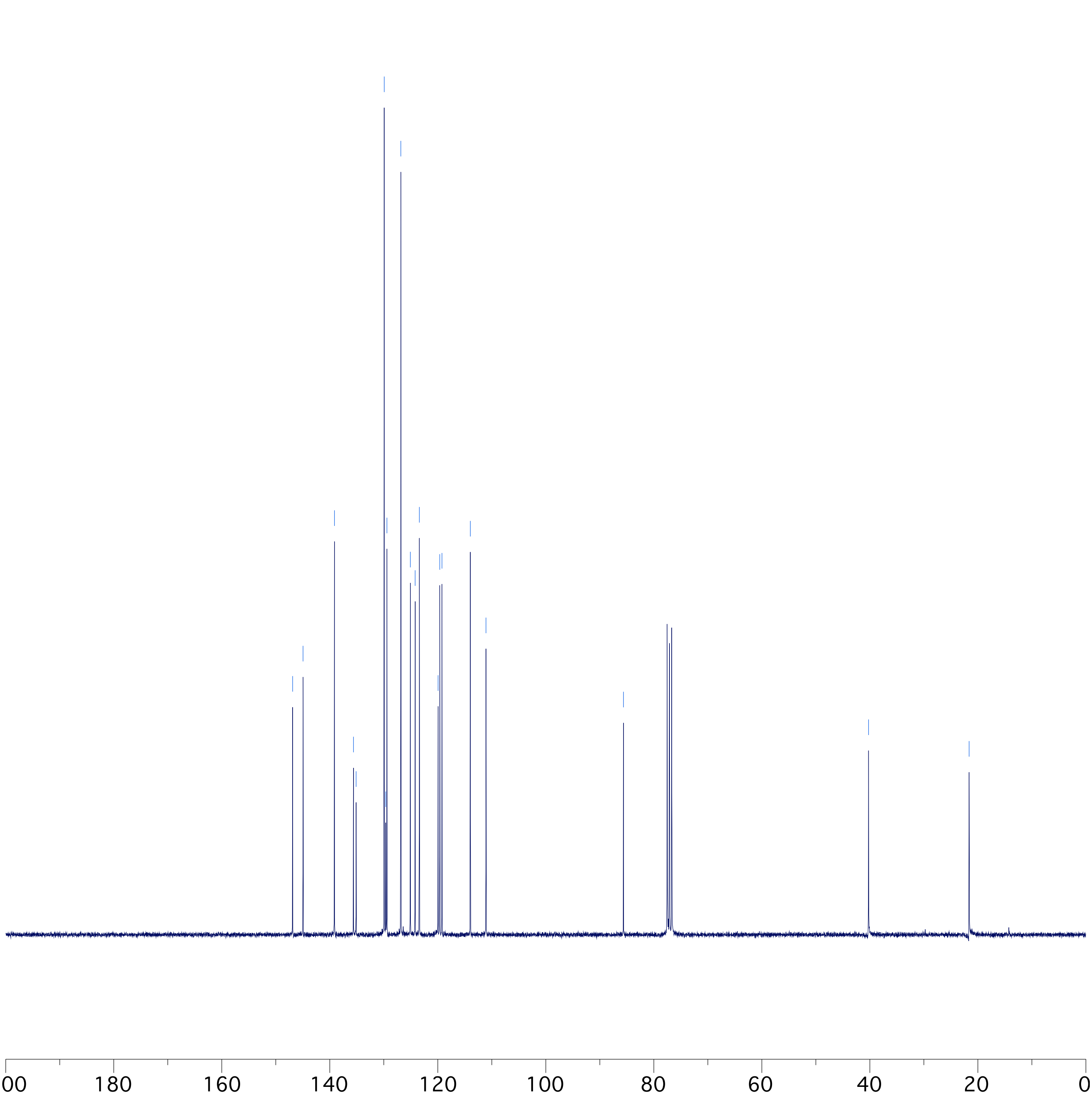
146.873
144.954
139.136
135.617
135.124
129.915
129.646
129.420
126.847
125.087
124.200
123.414
119.951
119.631
119.225
113.974
111.077
85.627
40.245
21.622
00
180
160
140
120
100
80
60
40
20
0

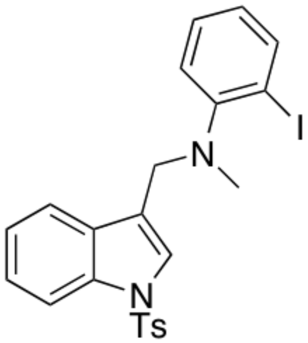
I
N
N
Ts

7

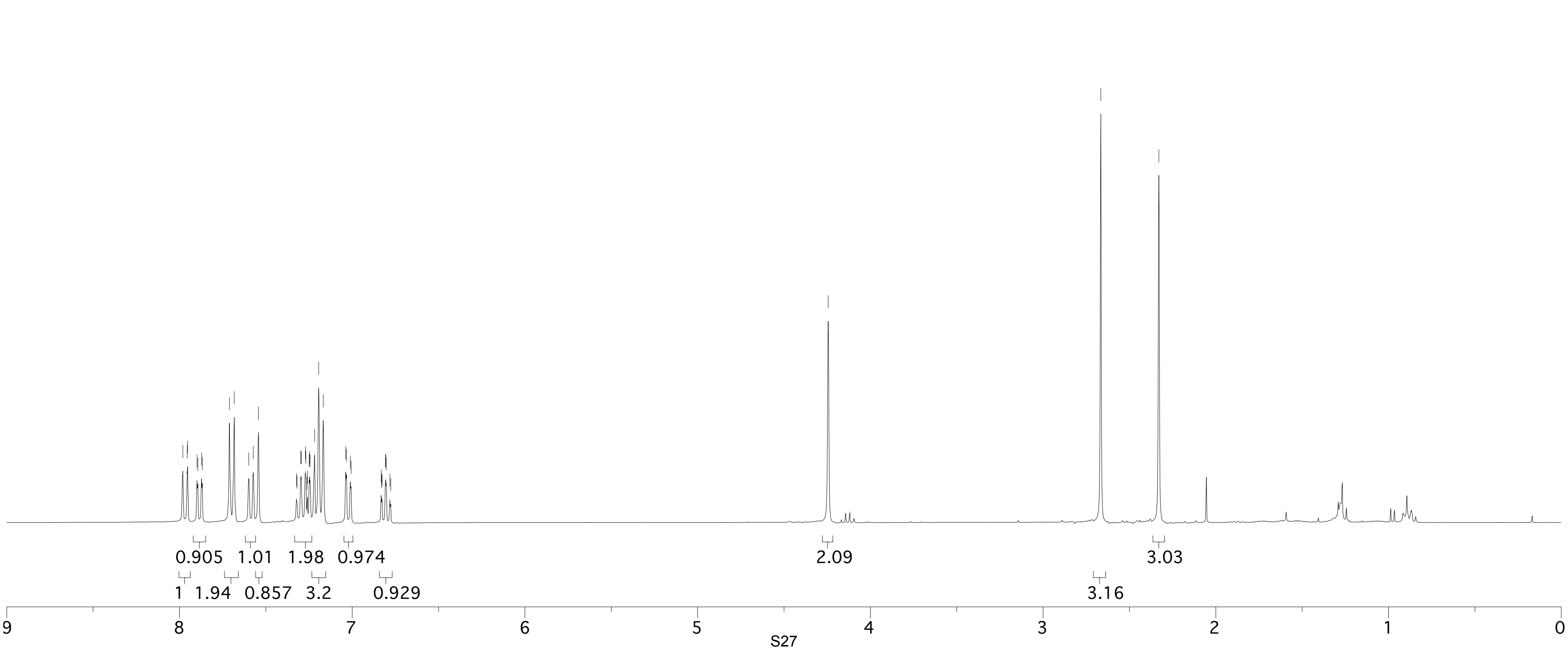
7.981
7.955
7.953
7.898
7.894
7.872
7.868
7.711
7.683
7.599
7.573
7.542
7.322
7.319
7.298
7.295
7.271
7.268
7.261
7.259
7.249
7.247
7.244
7.218
7.194
7.168
7.037
7.033
7.011
7.006
6.832
6.829
6.827
6.808
6.806
6.803
6.782
6.777
4.244
2.666
2.330
0.905
1.01
1.98
0.974
2.09
3.03
1
1.94
0.857
3.2
0.929
3.16
9
8
7
6
5
4
3
2
1
0

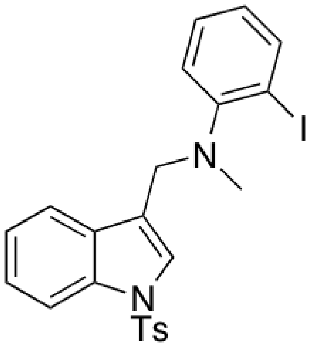
N
I
N
Ts

7

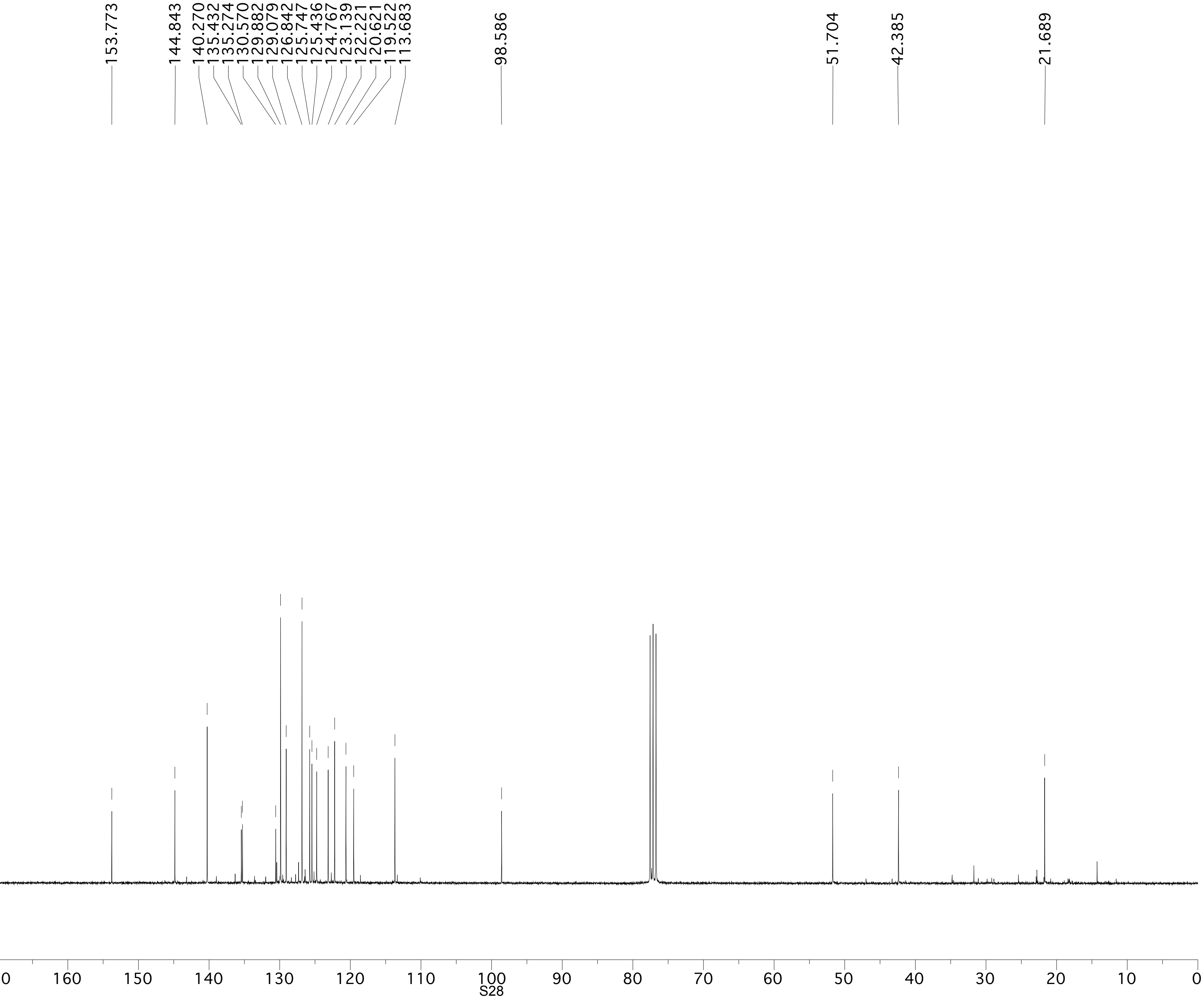
153.773
144.843
140.270
135.432
135.274
130.570
129.882
129.079
126.842
125.747
125.436
124.767
123.139
122.221
120.621
119.522
113.683
98.586
51.704
42.385
21.689
00 190 180 170 160 150 140 130 120 110 100 90 80 70 60 50 40 30 20 10 0

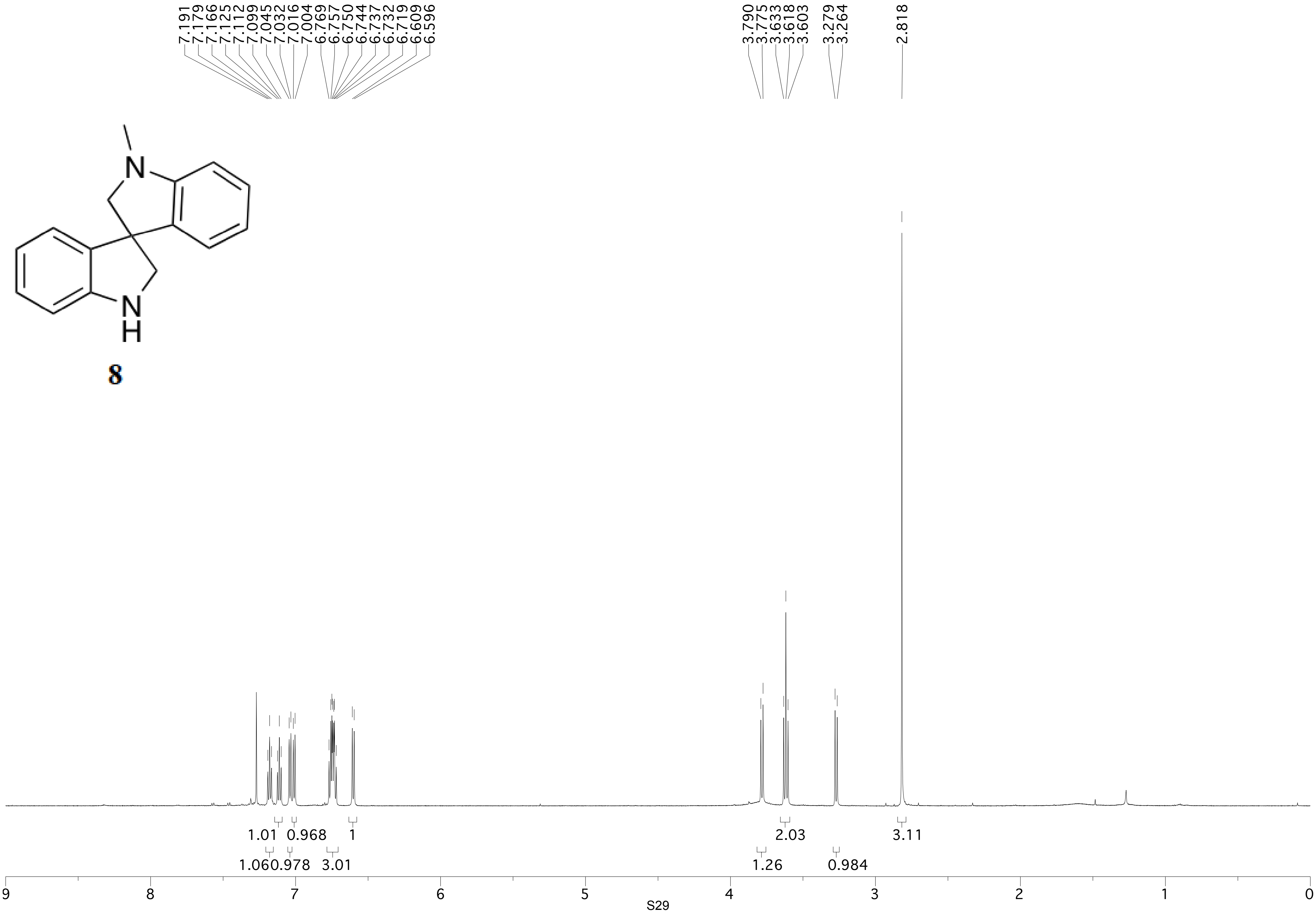
N
N
H
7.191
7.179
7.166
7.125
7.112
7.099
7.045
7.032
7.016
7.004
6.769
6.757
6.750
6.744
6.737
6.732
6.719
6.609
6.596
3.790
3.775
3.633
3.618
3.603
3.279
3.264
2.818
1.01
0.968
1
2.03
3.11
1.06
0.978
3.01
1.26
0.984
9
8
7
6
5
4
3
2
1
0

8

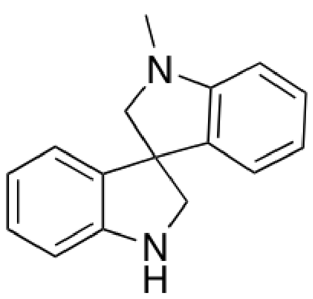
N
N
H

8

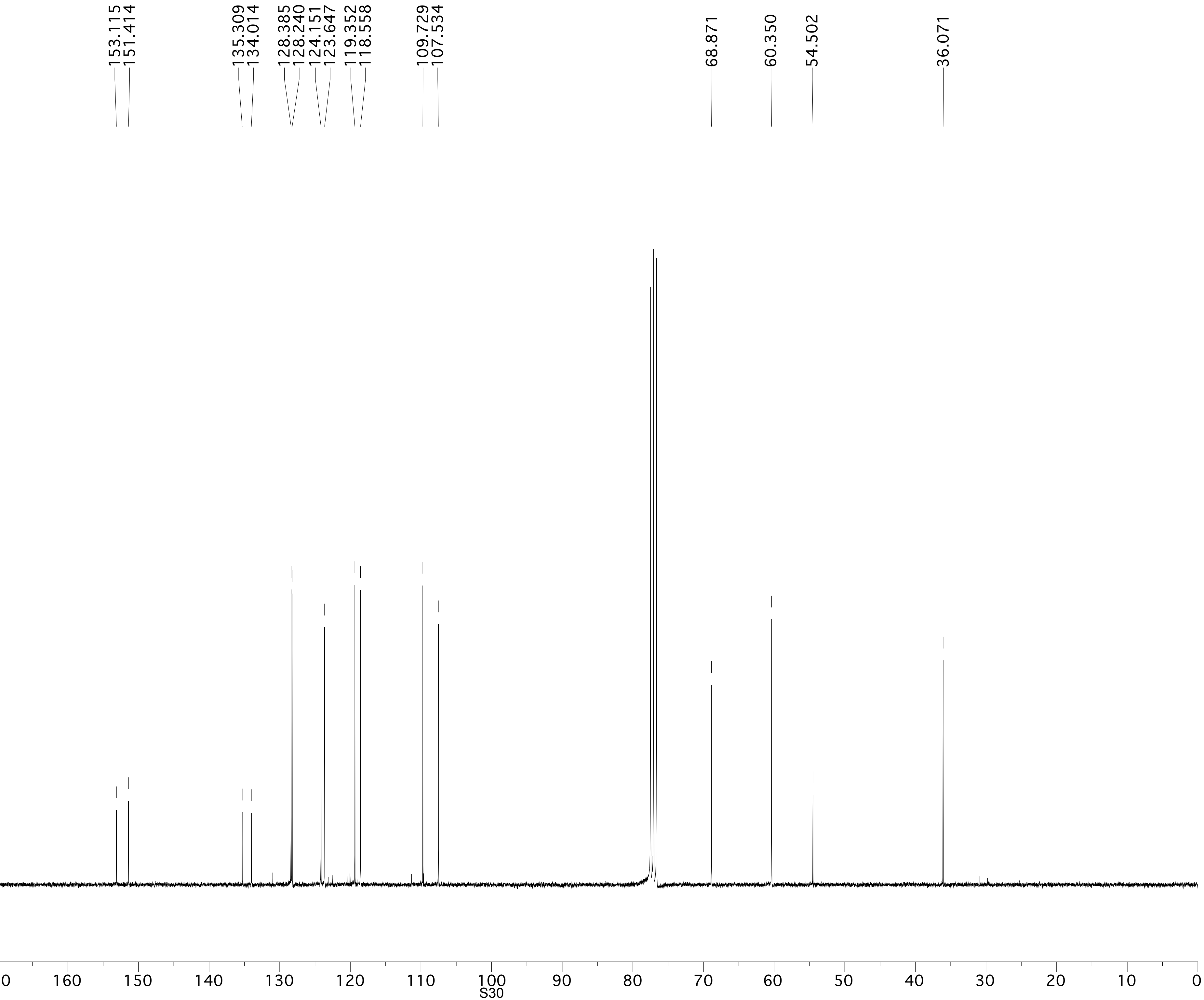
153.115
151.414
135.309
134.014
128.385
128.240
124.151
123.647
119.352
118.558
109.729
107.534
68.871
60.350
54.502
36.071
00 190 180 170 160 150 140 130 120 110 100 90 80 70 60 50 40 30 20 10 0

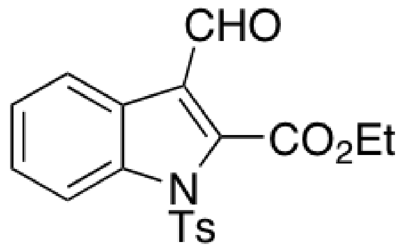

9

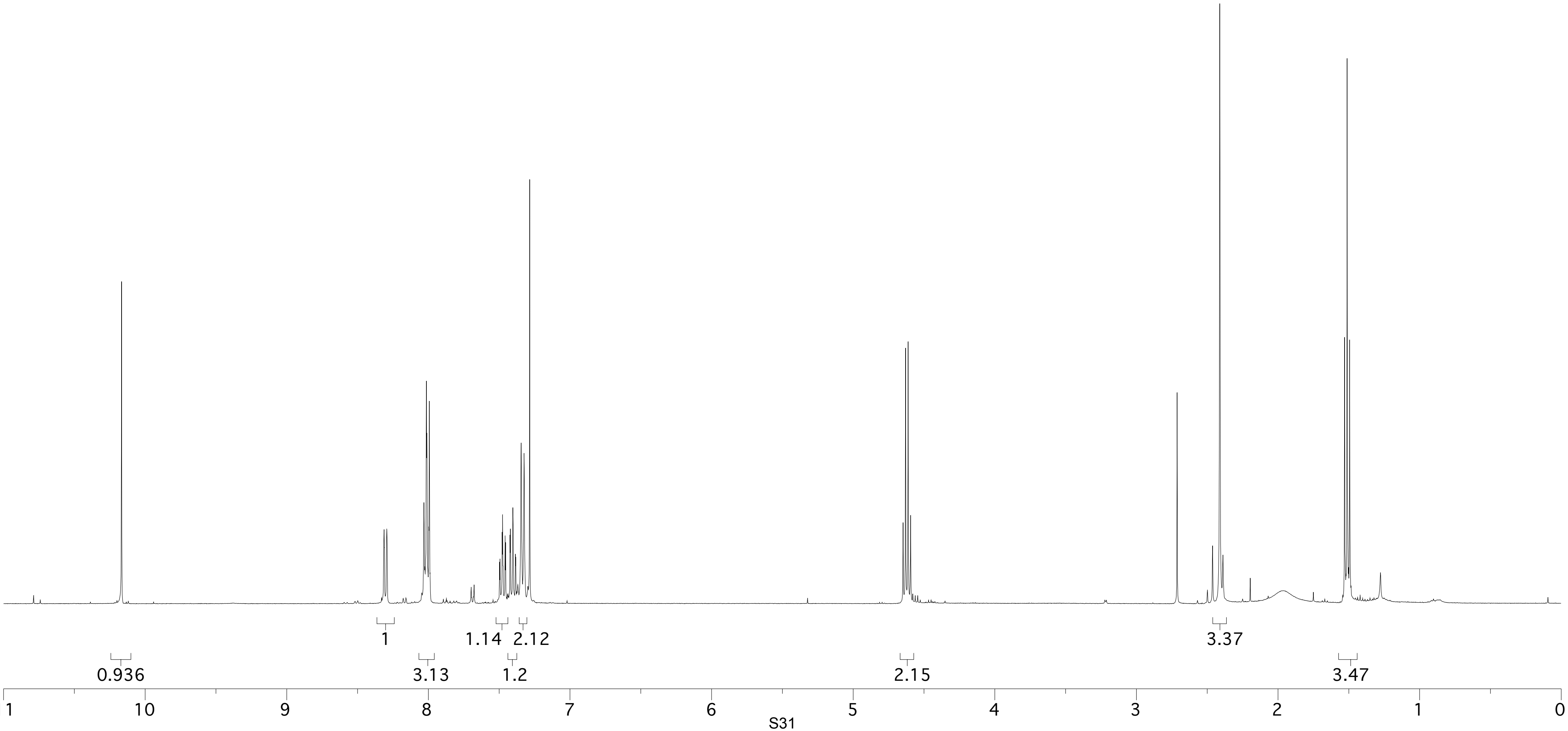

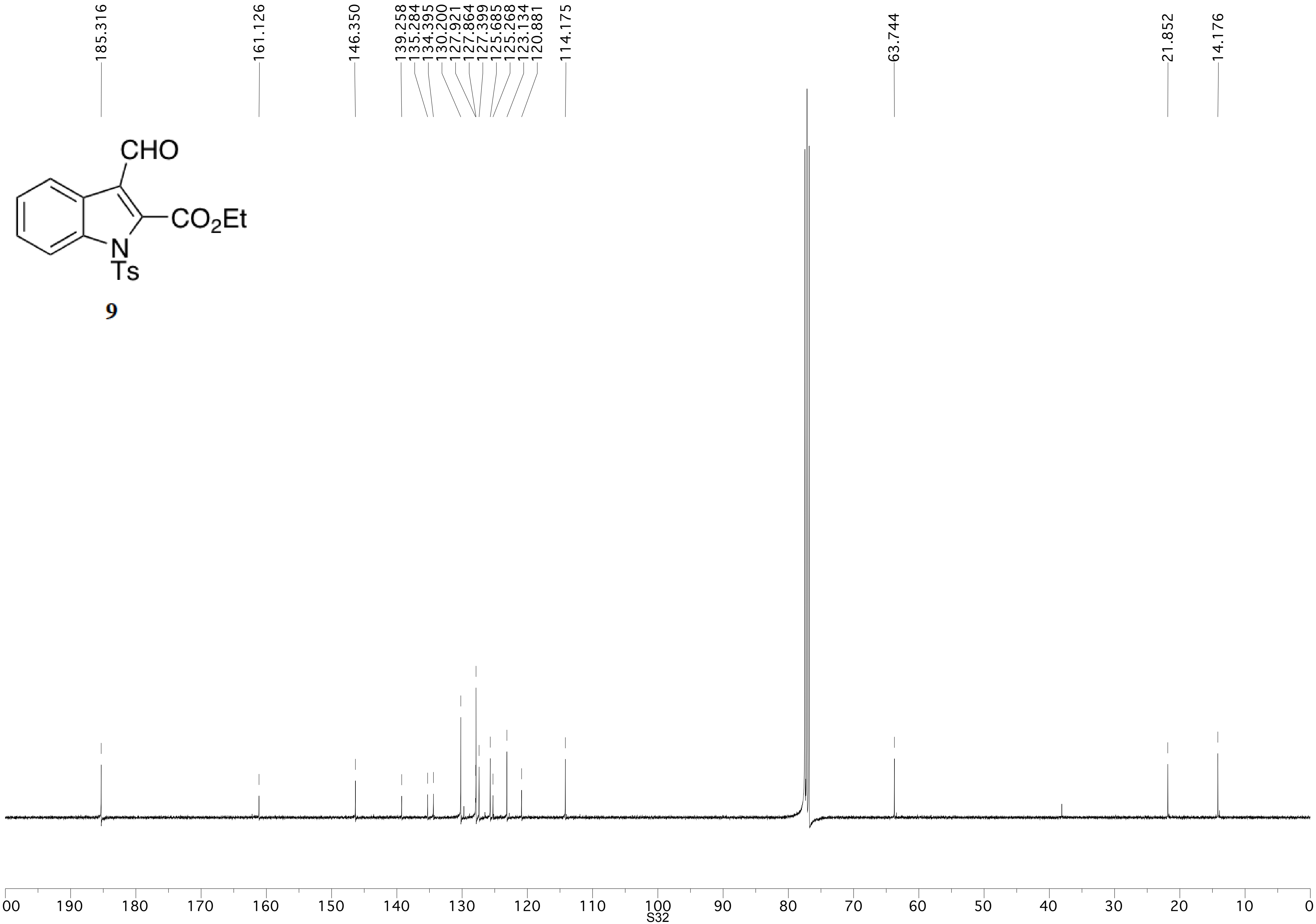
185.316
161.126
146.350
139.258
135.284
134.395
130.200
127.921
127.864
127.399
125.685
125.268
123.134
120.881
114.175
63.744
21.852
14.176
CHO
CO2Et
N
Ts
190
180
170
160
150
140
130
120
110
100
90
80
70
60
50
40
30
20
10
0

9

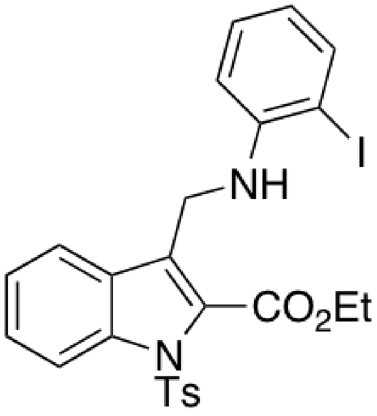
NH
I
CO2Et
N
Ts

S2

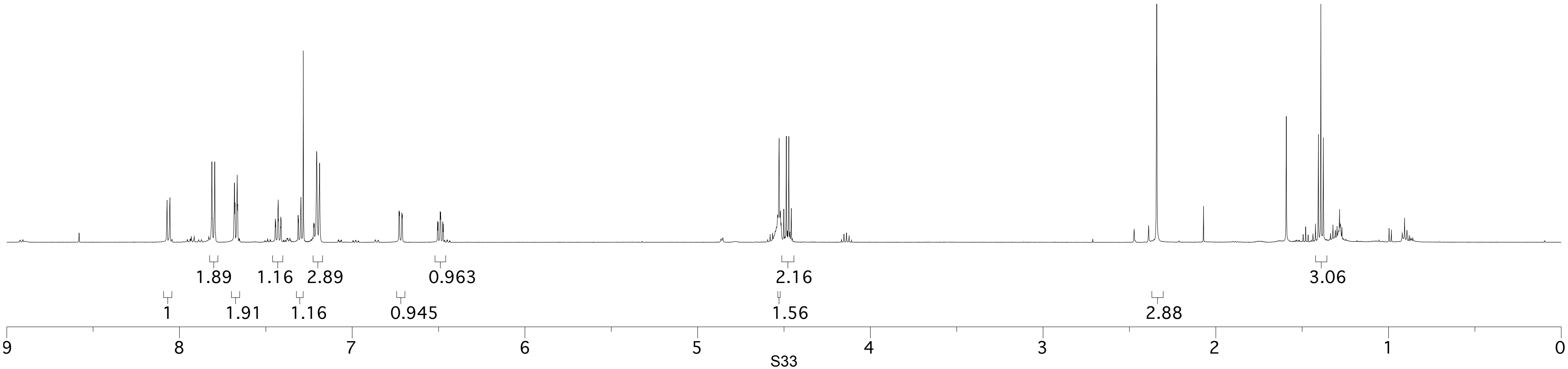
1.89
1.16
2.89
0.963
2.16
3.06
1
1.91
1.16
0.945
1.56
2.88
9
8
7
6
5
4
3
2
1
0

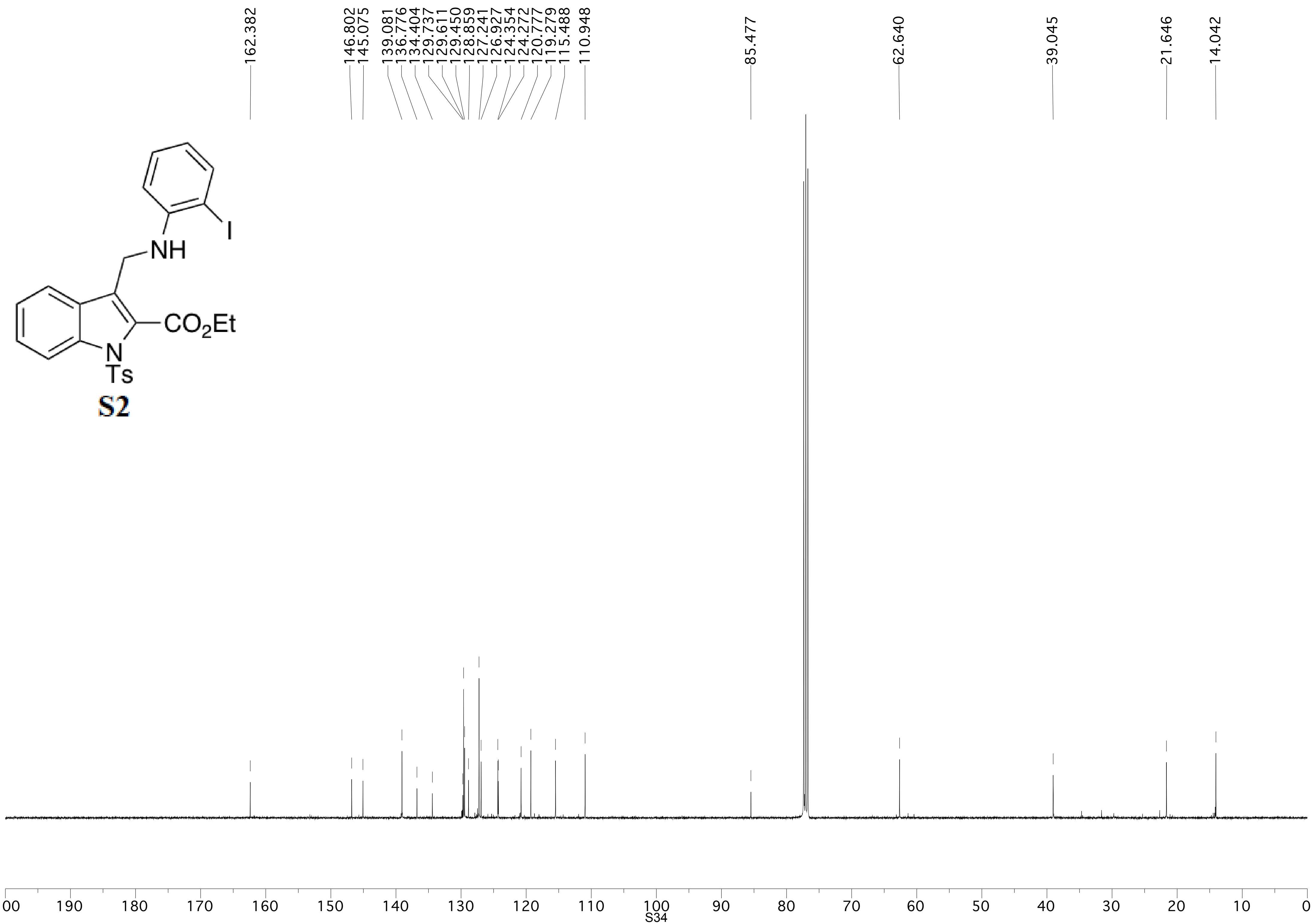
162.382
146.802
145.075
139.081
136.776
134.404
129.737
129.611
129.450
128.859
127.241
126.927
124.354
124.272
120.777
119.279
115.488
110.948
85.477
62.640
39.045
21.646
14.042
NH
I
CO2Et
N
Ts
S2
00
190
180
170
160
150
140
130
120
110
100
90
80
70
60
50
40
30
20
10
0

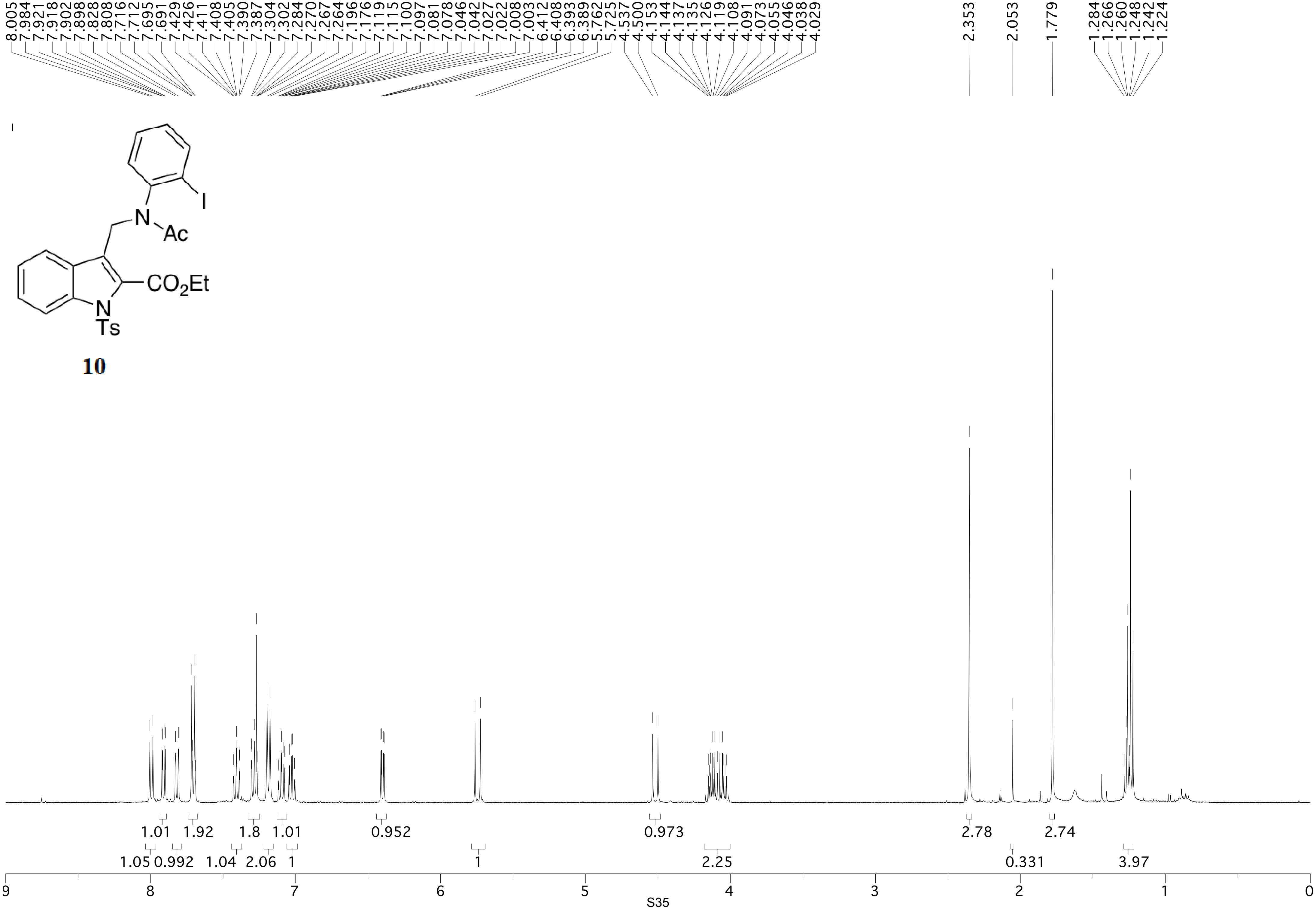
I
CO2Et
N
Ts
N
Ac
8.005 7.984 7.921 7.918 7.902 7.898 7.828 7.808 7.716 7.712 7.695 7.691 7.429 7.426 7.411 7.408 7.405 7.390 7.387 7.304 7.302 7.284 7.270 7.267 7.264 7.196 7.176 7.119 7.115 7.100 7.097 7.081 7.078 7.046 7.042 7.027 7.022 7.008 7.003 6.412 6.408 6.393 6.389 5.762 5.725 4.537 4.500 4.153 4.144 4.137 4.135 4.126 4.119 4.108 4.091 4.073 4.055 4.046 4.038 4.029
2.353
2.053
1.779
1.284 1.266 1.260 1.248 1.242 1.224
1.01 1.92 1.8 1.01 0.952 0.973 2.78 2.74
1.05 0.992 1.04 2.06 1 1 2.25 0.331 3.97
9 8 7 6 5 4 3 2 1 0

10

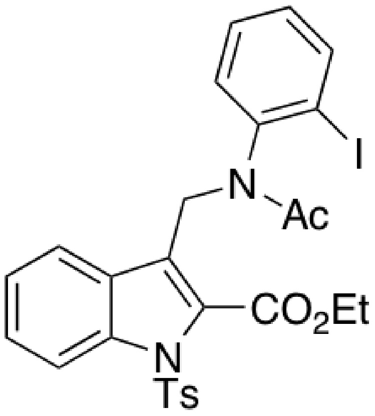
CO2Et
N
Ts
N
Ac
I

10

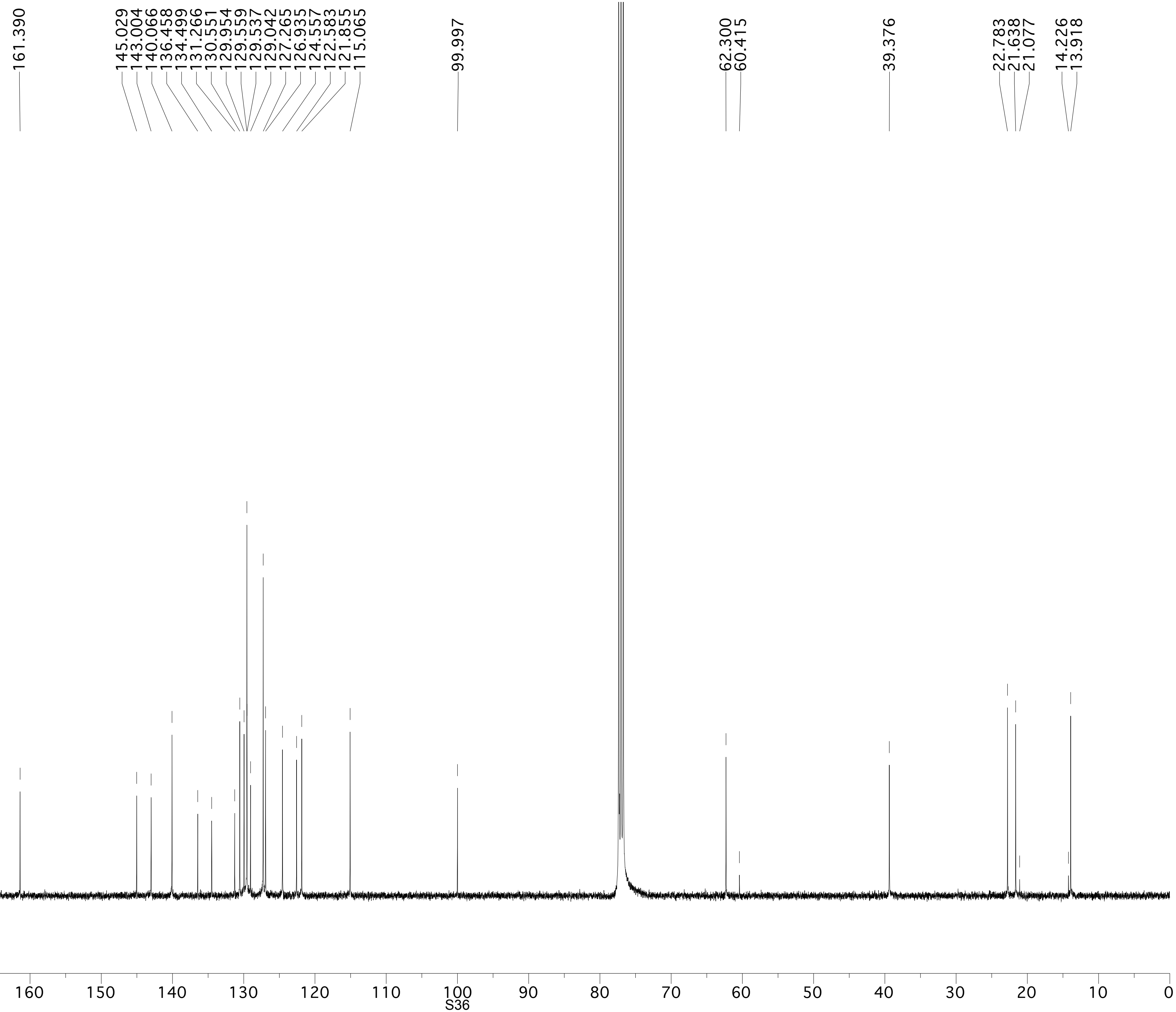
169.892
161.390
145.029
143.004
140.066
136.458
134.499
131.266
130.551
129.954
129.559
129.537
129.042
127.265
126.935
124.557
122.583
121.855
115.065
99.997
62.300
60.415
39.376
22.783
21.638
21.077
14.226
13.918
00 190 180 170 160 150 140 130 120 110 100 90 80 70 60 50 40 30 20 10 0

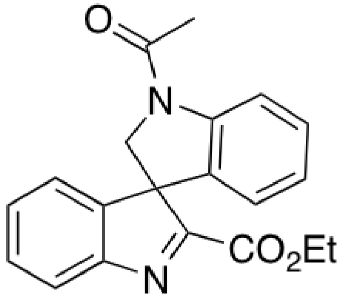
O
N
CO2Et
N

11

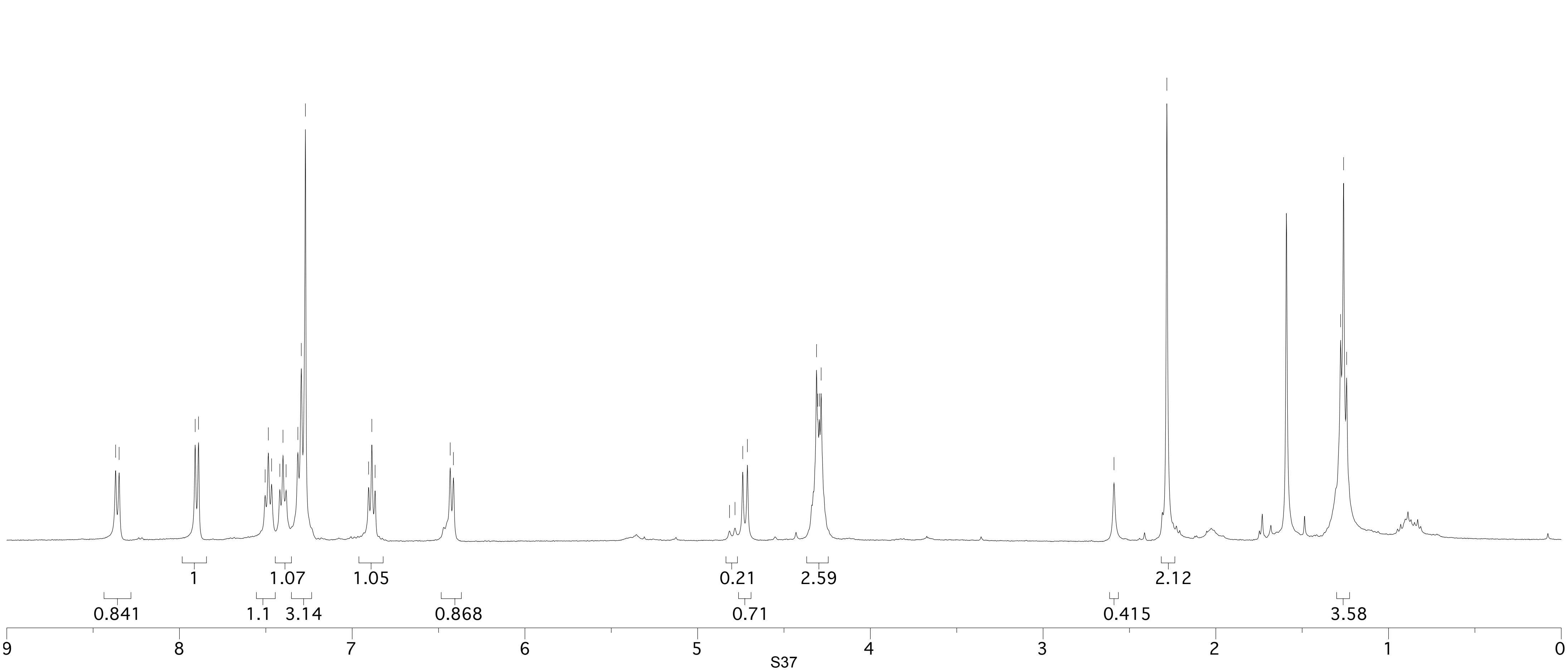
8.370
8.350
7.909
7.890
7.504
7.485
7.467
7.419
7.401
7.383
7.315
7.295
7.271
6.905
6.886
6.867
6.433
6.414
4.815
4.784
4.739
4.712
4.312
4.305
4.295
4.285
2.590
2.283
1.278
1.260
1.242
1
1.07
1.05
0.21
2.59
2.12
0.841
1.1
3.14
0.868
0.71
0.415
3.58
9
8
7
6
5
4
3
2
1
0

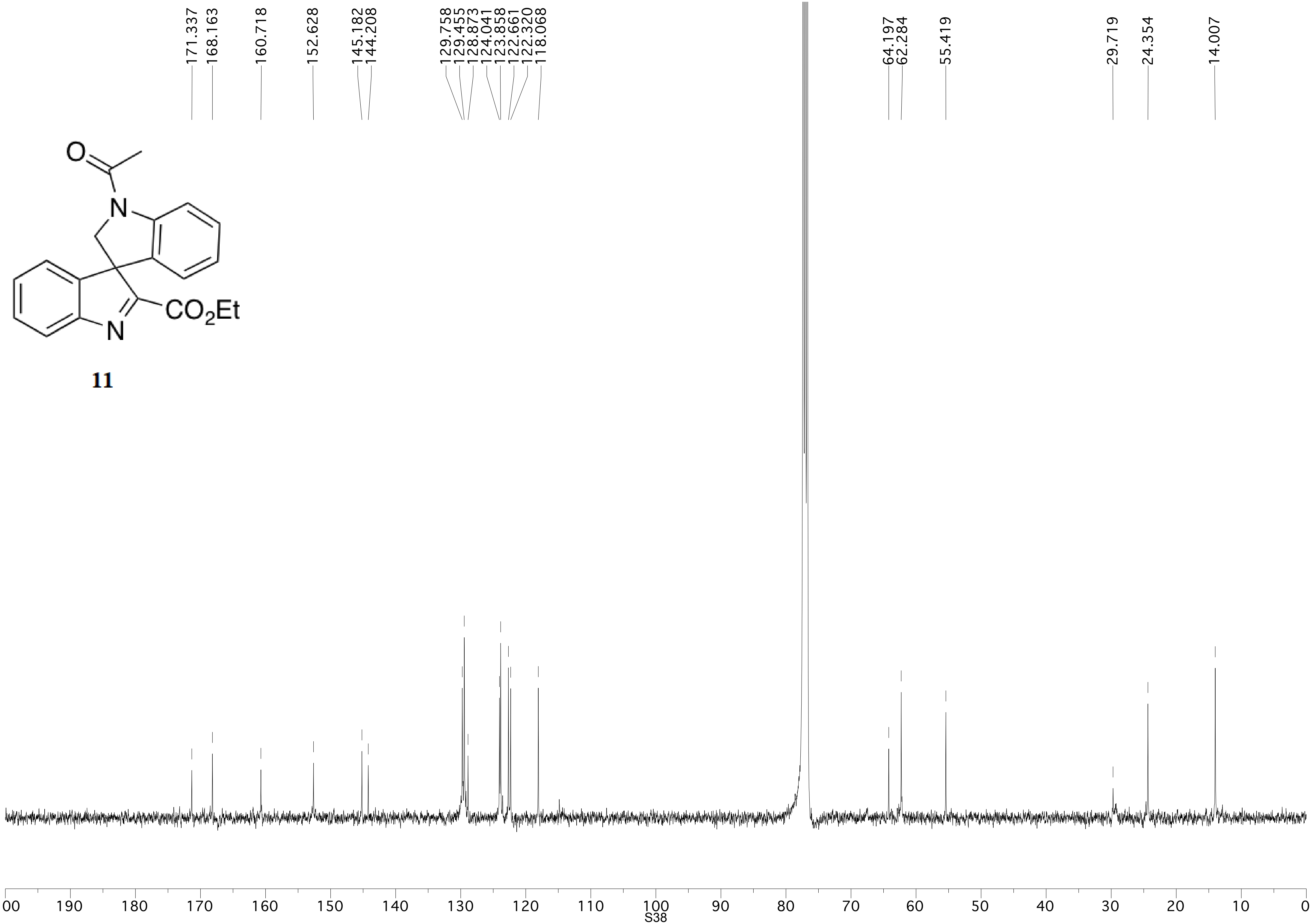
171.337
168.163
160.718
152.628
145.182
144.208
129.758
129.455
128.873
124.041
123.858
122.661
122.320
118.068
64.197
62.284
55.419
29.719
24.354
14.007
O
N
N
CO2Et
11
00
190
180
170
160
150
140
130
120
110
100
90
80
70
60
50
40
30
20
10
0

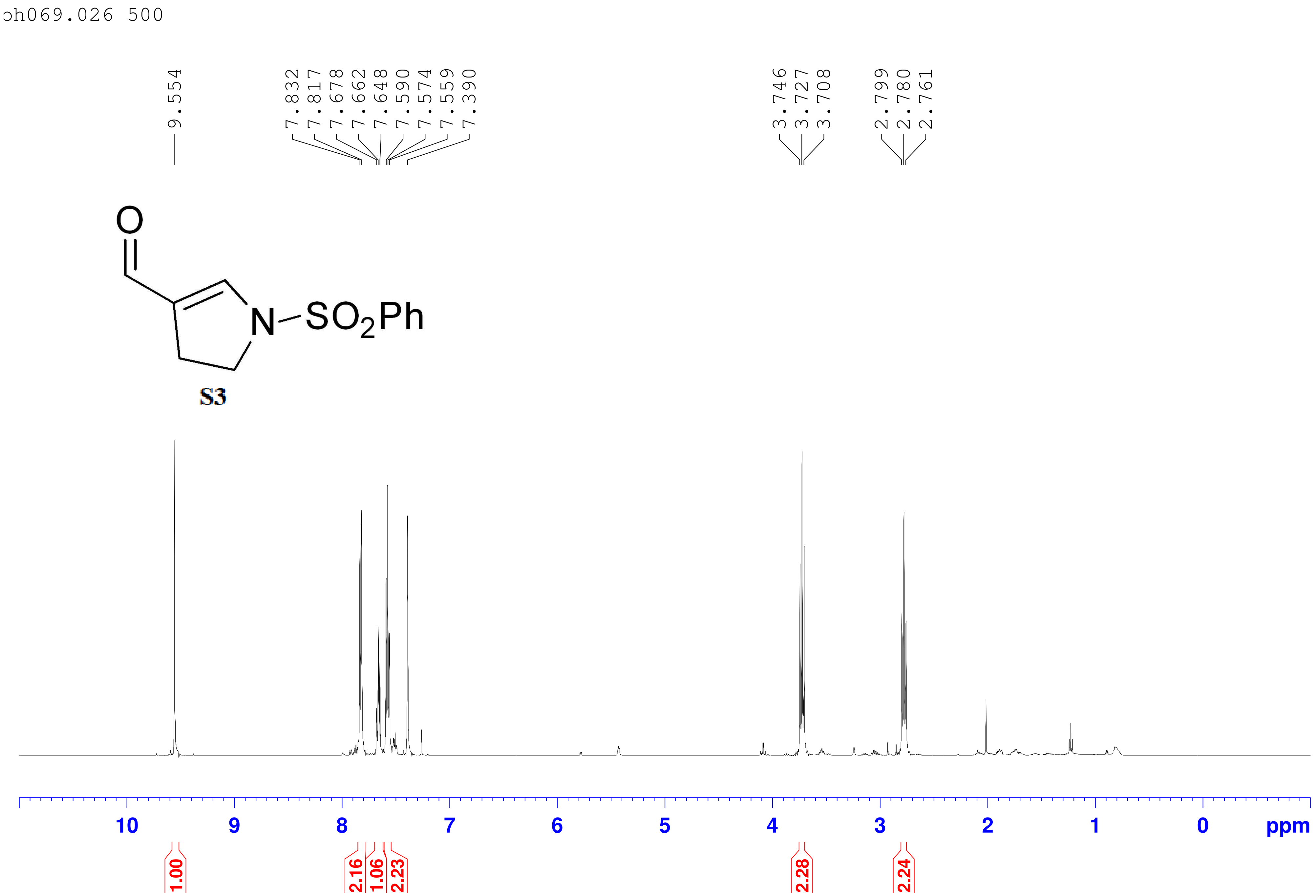
h069.026 500
9.554
7.832
7.817
7.678
7.662
7.648
7.590
7.574
7.559
7.390
3.746
3.727
3.708
2.799
2.780
2.761
O
N
SO2Ph
S3
10
9
8
7
6
5
4
3
2
1
0
ppm
1.00
2.16
1.06
2.23
2.28
2.24

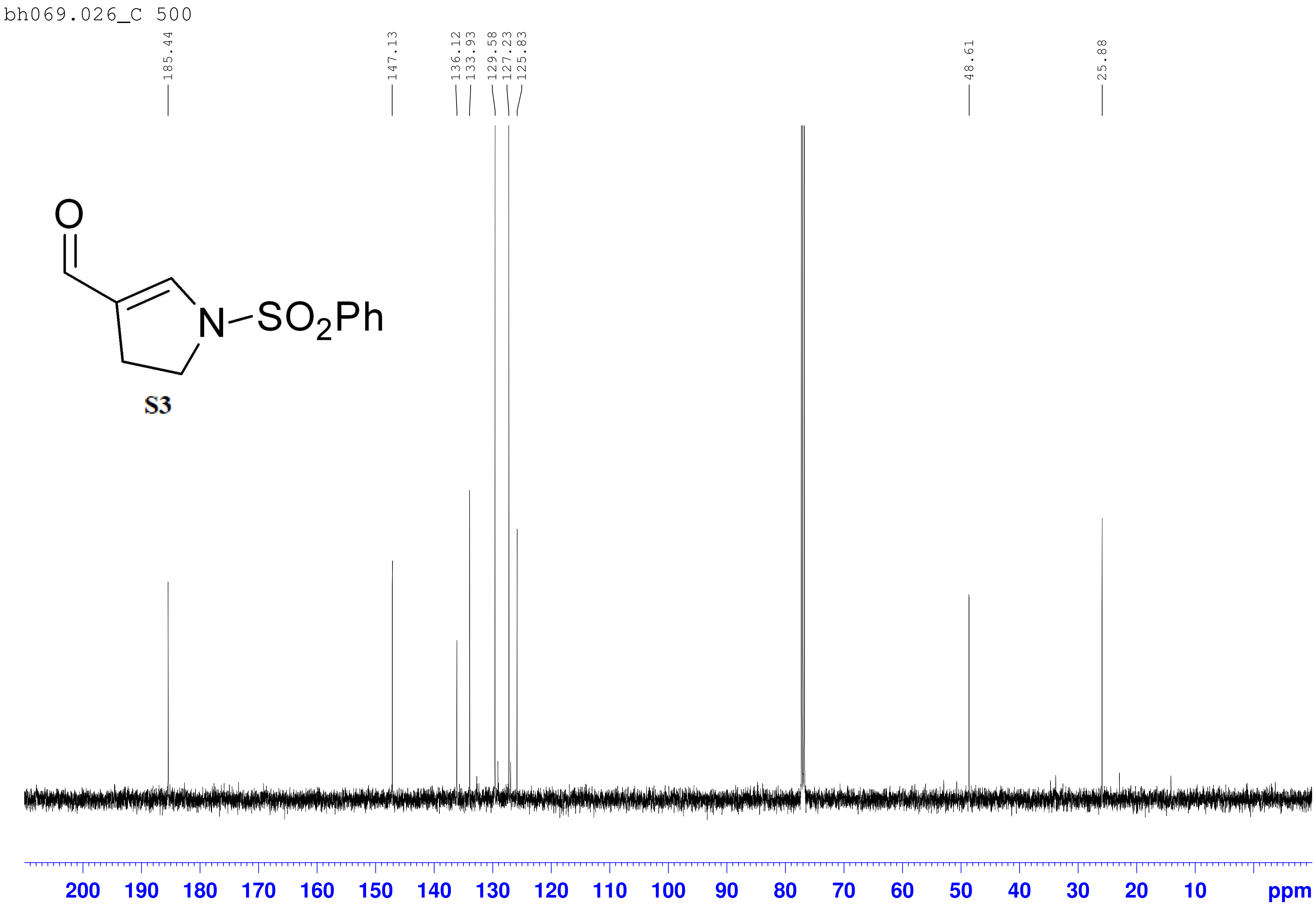
bh069.026_C 500
185.44
147.13
136.12
133.93
129.58
127.23
125.83
48.61
25.88
O
N–SO2Ph
S3
200 190 180 170 160 150 140 130 120 110 100 90 80 70 60 50 40 30 20 10 ppm

bh069.028.15-40 400b

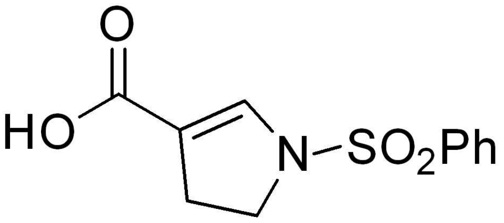

21

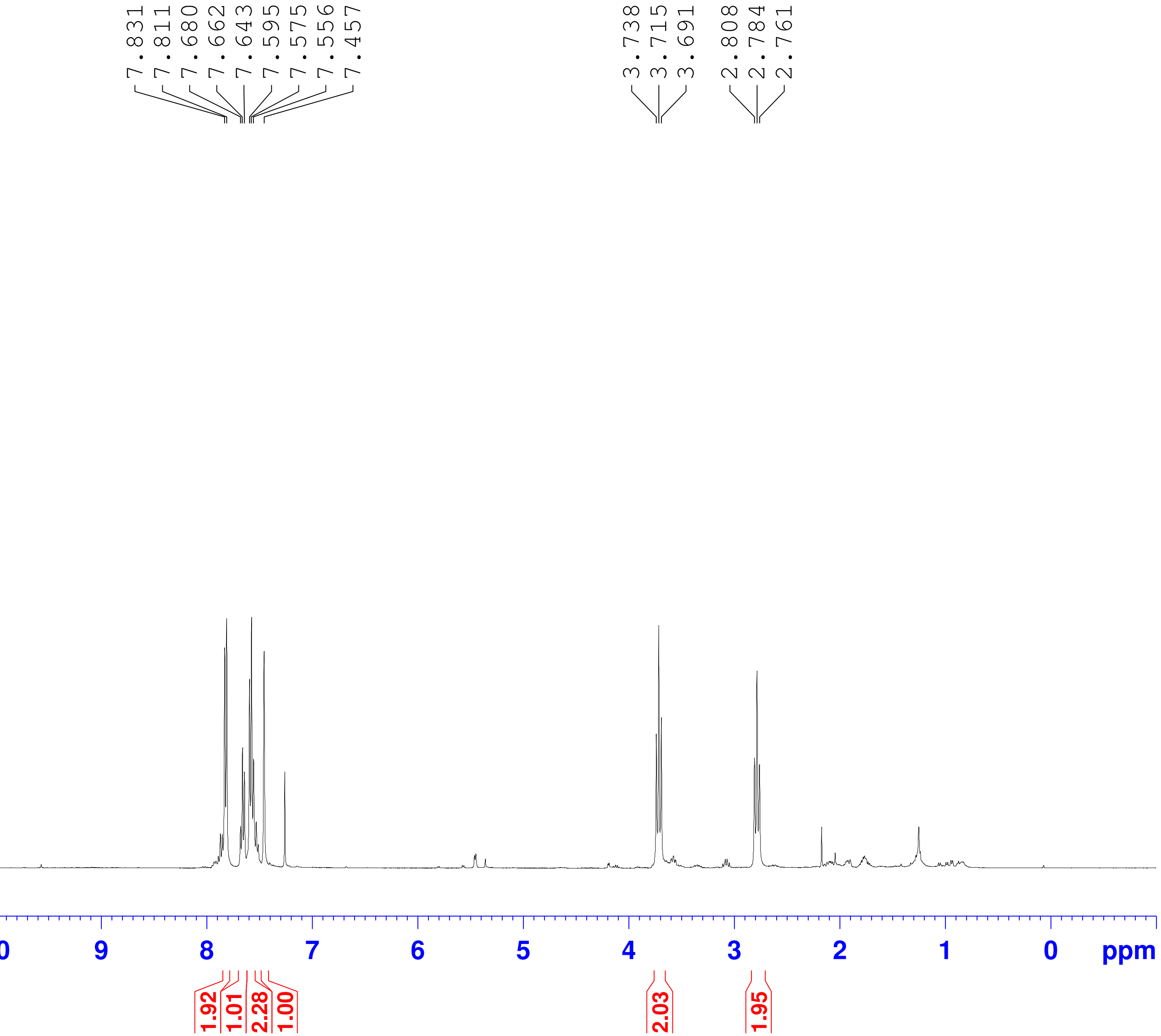

bh069.028.15-40_C 400b

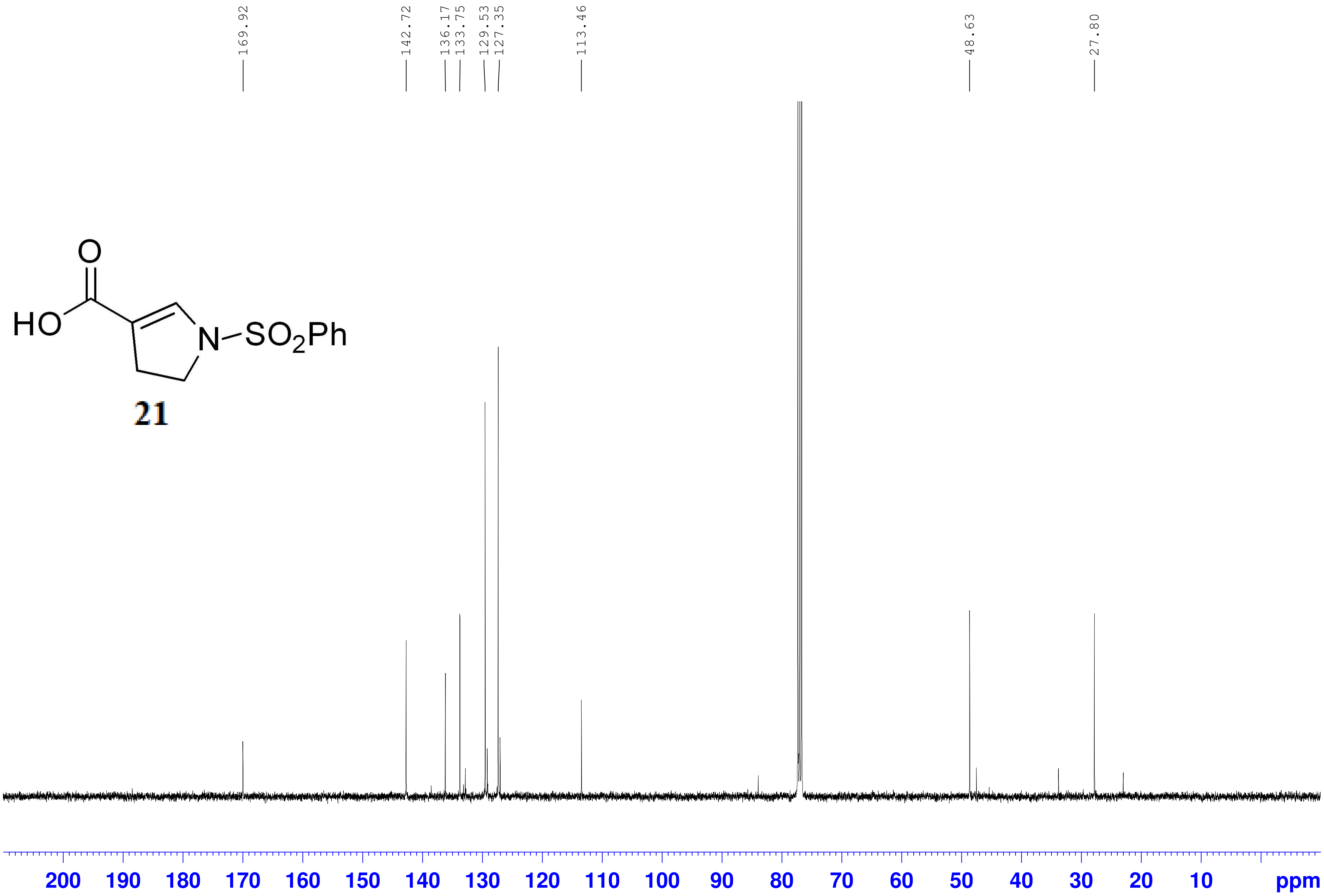

bh090.040 400B

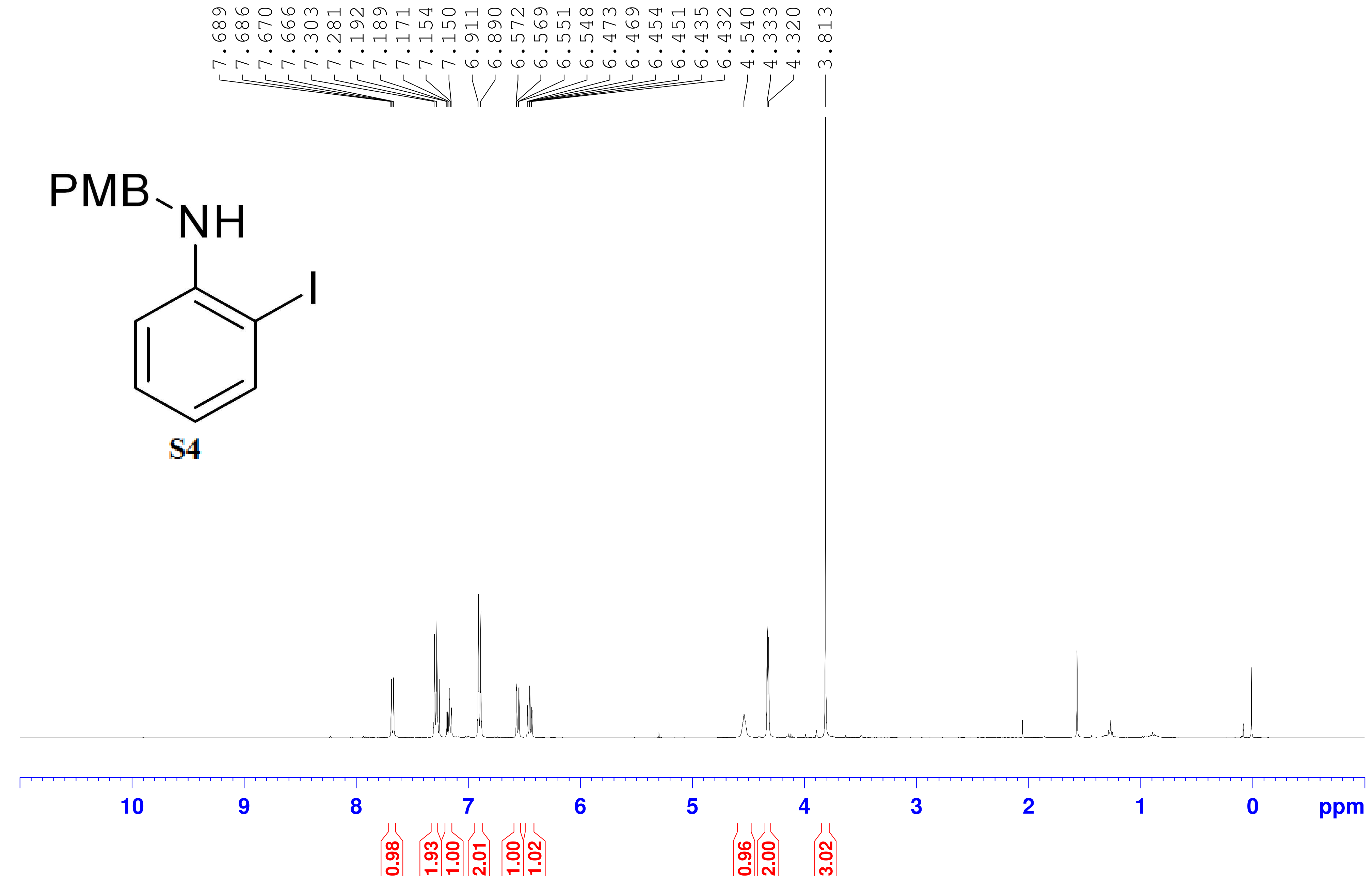

bh090.040_C 400B

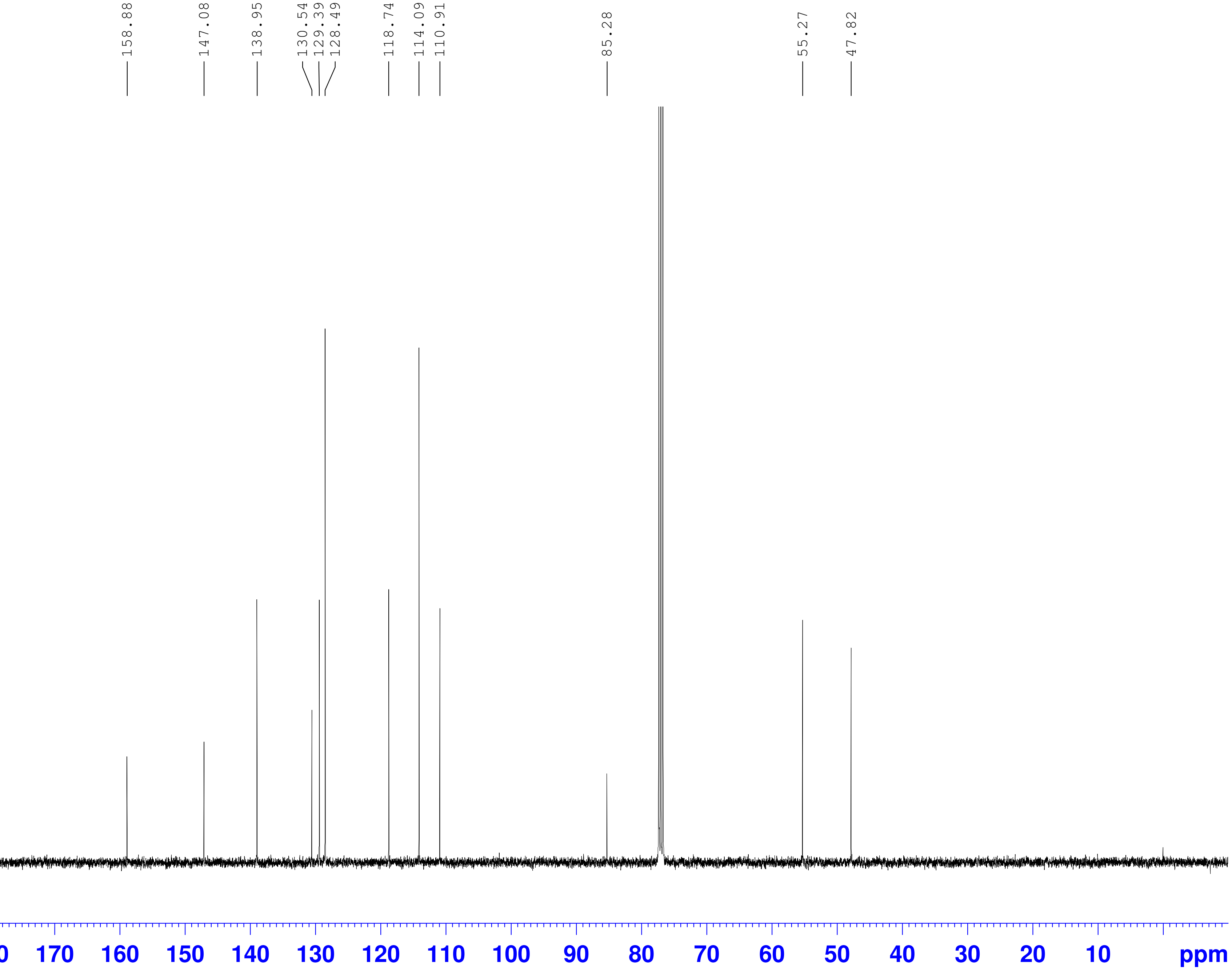

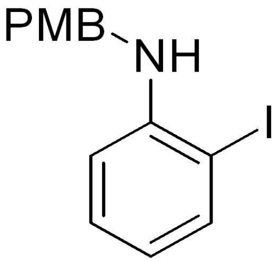

S4

bh090.050 500

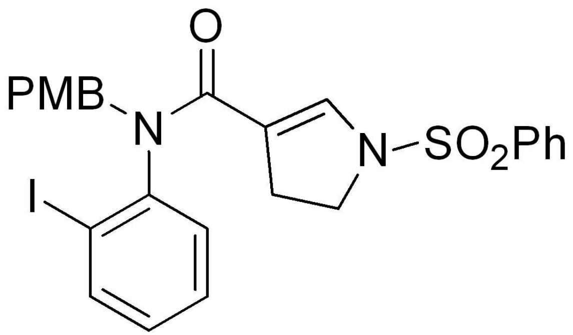

22

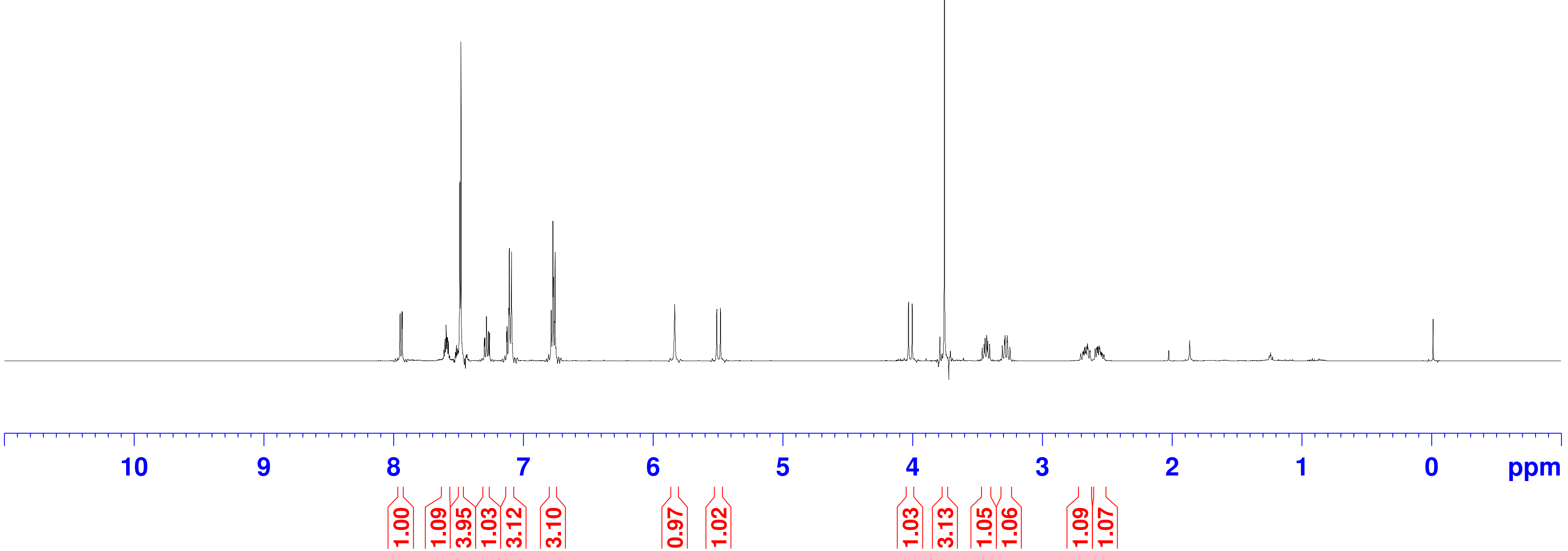

bh090.050_C 500

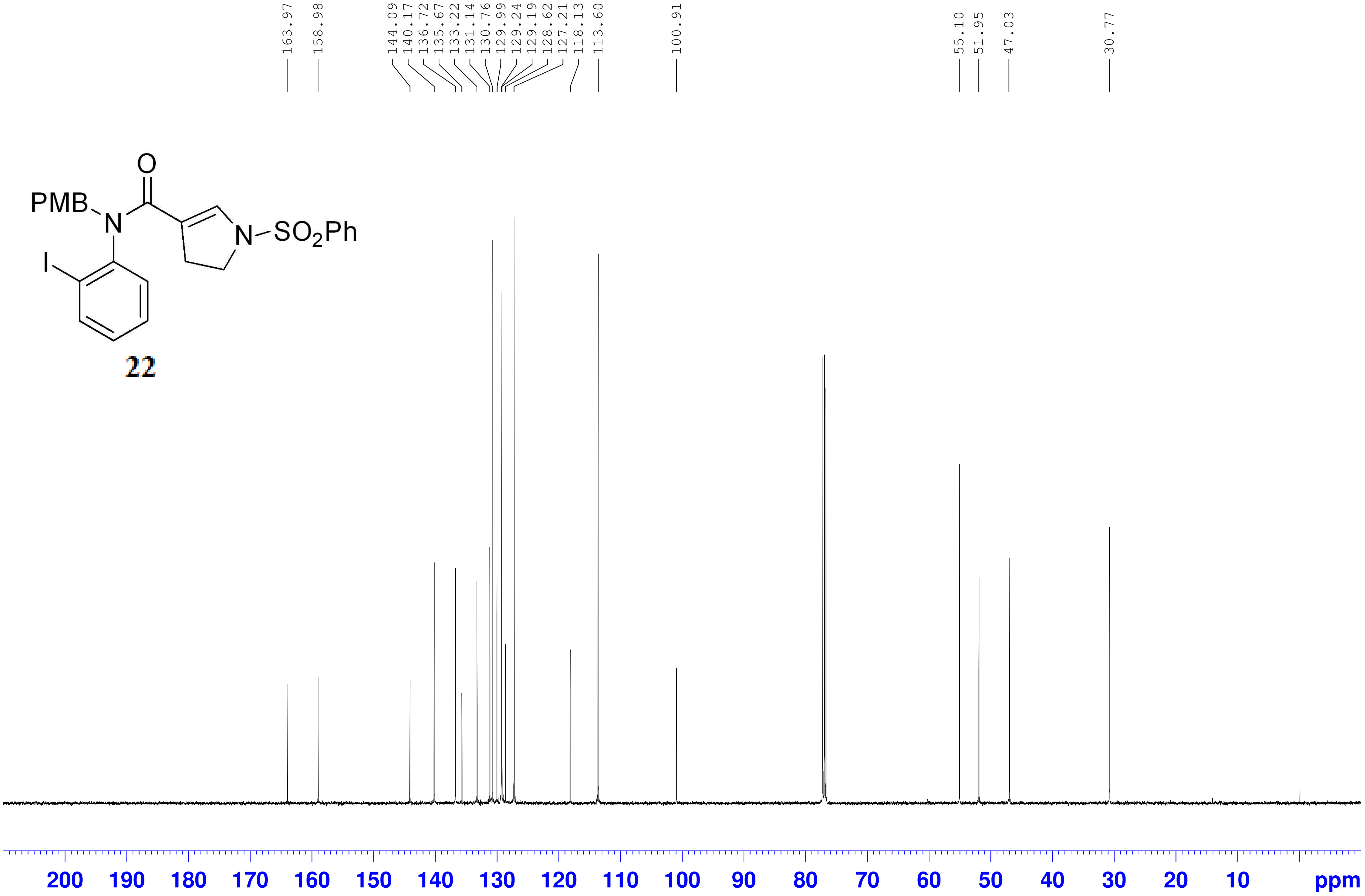

bh090.020.7-11 400b

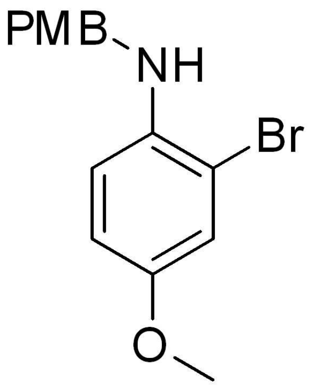

S5

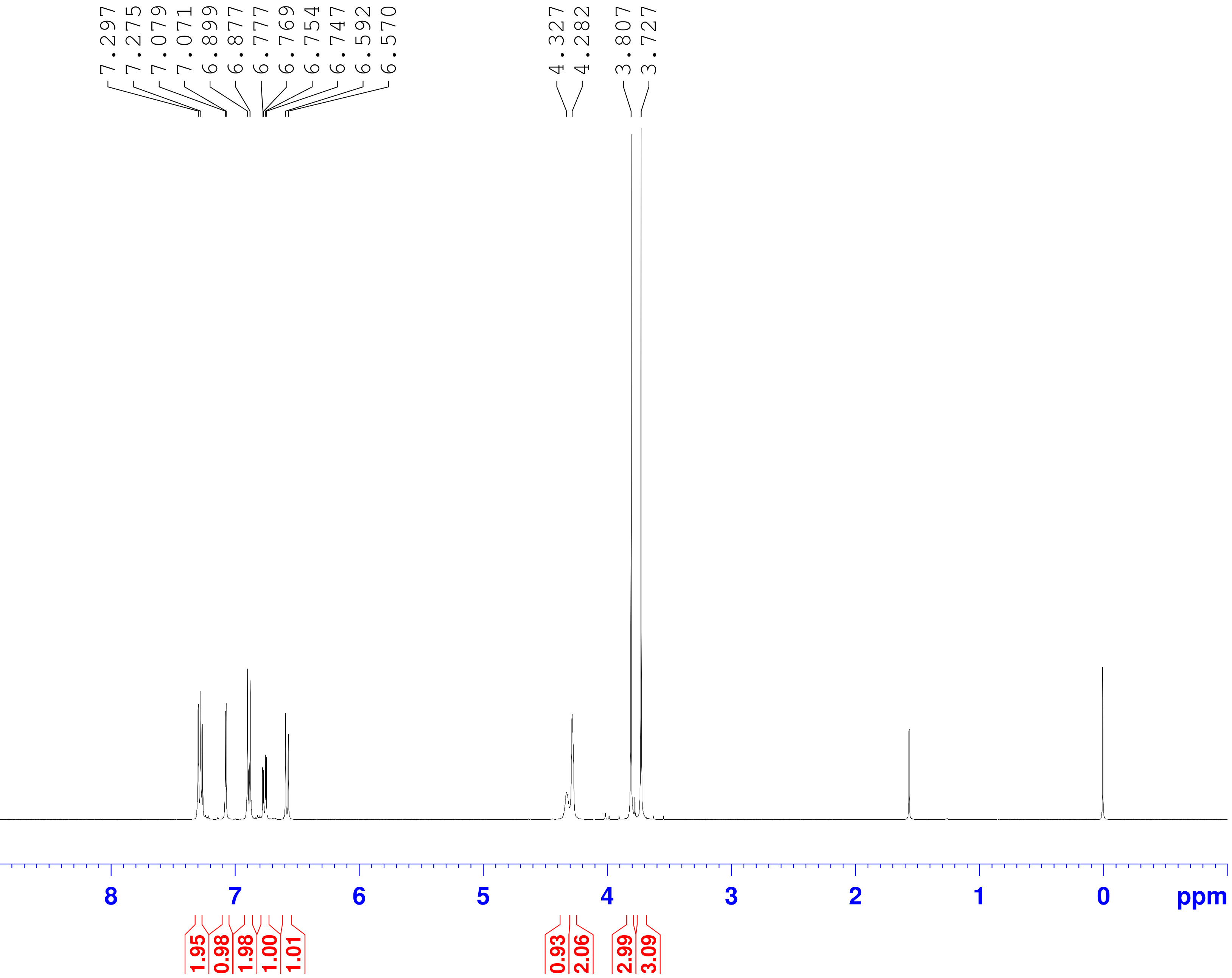

bh090.012.36-42_C 500 cdcl3 6-29-11

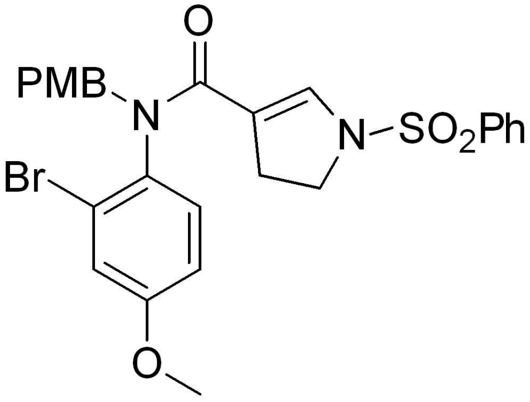

S5

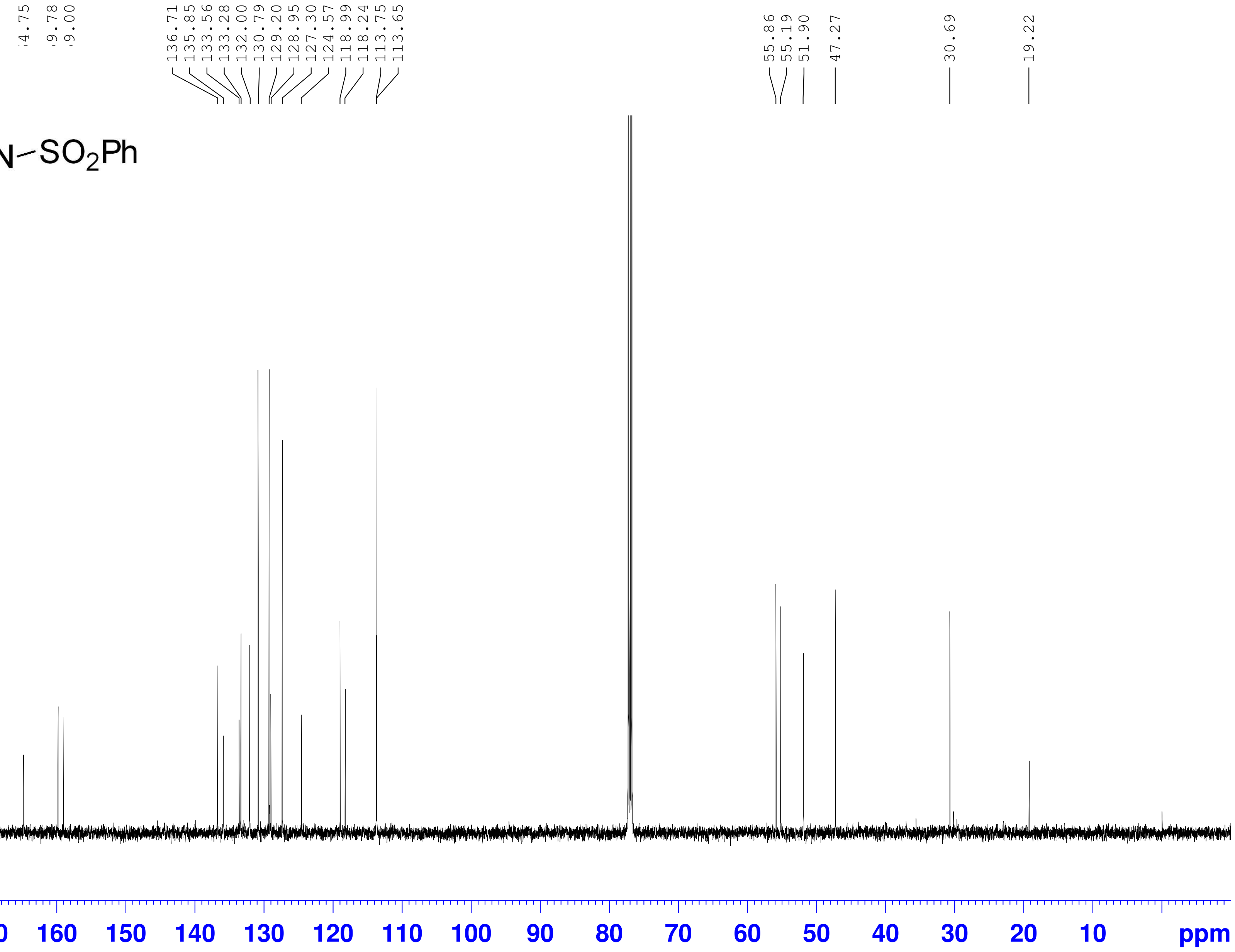

bh090.012.36-42 500

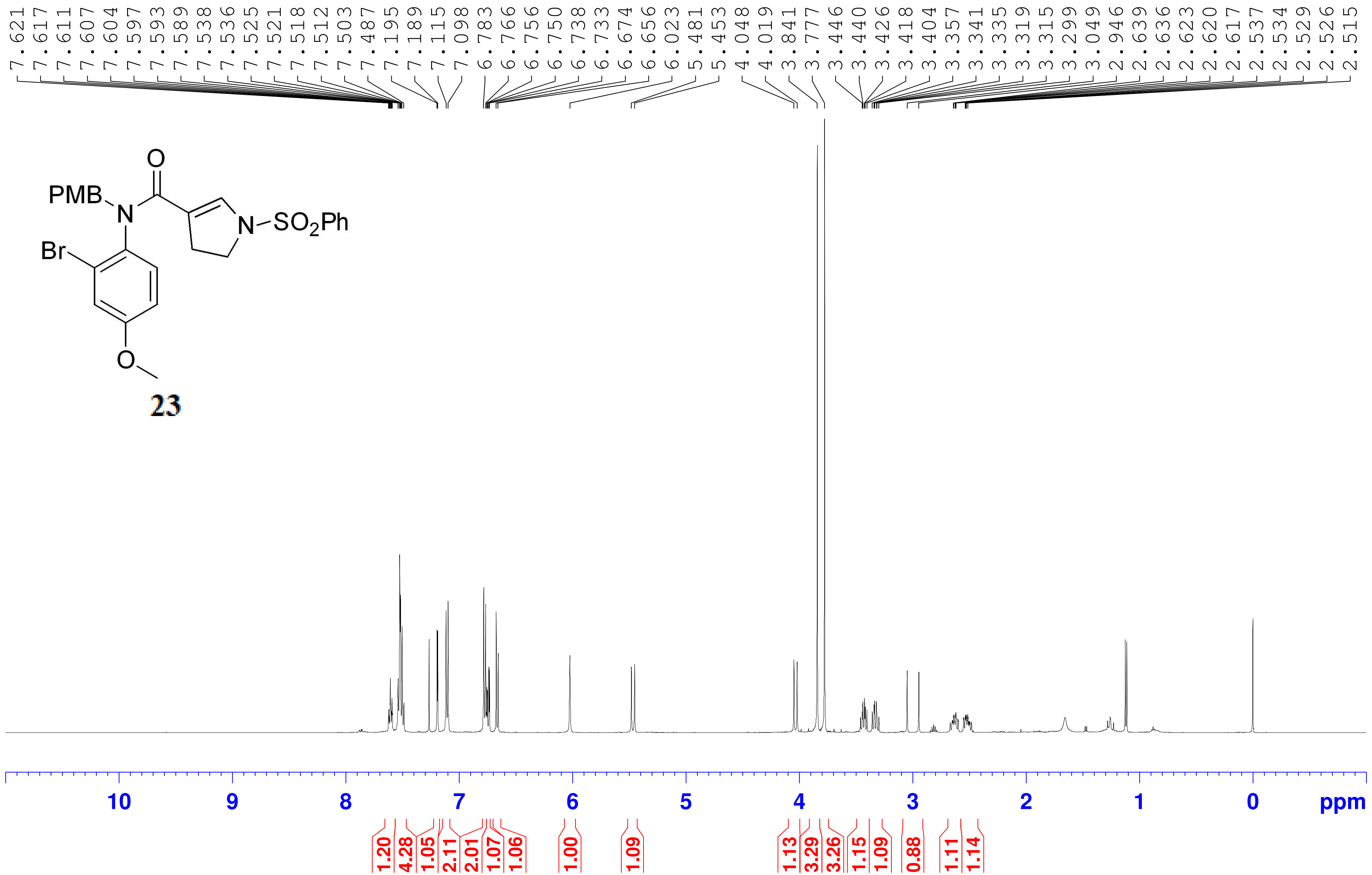

bh090.012.36-42_C 500 cdcl3 6-29-11

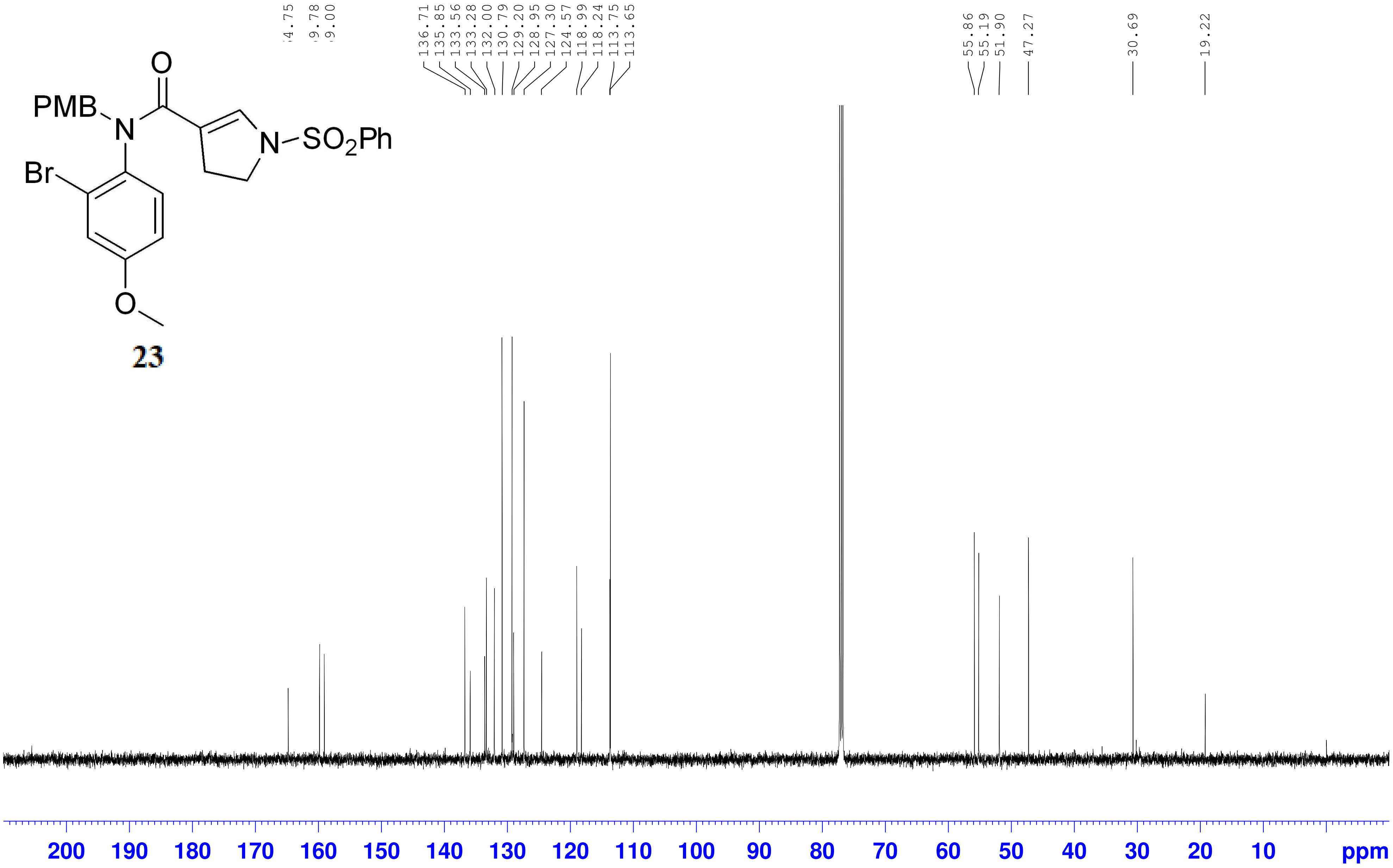

23

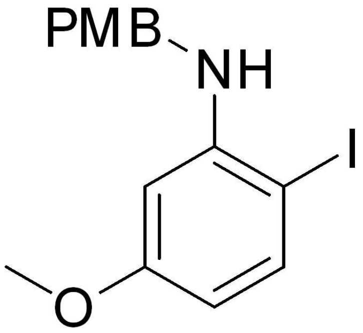
PMB
NH
I
O

S6

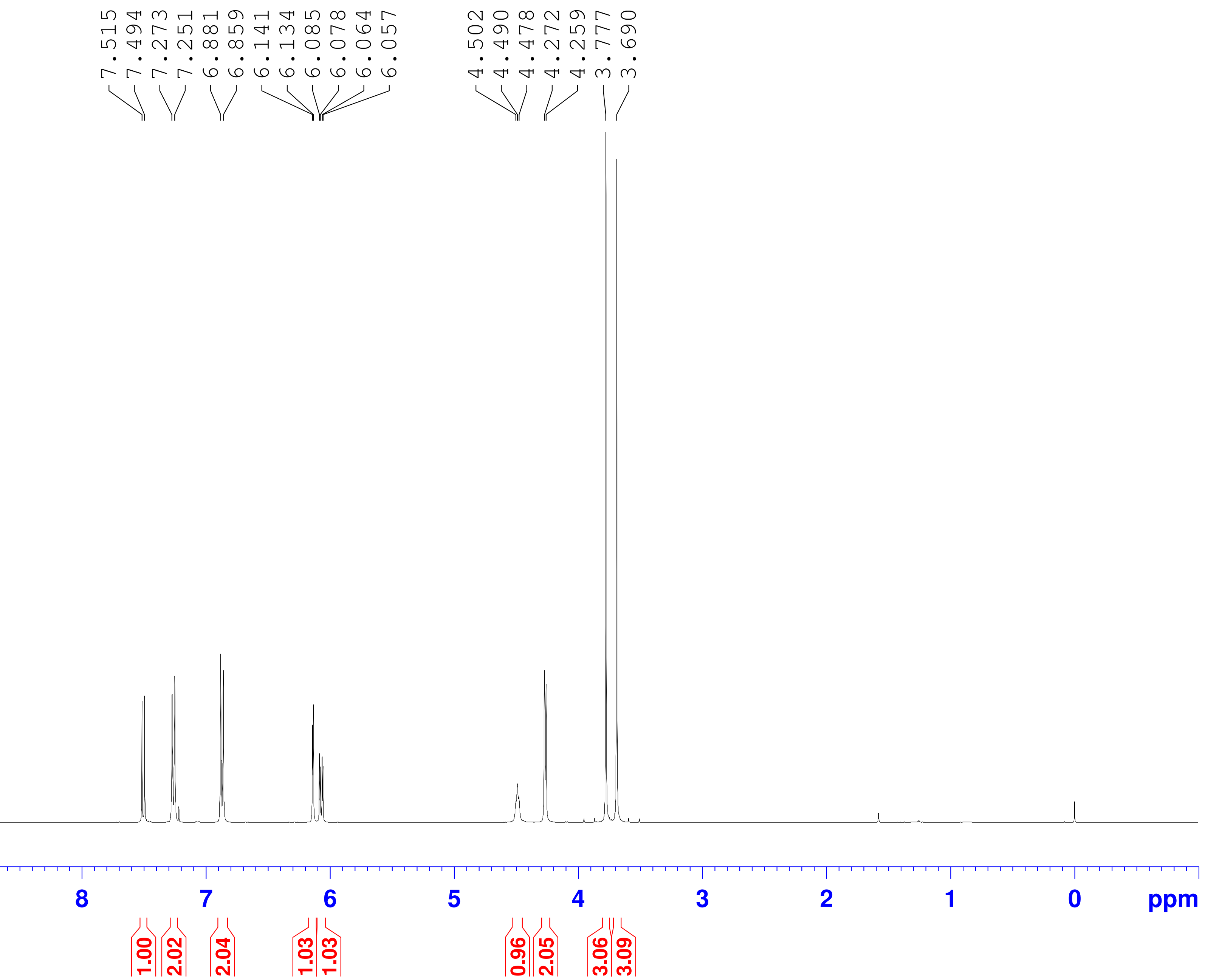
bh090.01.14-17 400b
7.515
7.494
7.273
7.251
6.881
6.859
6.141
6.134
6.085
6.078
6.064
6.057
4.502
4.490
4.478
4.272
4.259
3.777
3.690
10
9
8
7
6
5
4
3
2
1
0
ppm
1.00
2.02
2.04
1.03
1.03
0.96
2.05
3.06
3.09

bh090.01.14-17_C 400b

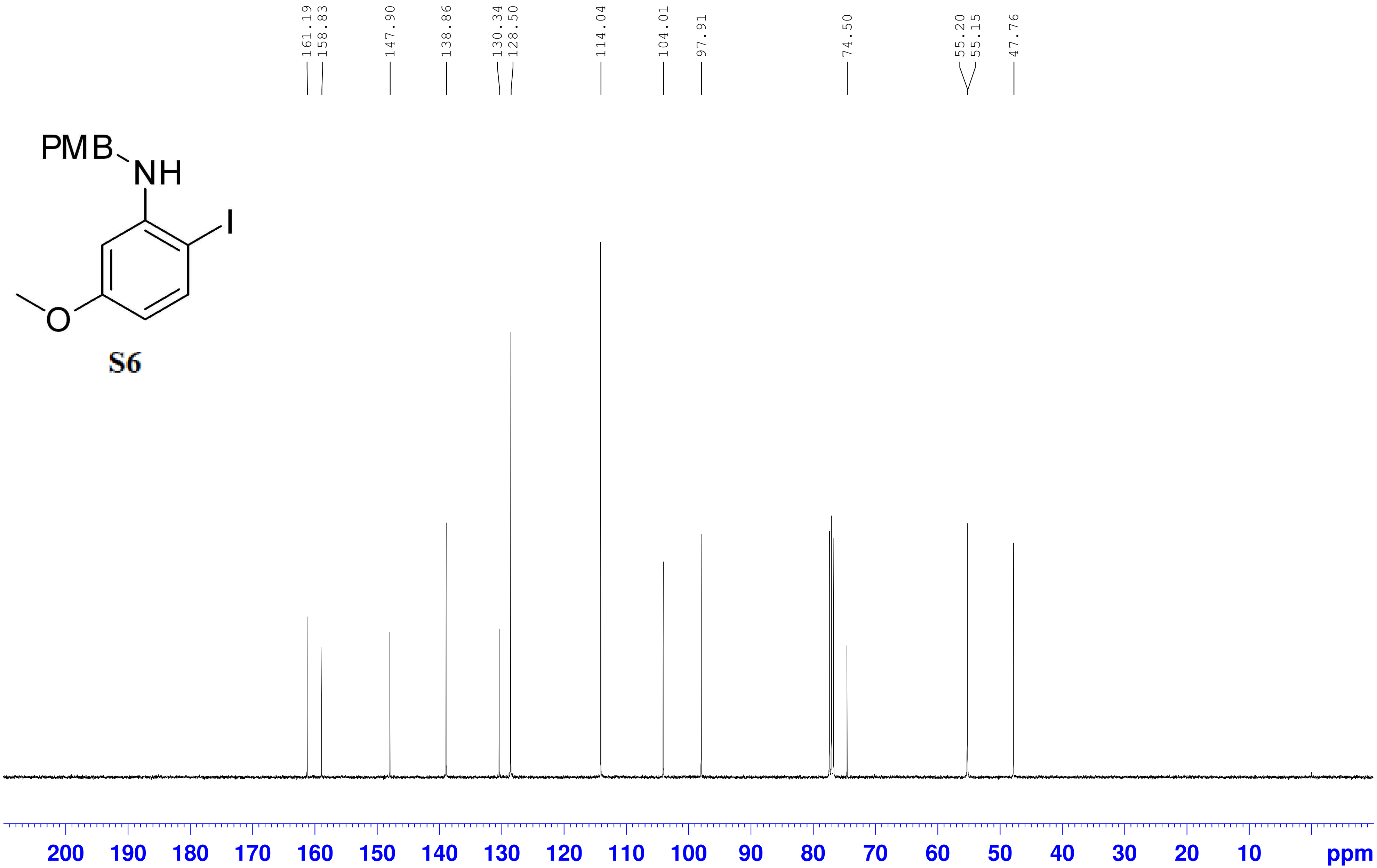

bh090.019 500

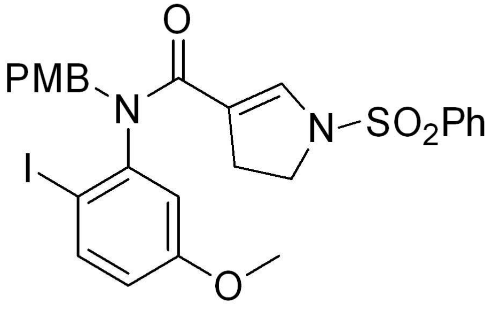

24

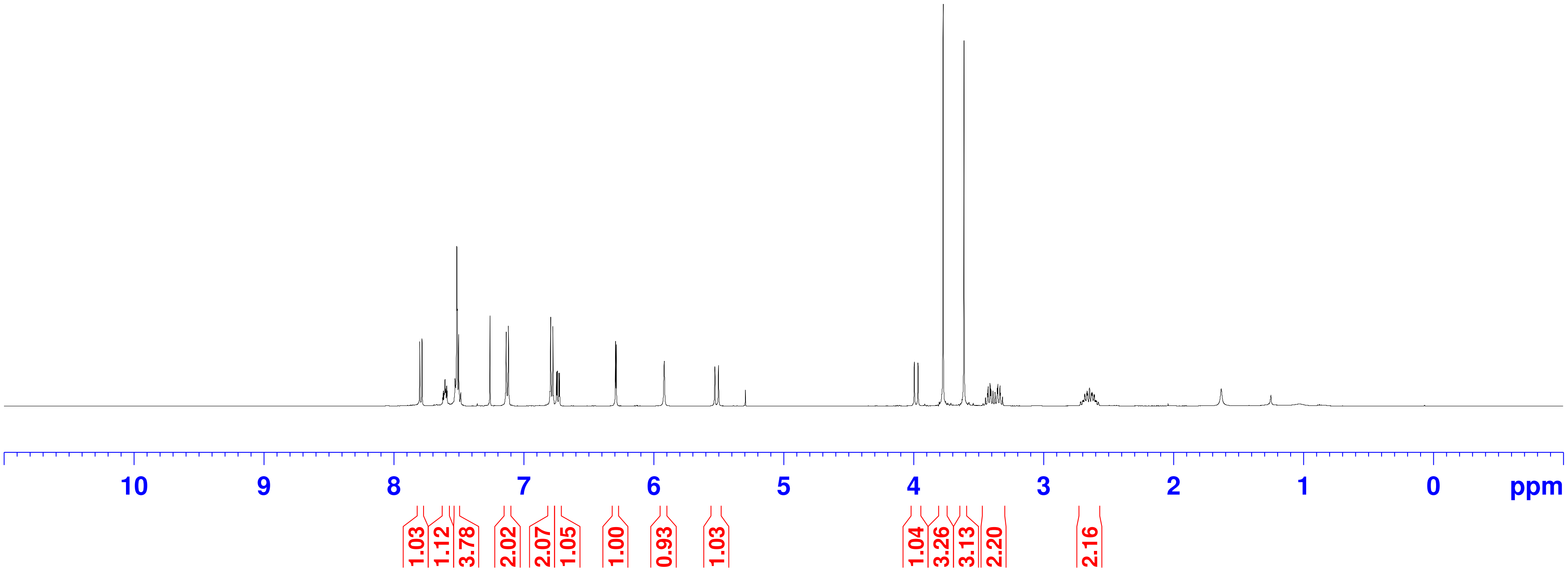

bh090.019_C 500

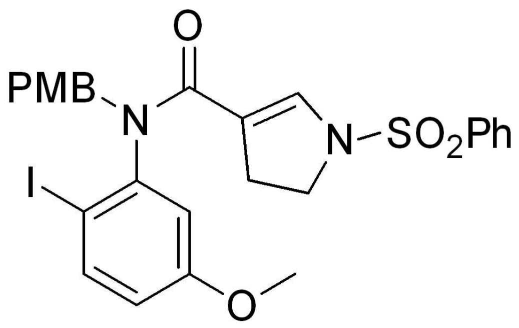

24

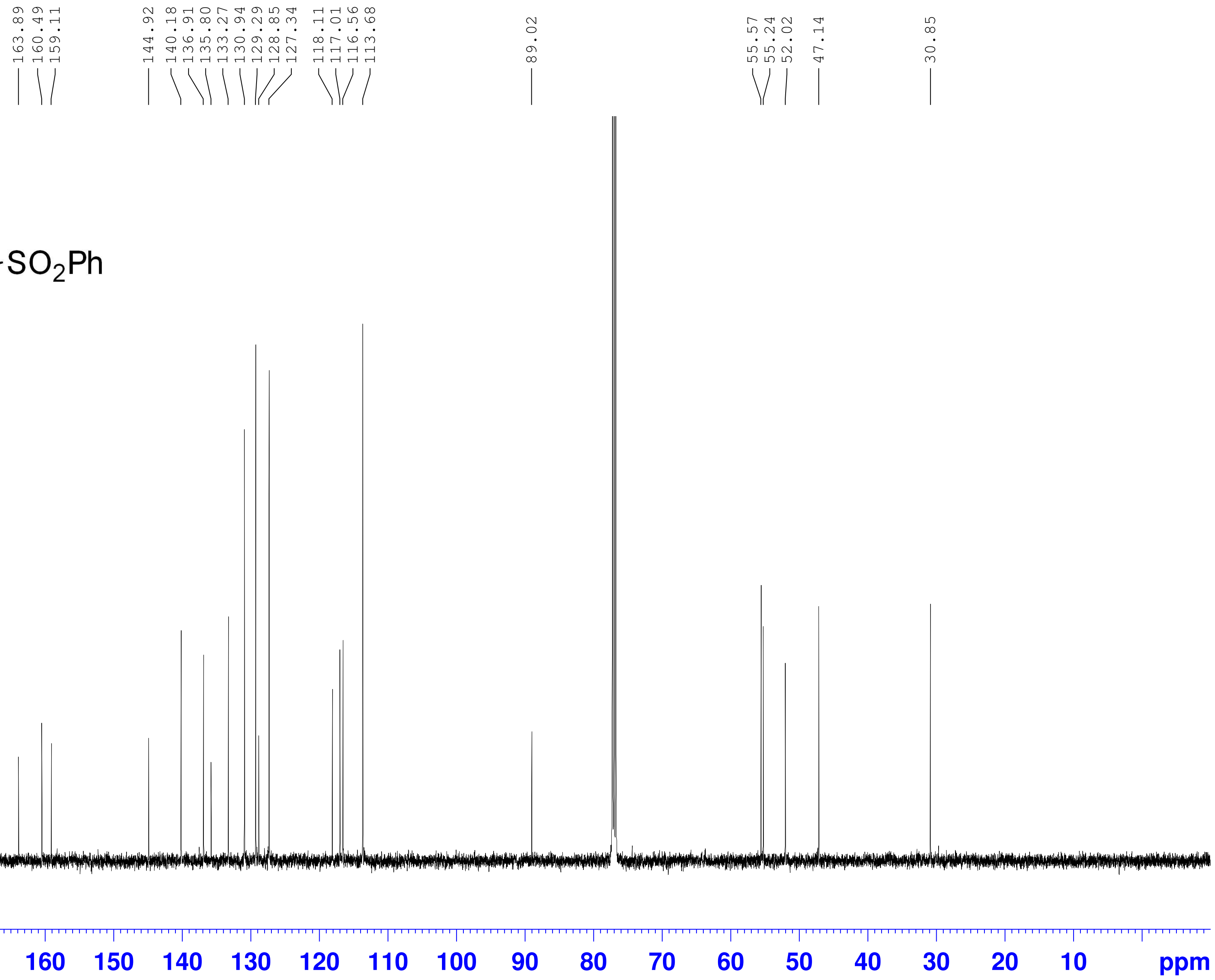

bh090.059.35-42 500

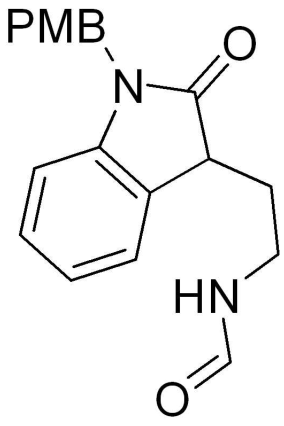

25

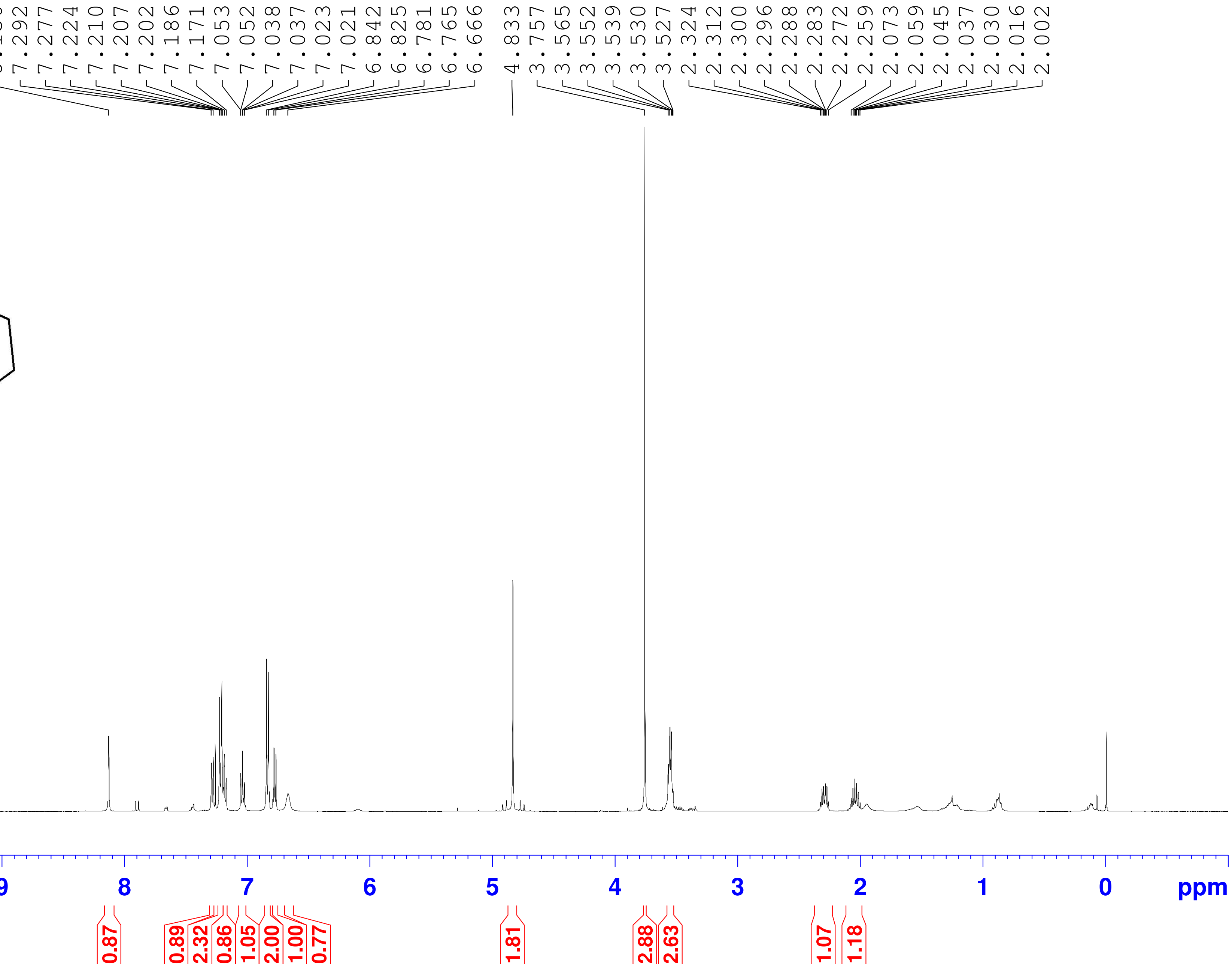

bh090.059.35-42_C 500

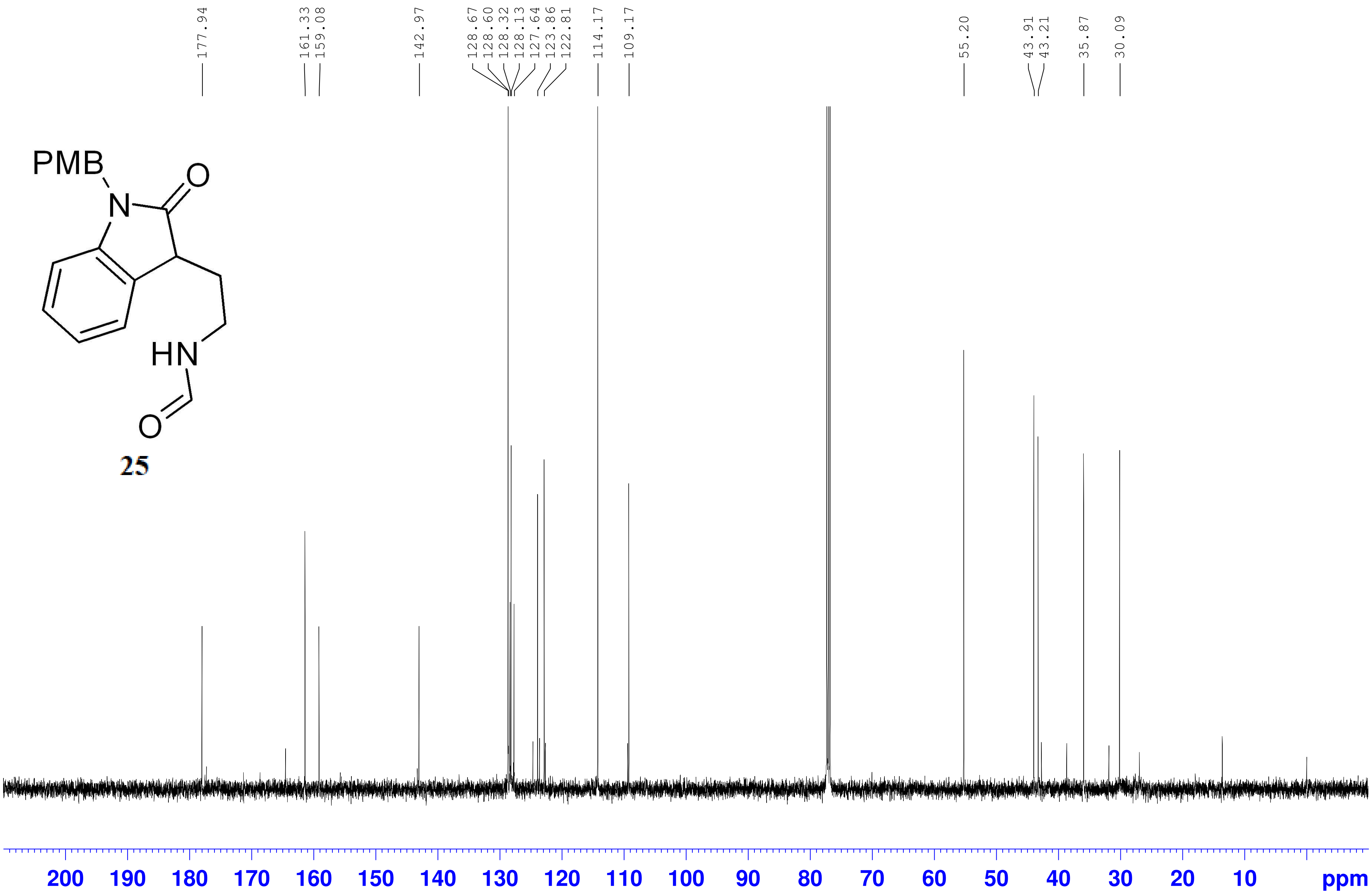

25

bh090.024.10-15 500

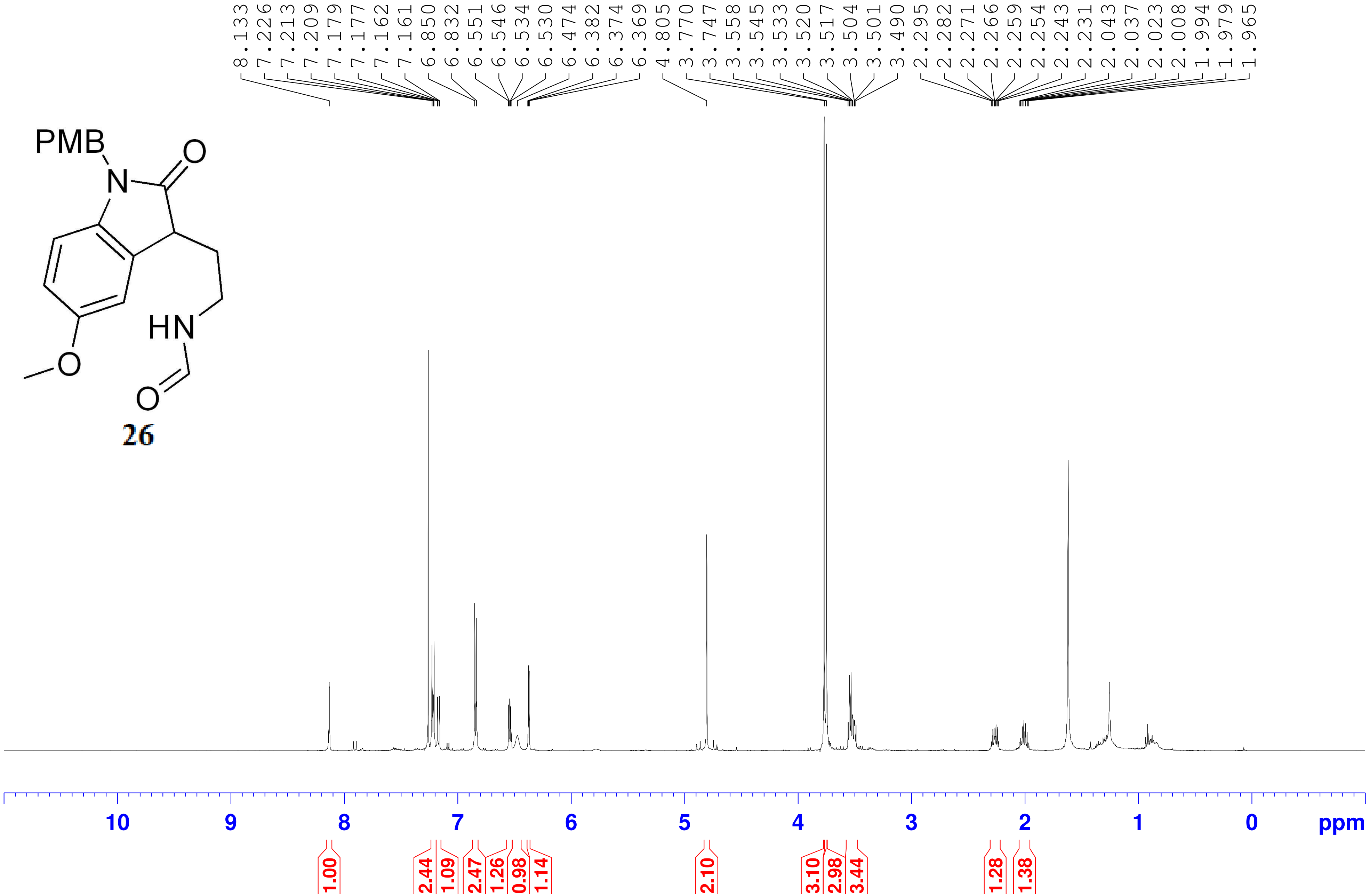

26

bh090.024.10-15_C 500

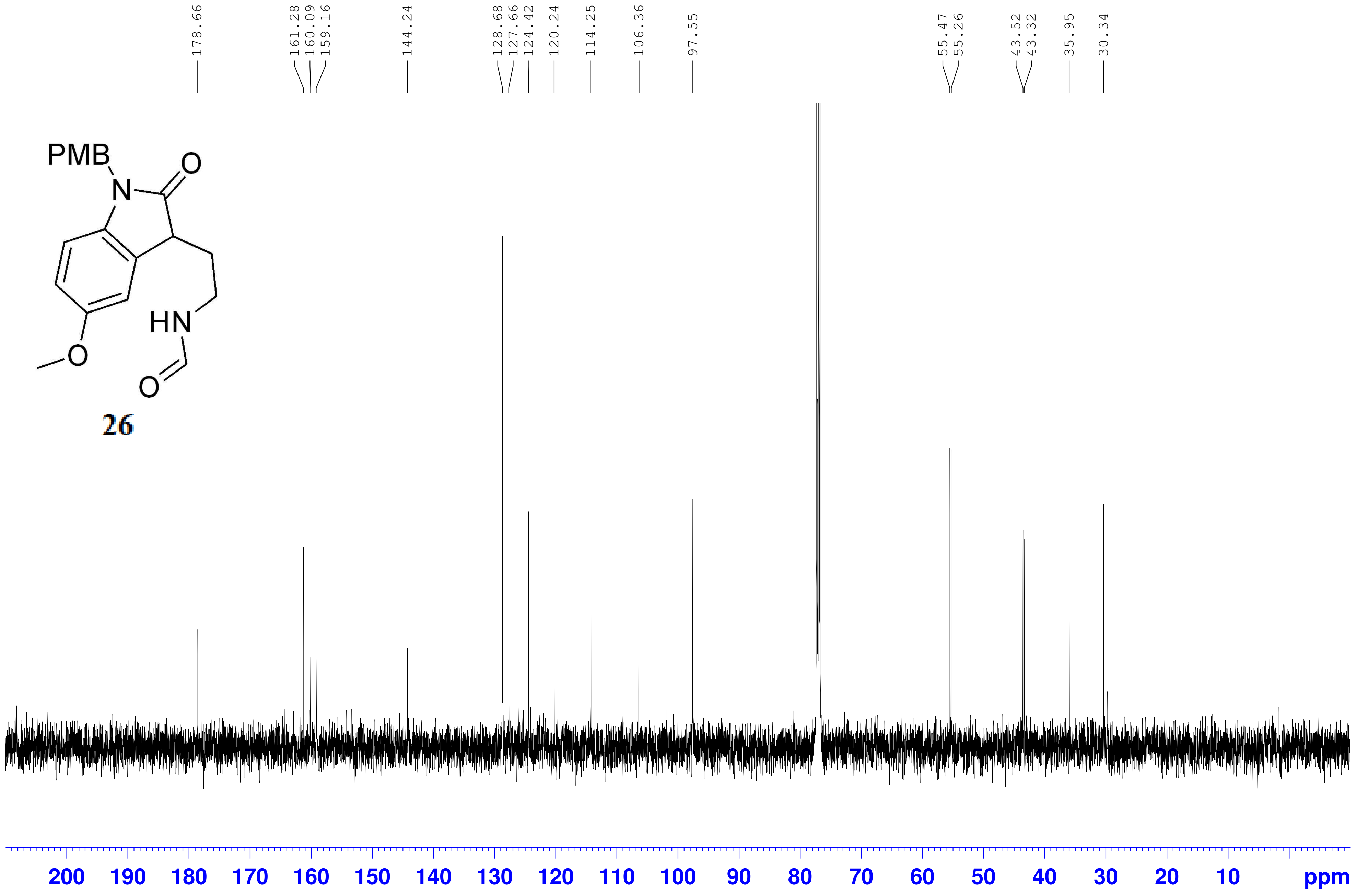

26

bh090.022.23-28 500

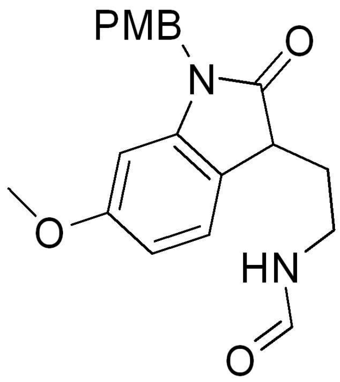

27

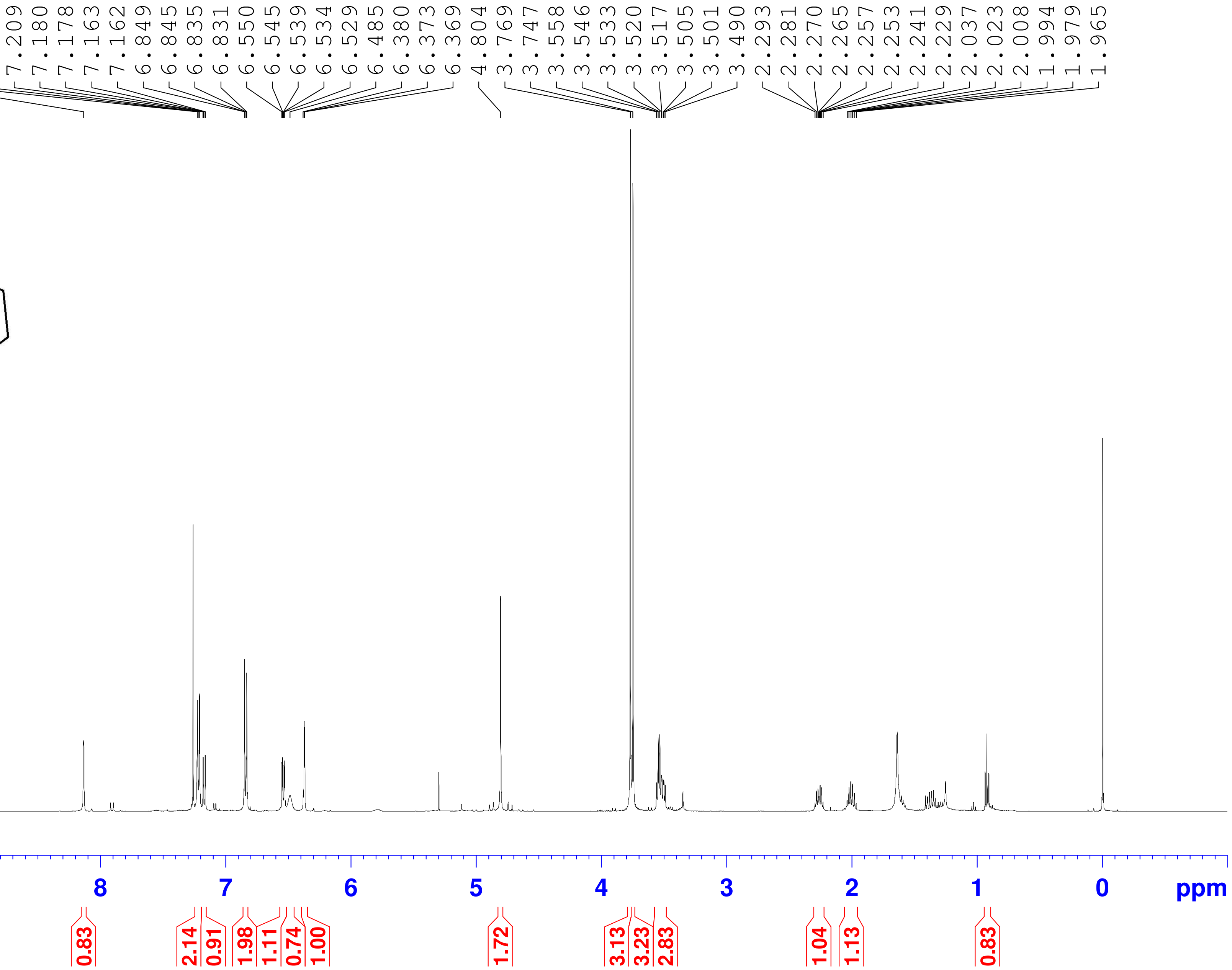

bh090.022.23-28_C 500

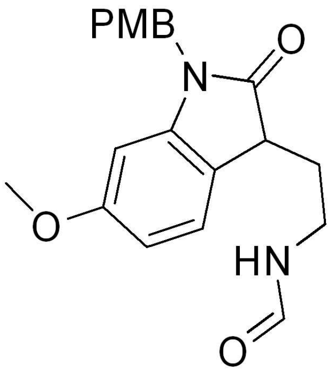

27

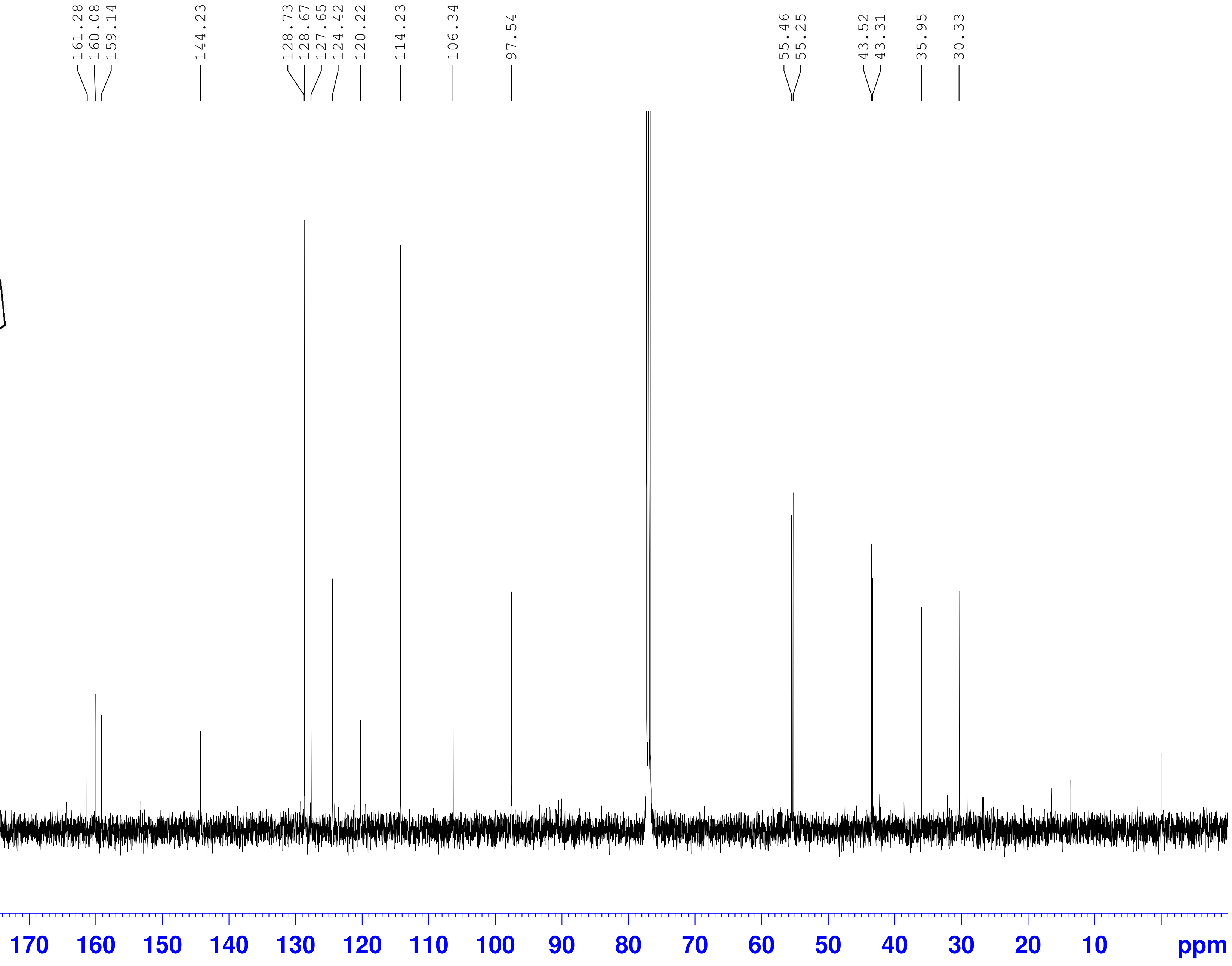

Supplement: File 1 — Experimental procedures, compound characterization data, and copies of NMR spectra. [file Beilstein_J_Org_Chem-11-1649-s001.pdf]
